# Paramagnetic Cage-Type Co(II) Complexes of Chiral Macrocycles: Enantio- and Size-Selective Binding of Guest Molecules

**DOI:** 10.1021/acs.inorgchem.4c03956

**Published:** 2025-02-24

**Authors:** Jan Janczak, Jerzy Lisowski

**Affiliations:** † Department of Chemistry, University of Wrocław, 14 F. Joliot-Curie, Wrocław 50-383, Poland; ‡ Institute of Low Temperature and Structure Research, Polish Academy of Sciences, Okólna 2 str., Wrocław 50-422, Poland

## Abstract

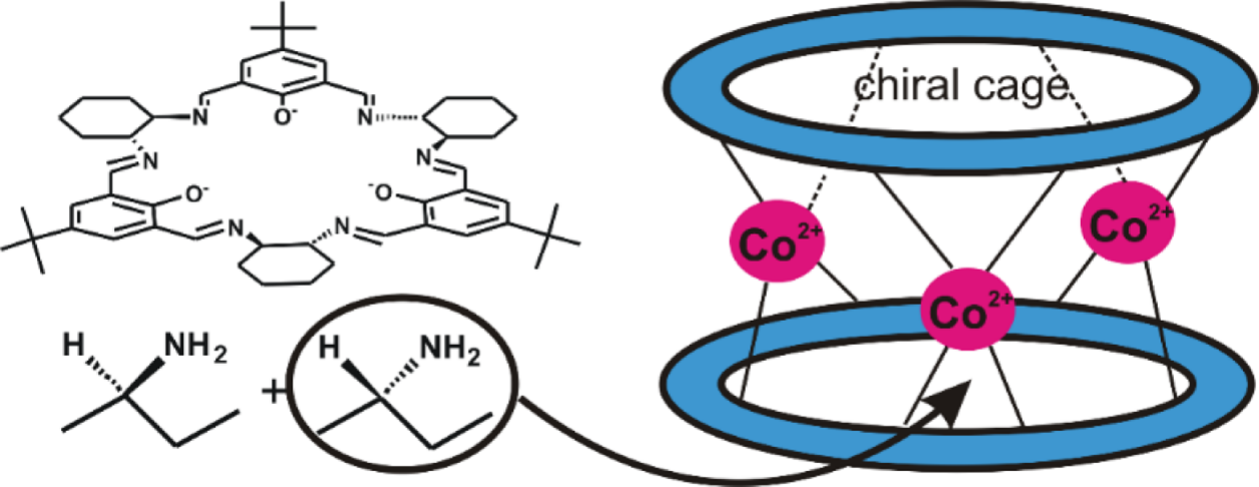

Two enantiomers of the cage-type complex, **[Co_3_L*^R^*_2_]** and **[Co_3_L*^S^*_2_]** of a large hexaazatriphenolic [3 + 3] macrocyclic imine **L**, have been synthesized and characterized on the basis of NMR, CD, and ESI MS spectra. The X-ray crystal structures of **[Co_3_L_2_]** crystalline forms reveal two macrocycles of cone shape stitched together by three Co(II) ions, forming a barrel-shaped molecule with a central void. Because of the limited size of the **[Co_3_L_2_]** cavity and the enantiopure nature of these enantiomeric complexes, both size-selective and enantioselective binding of guest molecules are observed. In the case of chiral guests, the interaction with paramagnetic Co(II) centers leads to an effective NMR enantiodifferentiation of the signals of guest molecules, even at host:guest ratios as low as 1:200. The tight binding of prochiral guest molecules such as ethanol and isopropanol within the chiral cavity results in the splitting of enantiotopic methylene and methyl signals. The *dc* magnetic data for **[Co_3_L_2_]** are in accord with the presence of high-spin Co(II) ions, and the *ac* susceptibility data of this complex indicate field-induced single molecule magnet (SMM) behavior. In contrast to the reaction with Co(II), the reaction of the macrocyclic ligand **H_3_L** with Ni(II) or Cu(II) salts results in the contraction of this [3 + 3] macrocycle and the formation of complexes of a smaller [2 + 2] macrocycle.

## Abstract 2

An enantiopure metal−organic cage **[Co_3_L_2_]** formed by the enantiomers of a large triphenolic macrocycle **L** selectively binds small guest molecules such as enantiomers of 2-aminobutane.

## Introduction

Macrocyclic complexes^1−8^ and metal–organic cages^9−21^ are classical supramolecular structures that attract increasing attention due to their elegant architecture and potential applications ranging from magnetic properties and catalysis to gas sorption and mimicking biological systems. Macrocycles, cryptands, cavitands, and cage-type compounds are of paramount importance in both organic and inorganic supramolecular chemistry due to their ability to bind guest molecules. This feature is related to the presence of a preorganized cavity within the host molecule. In particular, some large macrocycles have internal cavities that can accommodate several metal ions and/or additional guest molecules.^2,7,8^ Guest binding is also a characteristic feature of metal–organic cages (MOCs), also called metal–organic polyhedra, coordination cages, or metallacages. A judicious fine-tuning of the structure of such compounds may lead to size-selective, shape-selective, and/or functional group-selective binding of guest molecules.

**Scheme 1:**
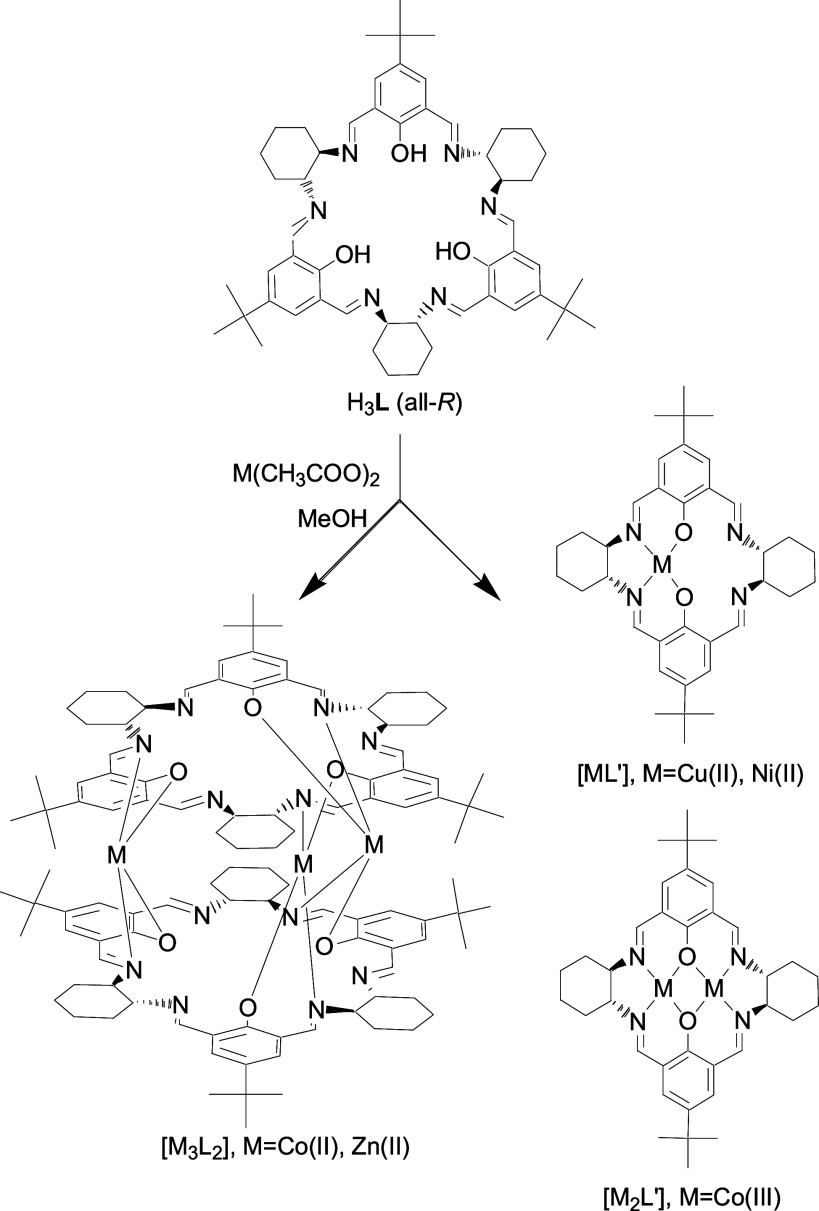
Formation of the Trinuclear Container-Like **[M**_**3**_**L**_**2**_**]** Complexes and the Rearrangement of Ligand **H**_**3**_**L** into Complexes of the Contracted Macrocycle **L′**

We have previously reported Zn(II) macrocyclic cage complexes **[Zn**_**3**_**L**_**2**_**]** based on large enantiopure [3 + 3] imine macrocycle L (Scheme 1), which is a 3 + 3 condensation product of chiral diamine (*trans*-1,2-diaminocyclohexane) and phenol dialdehyde (2,6-diformyl-4-*tert*-butylphenol).^22,23^ These cages are constructed from two macrocycles seamed by three Zn(II) ions and combine structural motifs of metallacages and macrocycles. The barrel-shaped molecule of **[Zn**_**3**_**L**_**2**_**]** forms a tight cage, which is able to bind small guest molecules. These compounds have exhibited substantial gas sorption properties and enantioselective binding of chiral alcohols.^23^ Recently, **[Zn_3_L_2_]** has been shown to exhibit D_2_/H_2_ selectivity and to be a very effective material for the separation of hydrogen isotopes via kinetic quantum sieving.^24^ Additionally, this macrocycle-based zinc(II) MOC has been shown to be a very effective agent for the detection and sequestration of perfluoroalkyl carboxylic acids, which are dangerous environmental pollutants.^25^ The same enantiopure **[Zn**_**3**_**L**_**2**_**]** complex has also been shown to be an excellent coated capillary column material for high-resolution chromatographic separation of a wide range of organic compounds, including racemates and positional isomers.^26^ In addition, various kinds of racemic compounds were separated with good enantioselectivity using **[Zn**_**3**_**L**_**2**_**]** as the stationary phase in capillary electrochromatography.^27,28^ It should be mentioned that metal–organic cages are typically built from several metal ions and several (usually relatively rigid) bi- or polydentate ligands, while examples of such cages based on macrocyclic units seamed by metal ions are relatively rare.^29−51^

Herein, we discuss the tendency of the macrocycle **H**_**3**_**L** to form analogous cage compounds with Cu(II), Ni(II), and Co(II) salts. We show that a stable metallacage is formed in the latter case (Scheme 1). We also demonstrate how the small diameter of the **[Co**_**3**_**L**_**2**_**]** cage and the tight binding of organic molecules result in the selective binding of guest molecules in solution, depending on the chirality and size of the guest. This cage, based on paramagnetic Co(II) ions, is an effective chiral shift reagent and causes unusual NMR differentiation of prochiral groups of the bound achiral guest molecules.

Chiral recognition is of paramount importance in nature and in many artificial chemical systems. Enantioselective binding and recognition of chiral organic molecules have been demonstrated for metal–organic cages,^52−80^ metal–organic frameworks (MOFs),^81−91^ and metallacycles,^92^ although this phenomenon is usually observed only in the solid state.

Cobalt(II) and cobalt(III) ions are used in the formation of many supramolecular structures. In particular, supramolecular complexes with triangular arrangements of these ions have been investigated in the context of magnetic interactions, cooperative reactivity of metal ions, as well as the formation of trinuclear metallacryptands or metallacages.^93−100^

## Results and Discussion

### Synthesis

The reaction of macrocycle **H**_**3**_**L** with transition metal salts strongly depends on the kind of metal ion. In the case of Zn(II) and Co(II) ions, trinuclear container-like complexes of the formula **[M**_**3**_**L**_**2**_**]** are formed in good yields in the reactions with metal acetates. Once formed, the trinuclear Co(II) complex **[Co**_**3**_**L**_**2**_**]** is quite stable toward oxidation or ligand dissociation. For instance, its NMR spectra of chloroform or benzene solutions do not change for at least 1 week (Figure S1). Only after a month of standing of the crude reaction mixture in methanol in the presence of triethylamine, the ligand partially decomposes, as indicated by the isolation of single crystals of **[Co**_**2**_**L′(AcO)**_**3**_**](AcO)·AcOH·3(H**_**2**_**O)** byproduct containing Co(III) ions bound side by side in a smaller [2 + 2] macrocycle **H**_**2**_**L′** (vide infra). Nevertheless, the ^1^H NMR spectra of the crude solid isolated from this reaction mixture indicate that **[Co**_**3**_**L**_**2**_**]** is still the main component. The stability toward ligand dissociation of the **[M**_**3**_**L**_**2**_**]** complexes is also manifested in metal exchange experiments. Thus, the ^1^H NMR spectra of the solution containing the mixture of **[Co**_**3**_**L**_**2**_**]** and [**Zn**_**3**_**L**_**2**_**]** complexes do not indicate the presence of significant amounts of the mixed species **[Co**_**2**_**ZnL**_**2**_**]** and [**CoZn**_**2**_**L**_**2**_**]**. On the other hand, such mixed Co(II)/Zn(II) complexes can be generated in the reaction between **H**_**3**_**L** and a mixture of Co(II) and Zn(II) acetates (Figure S2).

In contrast to the reaction with Co(II) acetate, the reactions of **H**_**3**_**L** with Cu(II) and Ni(II) acetates did not proceed smoothly. Although the ESI MS spectra of the crude reaction mixtures obtained directly after mixing the substrates indicate the presence of trinuclear **[Cu**_**3**_**L**_**2**_**]** and **[Ni**_**3**_**L**_**2**_**]** complexes, as confirmed by the observation of ions such as [Cu_3_(H_2_L)_2_]^2+^, [Ni_3_(H_2_L)_2_(H_2_O]^2+^, or [Ni_3_(H_2_L)_2_(H_2_O)_2_]^2+^, the attempts to isolate these trinuclear complexes resulted in the formation of complicated final mixtures. ESI MS data of these mixtures are in accord with the formation of mono- and dinuclear complexes of a smaller 2 + 2 macrocycle **H**_**2**_**L′** as the main products (Scheme 1). Moreover, the X-ray crystal structures of the crystals isolated from the mixtures obtained in the reactions of **H**_**3**_**L** and copper(II) perchlorate indicate mononuclear complexes of the protonated ligand **H**_**2**_**L′** (vide infra). Clearly, the Cu(II) and Ni(II) complexes of the [3 + 3] ligand **L**^**3–**^ are not thermodynamically stable, and instead, complexes of the smaller [2 + 2] macrocycle **H**_**2**_**L′** are formed. This process is an example of dynamic covalent chemistry^101^ and proceeds via hydrolytic cleavage and rearrangement of the imine bonds of the [3 + 3] macrocycle **H**_**3**_**L**. An analogous rearrangement was previously observed for the reactions of similar [3 + 3] macrocycles.^102,103^ The difference in the behavior of the Co(II) and Zn(II) acetates in the reaction with **H**_**3**_**L**, in comparison with the behavior of Cu(II) and Ni(II) acetates, reflects the higher tendency of the former two ions to form tetrahedral complexes. Notably, the high-spin *d*^7^ Co(II) ions exhibit the highest relative LFSE (ligand field stabilization energy) among the first-row transition metal M^2+^ ions for complexes of tetrahedral geometry.

### X-Ray Crystal Structures

**Figure 1:**
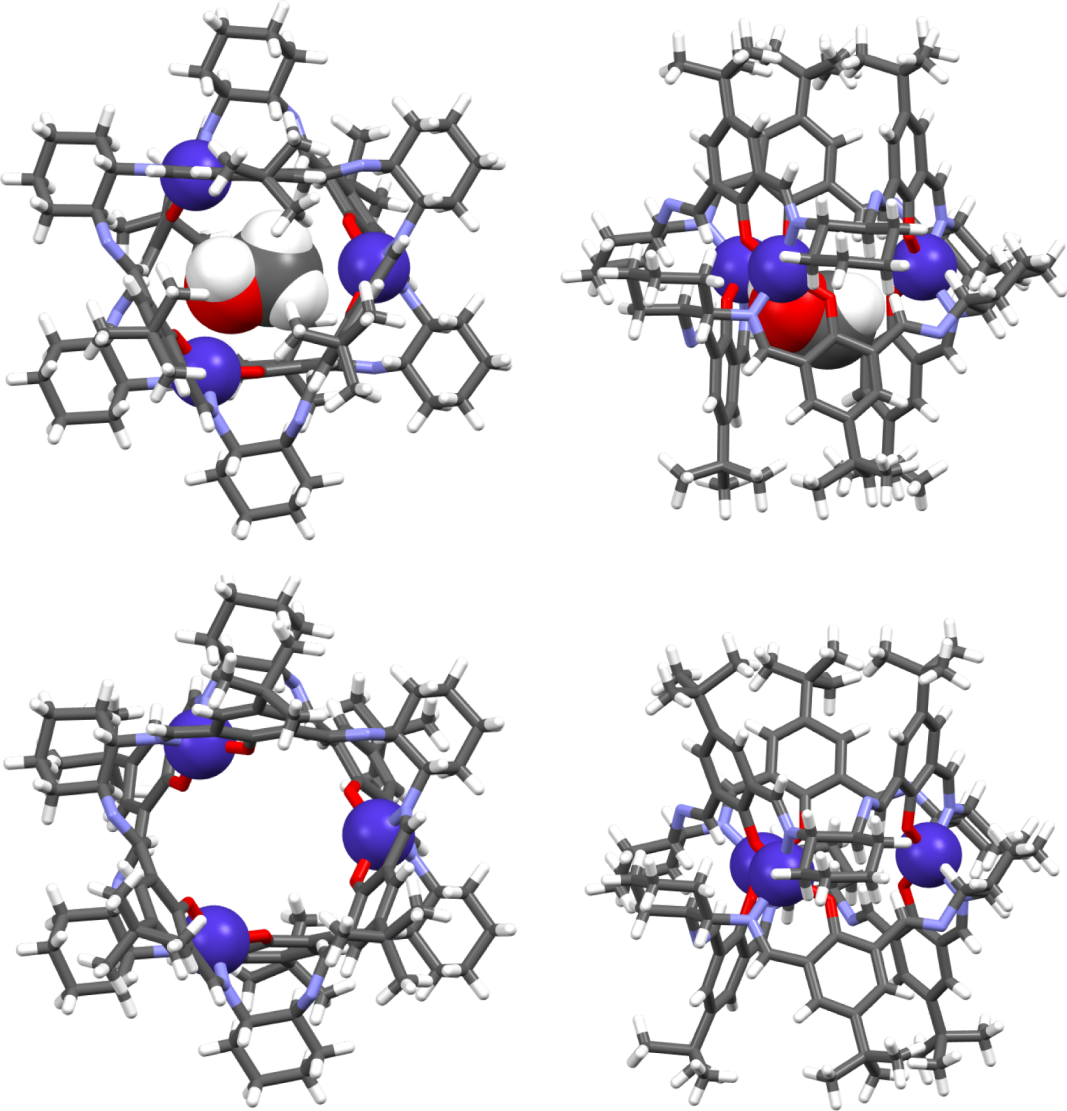
Upper panel: top and side views of the **[Co**_**3**_**L***^**S**^*_**2**_**]** complex crystallized from methanol. The guest solvent molecule and Zn(II) ions are in a space-filling representation. Lower panel: top and side views of the **[Co**_**3**_**L***^**S**^*_**2**_**]** complex crystallized from chloroform. The solvent (e.g., water) molecules inside the cavity are not shown because it was not possible to correctly model these disordered molecules.

The X-ray crystal structure of the complex formed in methanol shows the **[Co**_**3**_**L***^**R**^*_**2**_**]** complex, where three Co(II) ions link two macrocyclic ligands. The macrocycle **L** adopts a tubular conformation with almost parallel phenyl rings, thus forming a barrel-shaped complex molecule with a methanol guest inside (Figures 1 and S3–S5). The N_2_O_2_ coordination sphere of each Co(II) ion corresponds to a highly distorted tetrahedron, and the bond lengths are in accord with the high-spin Co(II) ion. The distortion of the coordination sphere is imposed by the relatively rigid macrocyclic ligand. The deviation of the metal center from the ideal tetrahedral geometry may be described by the geometry index^104^ τ_4_:
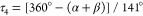
where α and β are the two largest angles in the coordination sphere, or alternatively by the index^105^ τ’_4_:
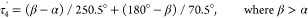


The values of both indexes are equal to 1 for a perfect tetrahedral geometry and zero for a perfect square planar geometry. In the discussed case, the values of τ_4_ = 0.71 and τ’_4_ = 0.69 indicate that the tetrahedral geometry around Co(II) ions is considerably distorted toward a square-planar one.

The structure of the trinuclear Co(II) complex **[Co**_**3**_**L***^**R**^*_**2**_**]** grown from methanol is isomorphic with the analogous trinuclear Zn(II) complex **[Zn**_**3**_**L***^**R**^*_**2**_**]** grown from the same solvent.^24^ However, it is not isomorphic with another form of **[Zn**_**3**_**L***^**R**^*_**2**_**]** crystals also grown from methanol,^22^ although the overall geometries of the three complex molecules are very similar. In the crystal of the latter Zn(II) crystalline form, the cavities of individual barrels are on top of each other, and a channel-type structure is formed. The molecular structure of the complex **[Co**_**3**_**L***^**R**^*_**2**_**]** observed in the crystalline form obtained from benzene (Figures S6–S8) is very similar to that of the form obtained in methanol. On the other hand, the barrel structure is more open for the crystalline form obtained from chloroform, with the macrocyclic ligands assuming a cone conformation (Figures 1 and S9–S11). These variations indicate a greater influence of the solvent and crystallization conditions on the crystal packing and exact cage conformation compared with the change from Zn(II) to Co(II) in **[M**_**3**_**L**_**2**_**]**.

**Figure 2:**
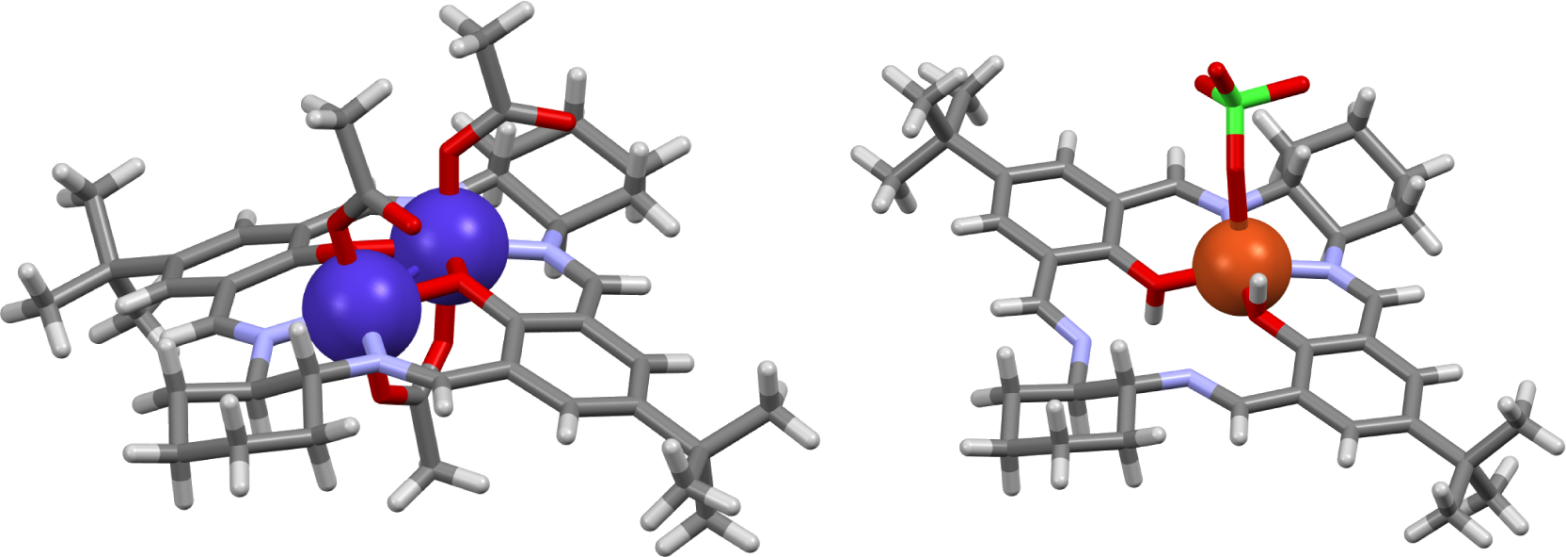
Left: structure of the **[Co**_**2**_**L′(AcO)**_**3**_**]**^**+**^ cationic complex. Right: structure of the **[Cu(H**_**2**_**L′)(ClO**_**4**_**)]**^**+**^ cationic complex.

As mentioned in the previous section, in some reactions, the complexes of the [3 + 3] macrocycle **H**_**3**_**L** are converted into complexes of the contracted macrocycle [2 + 2], **H**_**2**_**L′**. The X-ray crystal structure of the **[Co**_**2**_**L′(AcO)**_**3**_**](AcO)·AcOH·3H**_**2**_**O** reveals the cationic Co(III) complex where the [2 + 2] macrocyclic ligand is somewhat bent (Figures 2, S12, and S13). The two Co(III) ions are positioned in the two N_2_O_2_ compartments of the macrocycle and are coordinated by terminal acetate anions. An additional acetate anion bridges the two metal ions on the concave side of the macrocycle. The structures of the two Cu(II) complexes of the macrocycle **H**_**2**_**L′** indicate the presence of mononuclear forms with the metal ion bound in one of the two roughly square N_2_O_2_ compartments. The conformation of the [2 + 2] ligand in the mononuclear complexes **[Cu(H**_**2**_**L′)(ClO**_**4**_**)](ClO**_**4**_**)** (Figures 2, S14, and S15) and **[Cu(H**_**2**_**L′)(CH**_**3**_**OH)](ClO**_**4**_**)**_**2**_**·CH**_**3**_**OH** (Figures S16–S18) is more flat and resembles that of the dinuclear Zn(II) complex.^22^ In both complexes, the Cu(II) ion interacts weakly with an additional axial ligand – perchlorate anion or methanol, respectively.

### Spectroscopic and Magnetic Properties

**Figure 3:**
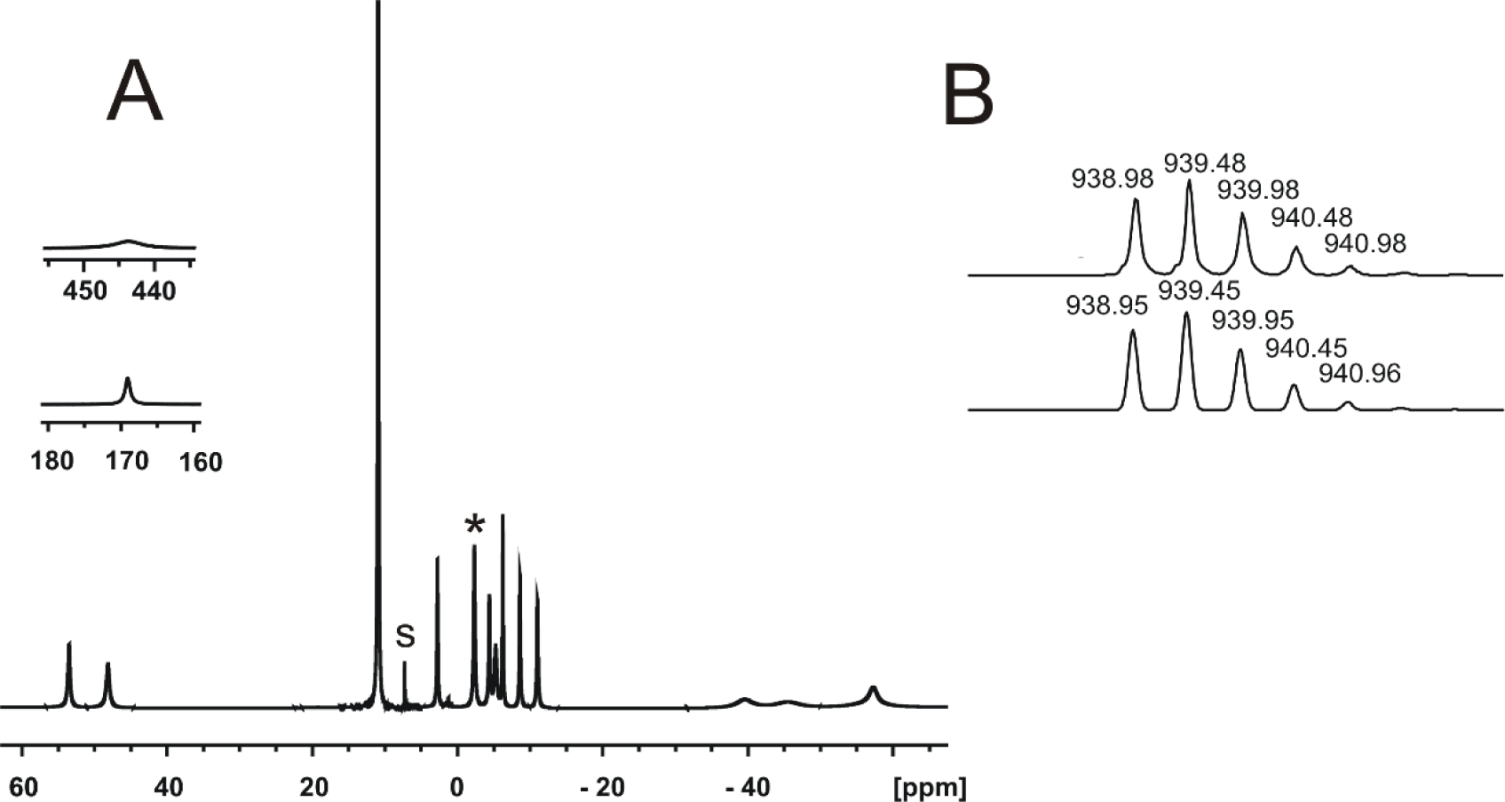
A) ^1^H NMR spectrum of the **[Co**_**3**_**L**_**2**_**]** complex (CDCl_3_, 298 K, 500 MHz, 1024 scans), s – solvent and * signal of bound water. B) Fragment of the experimental (top) and calculated (bottom) ESI MS spectra of **[Co**_**3**_**L**_**2**_**]**.

The formation of the enantiomeric trinuclear cobalt(II) complexes **[Co**_**3**_**L**_**2**_**]** is confirmed by the ESI mass spectrum, showing signals with a characteristic isotope pattern (Figure 3) at *m*/*z* 626.7 {**[Co**_**3**_**L**_**2**_**]**+3H}^3+^ ([C_108_H_141_Co_3_N_12_O_6_]^3+^), 939.5 {**[Co**_**3**_**L**_**2**_**]**+2H}^2+^ ([C_108_H_140_Co_3_N_12_O_6_]^2+^) and 1877.9 {**[Co**_**3**_**L**_**2**_**]**+H}^+^ ([C_108_H_139_Co_3_N_12_O_6_]^+^) corresponding to the intact cage, which is triply, doubly, or singly protonated at the nitrogen atoms that do not take part in metal ion coordination.

The ^1^H NMR spectrum of **[Co**_**3**_**L**_**2**_**]** consists of 14 strongly broadened and paramagnetically shifted signals ranging from −57 ppm to 443 ppm (Figure 3). This spectral pattern is in accord with the effective *C*_*3*_ symmetry of this complex.

**Figure 4:**
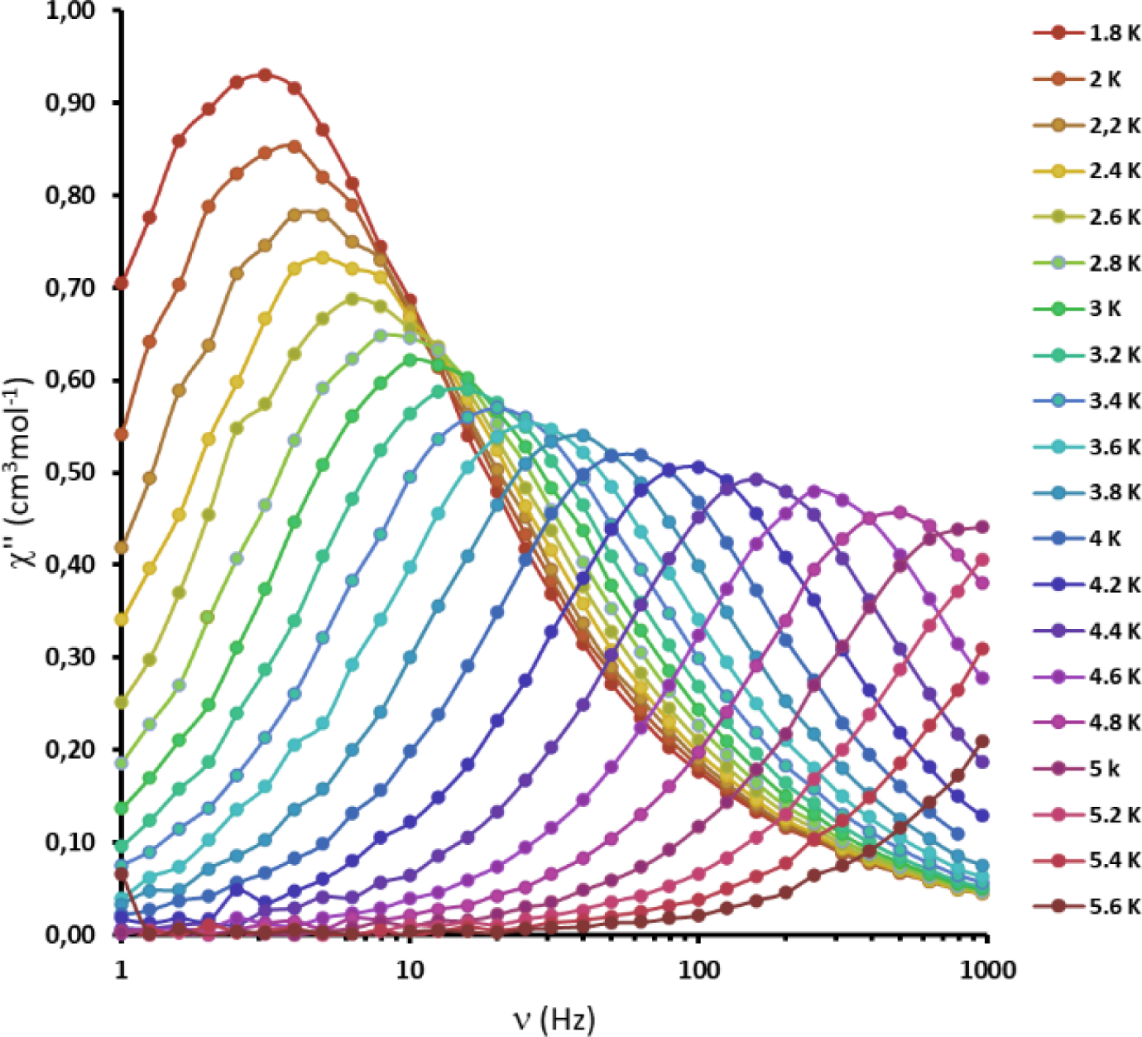
Frequency dependence of the imaginary χ” components of the *ac* susceptibility of **[Co**_**3**_**L**_**2**_**]** at different *ac* frequencies between 1 and 960 Hz and different temperatures (3 Oe *ac* field and 1500 Oe *dc*-field, the solid lines are for the guidance only).

The presence of three high-spin Co(II) ions is additionally confirmed by magnetic measurements, which indicate the value of the χ_M_T product of molar magnetic susceptibility and temperature equal to 7.06 cm^3^ mol^–1^ K at 300 K. This value corresponds to a magnetic moment equal to 4.34 μ_B_ per metal ion, in accord with the values expected for high-spin Co(II) ions with orbital contribution. Variable temperature magnetic susceptibility data for **[Co**_**3**_**L**_**2**_**]** were obtained in the temperature range of 1.8–300 K. The value of the χ_M_T product slightly changes on cooling to ca. 50 K (Figure S19). Below this temperature, χ_M_T starts to decrease substantially to reach the value of 3.83 cm^3^ mol^–1^ K at 1.8 K. This decrease is typical for Co(II) complexes with magnetic anisotropy, although it may indicate additional weak antiferromagnetic interactions. Magnetic anisotropy is also in accord with the field dependencies of the magnetization measured at 2, 3, and 5 K and the observation of the magnetization *M* versus field *H* isotherms that are not superimposable (Figure S20). Due to the magnetic anisotropy of high-spin Co(II) centers, some complexes of these ions exhibit slow magnetic relaxation and attract attention as single-molecule magnets (SMMs).^106−113^ In order to probe the dynamic magnetic behavior of **[Co**_**3**_**L**_**2**_**]**, alternating current (*ac*) susceptibility data were recorded. Although no appreciable out-of-phase *ac* susceptibility χ″ signals were observed under a zero static (*dc*) magnetic field, due to the quantum tunneling of magnetization (QTM), slow magnetic relaxation was observed when an additional *dc* field was applied to suppress QTM. The temperature and frequency-dependent maxima of the out-of-phase χ″, as well as Cole–Cole plots of χ″ vs in-phase *ac* susceptibility χ′, are in accord with SMM behavior under an applied *dc* field of 1500 Oe (Figures 4 and S21–S23). The data were fitted using a generalized Debye model, and the distribution of the relaxation time was determined on the basis of the Cole–Cole plot using CC-Fit2 software.^114^ The obtained α Cole–Cole parameter values range from 0.03 to 0.24 at 1.8–5.0 K, in accord with a rather narrow distribution of relaxation times. The plot of the natural logarithm of the relaxation time ln(τ) vs inverse temperature (Figure S24) indicates the dominant Orbach and Raman relaxation mechanisms with an energy barrier *U*_eff_ of 46.3 cm^–1^ (66.7 K) and a pre-exponential relaxation time τ_0_ of 3.5 × 10^–10^ s.

### Enantioselective and Size-Selective Guest Binding

**Figure 5:**
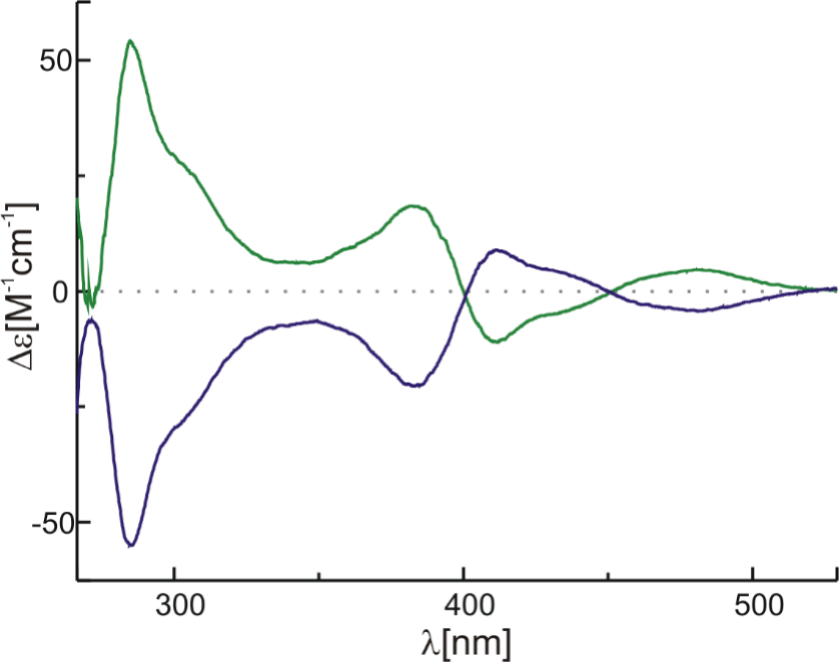
CD spectra of [**Co**_**3**_**L***^**R**^*_**2**_**]** (blue) and [**Co**_**3**_**L***^**S**^*_**2**_**]** (green) complexes in CHCl_3_.

Because the enantiopure forms of the macrocyclic ligand **H_3_L** were used for the syntheses, the obtained cage complexes were also enantiopure. The chiral nature of the **[Co**_**3**_**L**_**2**_**]** complex is reflected in the mirror CD spectra of the **[Co**_**3**_**L***^**R**^*_**2**_**]** and **[Co**_**3**_**L**^***S***^_**2**_**]** complexes with the macrocyclic ligand of all-*R* and all-*S* configuration, respectively (Figure 5). The enantiopure nature of [**Co**_**3**_**L**_**2**_**]** prompted us to study the interaction of these trinuclear Co(II) complexes with chiral guests and test their efficiency in enantiodifferentiation^115−125^ of ^1^H NMR signals of chiral molecules.

**Figure 6:**
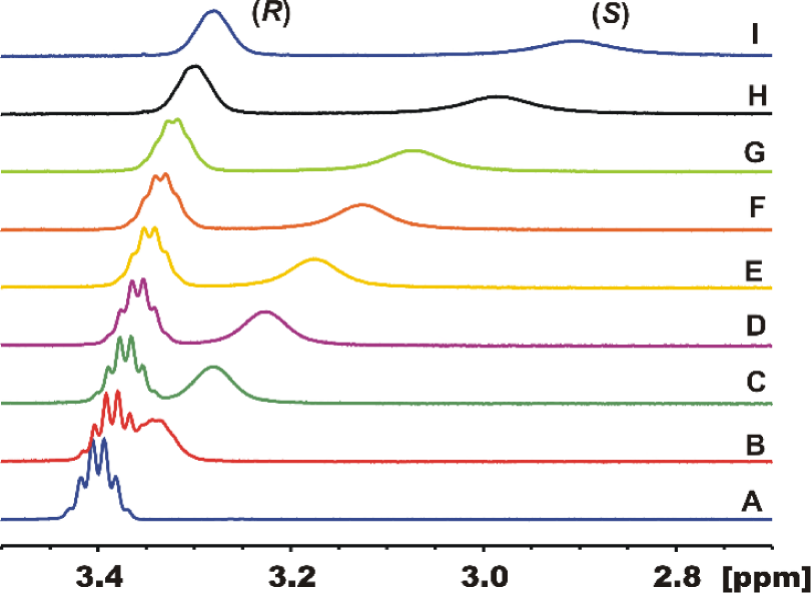
Splitting of the CH_3_CH(OH)CH_2_CH_3_^1^H NMR signal of the racemic 2-butanol (298 K, 500 MHz, 0.04 M solution in C_6_D_6_) after addition of increasing amounts of the **[Co**_**3**_**L***^**R**^*_**2**_**]** complex. Traces A–I correspond to 0, 0.0024, 0.0048, 0.0072, 0.0096, 0.012, 0.014, 0.019, and 0.024 equiv of the added Co(II) cage complex, respectively.

We have previously observed enantiodiscrimination of ^1^H NMR signals of chiral racemic guests bound by the analogous diamagnetic **[Zn**_**3**_**L**_**2**_**]** cage.^23^ In the case of the high-spin Co(II) derivative, one can expect that such discrimination will be much more pronounced due to the paramagnetic effects, in particular, the dipolar contribution to the chemical shifts of guest protons. Indeed, the interaction of various chiral and achiral organic compounds with the **[Co**_**3**_**L**_**2**_**]** cage resulted in a substantial shift of the ^1^H NMR signals of small guest molecules (Figures 6, S25, S26, S46, and S49–S53). For instance, the titration of racemic 2-butanol with a solution of **[Co**_**3**_**L***^**R**^*_**2**_**]** in C_6_D_6_ or CDCl_3_ solvent resulted in the gradual shift and splitting of the ^1^H NMR signals (Figures 6 and S25). This splitting effect is due to spectroscopic enantiodiscrimination of 2-butanol enantiomers, as confirmed by analogous ^1^H NMR titrations of enantiopure (*R*)-2-butanol and (*S*)-2-butanol (Figure S26). Notably, the effect of the paramagnetic host **[Co**_**3**_**L***^**R**^*_**2**_**]** on the NMR signals of the guest molecule is larger for the (*S*) enantiomer. The Δδ values are almost 5 times larger for the (*S*)-2-butanol in comparison with (*R*)-2-butanol (Figure S27) that may indicate substantial enantioselectivity in the binding of 2-butanol enantiomers by this Co(II) cage. This selectivity results from the relatively small cavity of **[Co**_**3**_**L**_**2**_**]** which leads to a tight fit of the guest molecule. The preferred binding of (*S*)-2-butanol is also reflected in larger NMR line broadening effects for this enantiomer due to the interaction with the paramagnetic Co(II) centers. Similar spectroscopic enantiodifferentiation was also observed for other chiral alcohols. It should be noted that the observed spectroscopic effects correspond to equilibria in solution and guest binding on a molecular level, as opposed to the enantioselective binding in the solid state. The latter case was observed previously for 2-butanol and similar compounds in a number of crystalline systems^64,74,81−91^ and thin films.^55^

Although the paramagnetic effects enhance NMR spectroscopic enantiodifferentiation due to the very large difference between the chemical shifts of the free diamagnetic guest and that of the bound guest coordinated to paramagnetic Co(II), the accompanying line broadening does not allow following the signals of guests in the case of larger paramagnetic host concentrations. Thus, the plots in Figure S27 correspond to the initial, practically linear, parts of binding isotherms (up to 0.024 equiv of added Co(II) cage host) and cannot be extended much further because of severe line broadening followed by the final disappearance of 2-butanol signals. This effect, together with the relatively small binding constants, does not allow for quantitative determination of binding constants based on the variations of the guest chemical shifts with different host concentrations. It should be mentioned that the difference between the chemical shifts of the diamagnetic guest and the shifts of the guest coordinated to Co(II) in the cage will be large (on the order of tens or hundreds of ppm) because the fully bound guest will be part of a paramagnetic complex. In such cases, the line broadening is not a simple combination of exchange and paramagnetic contributions. On the other hand, the binding of the guest does not strongly influence the structure of the Co(II) host, and the differences between the chemical shifts of free and bound paramagnetic forms of **[Co**_**3**_**L**_**2**_**]** are smaller. As a result, for some resonances, averaged signals corresponding to the fast NMR exchange regime can be observed. Therefore, we have analyzed the variations of the chemical shifts of the **[Co**_**3**_**L***^**R**^*_**2**_**]** host at different concentrations of 2-butanol enantiomers (Figures S28 and S29). The obtained binding isotherms (Figure S30) allowed the estimation of the apparent 1:1 binding constants. The obtained value of the binding constant *K* for the formation of (*S*)-2-butanol⊂**[Co**_**3**_**L***^**R**^*_**2**_**]** is equal to 9.26 M^–1^ (1.7% error), and that for the (*R*)-2-butanol⊂**[Co**_**3**_**L***^**R**^*_**2**_**]** is equal to 6.07 M^–1^ (2.1% error). This confirms the preferred binding of the 2-butanol guest of opposite chirality to that of the host cage, although the difference is moderate. The binding constant value for the achiral isomer of 1-butanol, equal to 11.36 M^–1^ (2.9% error), is higher than those for both enantiomers of 2-butanol (Figure S31).

For comparison, we have reinvestigated the interaction of 2-butanol with the isostructural diamagnetic Zn(II) cage. The **[Zn**_**3**_**L***^**R**^*_**2**_**]** and **[Zn**_**3**_**L***^**S**^*_**2**_**]** enantiomers of the host were titrated with (*R*)-2-butanol (Figure S32). It should be noted that the binding constant for the guest/host pair (*R*)-2-butanol⊂**[Zn**_**3**_**L***^**S**^*_**2**_**]** is equivalent to that of the enantiomeric pair (*S*)-2-butanol⊂**[Zn**_**3**_**L***^**R**^*_**2**_**]** and different from the binding constant for the enantiomeric pairs (*R*)-2-butanol⊂**[Zn**_**3**_**L***^**R**^*_**2**_**]** and (*S*)-2-butanol⊂**[Zn**_**3**_**L***^**S**^*_**2**_**]**. The apparent 1:1 binding constant *K* for the formation of (*R*)-2-butanol⊂**[Zn**_**3**_**L***^**S**^*_**2**_**]** is equal to 1.90 M^–1^ (1.1% error), and that for the (*R*)-2-butanol⊂**[Zn**_**3**_**L***^**R**^*_**2**_**]** is equal to 1.57 M^–1^ (0.8% error), based on the obtained binding isotherms (Figure S33). Similar to the Co(II) system, binding of the 2-butanol guest of opposite chirality to that of the host cage is preferred, although both binding constants are significantly lower, indicating a higher tendency of Co(II) ions to increase the coordination number in this kind of host/guest system. The preference for the formation of the host/guest pair of opposite chirality observed in solution is in accord with the selective binding of only one of the enantiomers of 2-pentanol or 2-aminobutane within the **[Co**_**3**_**L***^**R**^*_**2**_**]** host observed in solid-state crystal structures (vide infra). It is also in accord with our previously reported X-ray crystal structure of single crystals obtained in the reaction of **[Zn**_**3**_**L***^**R**^*_**2**_**]** with racemic 2-butanol. This structure showed that **[Zn**_**3**_**L***^**R**^*_**2**_**]** selectively binds the enantiomer of 2-butanol of opposite chirality in the solid state.^23^ The stronger binding of the 2-butanol molecule of opposite chirality to that of the Zn(II) host is also consistent with the application of **[Zn**_**3**_**L***^**R**^*_**2**_**]** as a stationary phase for gas chromatography and the observation that the retention time of the (*S*)-enantiomer of 2-butanol was longer than that of the (*R*)-enantiomer.^26^

We also investigated the influence of **[Co**_**3**_**L**_**2**_**]** on the chemical shifts of chiral amines, such as 2-aminobutane. In this case, addition of the host resulted in severe broadening and finally the disappearance of the guest ^1^H NMR signals. Nevertheless, the binding of chiral amines is also enantioselective, as indicated by the differences in the extent of line-broadening effects for the (*S*)-2-aminobutane⊂**[Co**_**3**_**L***^**R**^*_**2**_**]** and (*S*)-2-aminobutane⊂**[Co**_**3**_**L***^**R**^*_**2**_**]** diastereomeric pairs. After addition of small amounts of **[Co**_**3**_**L***^**R**^*_**2**_**]** to the (*S*)-2-aminobutane solution in deuterated benzene, the signals of amine broaden in ^1^H NMR spectra measured at 600 MHz but do not shift, while the signal of the added Co(II) complex is broadened beyond detection (Figure S34). In contrast, upon the addition of a small amount of **[Co**_**3**_**L***^**S**^*_**2**_**]** enantiomer, the (*S*)-2-aminobutane guest signals shift and broaden much more extensively. Also, in contrast with the **[Co**_**3**_**L***^**R**^*_**2**_**]** case, the signal of *tert*-butyl groups of the added **[Co**_**3**_**L***^**S**^*_**2**_**]** host is observable and is only slightly shifted and broadened compared to that of the pure host (Figure S34). These differences are related to the different rates of binding to the two host enantiomers, taking into account the larger difference between the chemical shifts of the free and complexed amine compared to the difference between the chemical shifts of the free and bound **[Co**_**3**_**L***^**R**^*_**2**_**]** host forms. The observed effects on the guest signals can be explained by the fact that the exchange rate is between the slow and intermediate exchange regimes at 600 MHz for the (*S*)-2-aminobutane⊂**[Co**_**3**_**L***^**R**^*_**2**_**]** pair, whereas the exchange rate is closer to the fast exchange regime for the (*S*)-2-aminobutane⊂[**Co**_**3**_**L***^**S**^*_**2**_**]** pair. Additionally, at higher host concentrations, the paramagnetic range of ^1^H NMR spectra indicated shifting and broadening of **[Co**_**3**_**L**_**2**_**]** host signals, depending on the chirality of the host/guest system (Figures S35 and S36). In the case of the (*S*)-2-aminobutane⊂**[Co**_**3**_**L***^**R**^*_**2**_**]** pair, additional poorly defined ^1^H NMR signals were observed, which may correspond to the bound amine (Figure S35). The observed ^1^H NMR effects indicate much stronger binding of amines in comparison to that of alcohols. In the latter case, weaker binding corresponds to a fast exchange regime in the ^1^H NMR spectra. In the case of amines, the stronger binding results in rates closer to the intermediate or slow exchange regime, which renders very broad NMR signals.

^1^H NMR spectra of the **[Co**_**3**_**L**_**2**_**]** host at various concentrations of the (*S*)-2-aminobutane guest were also measured at 300 MHz in order to achieve a faster NMR exchange regime. Even at this frequency, the fast exchange regime could not be achieved for many host signals, and a marked difference between the signal widths of the (*S*)-2-aminobutane⊂**[Co**_**3**_**L***^**S**^*_**2**_**]** and the (*S*)-2-aminobutane⊂**[Co**_**3**_**L***^**R**^*_**2**_**]** pairs was still observed (Figures S37 and S38). The fastest exchange regime should be obtained for the signal that is least affected by the addition of amine, i.e., the signal of host *t*-butyl protons (Figure S38), which are located farthest from the paramagnetic Co(II) ions. Based on the obtained binding isotherms (Figure S39), the apparent 1:1 binding constant for the (*S*)-2-aminobutane⊂**[Co**_**3**_**L***^**S**^*_**2**_**]** pair was estimated to be 57.5 M^–1^ (1.9% error). The binding isotherm of the (*S*)-2-aminobutane⊂**[Co**_**3**_**L***^**R**^*_**2**_**]** pair clearly indicates a much stronger guest complexation, although, in this case, only a rough estimation of the apparent 1:1 binding constant *K* of 1500 M^–1^ (35% error) was possible. Both binding constants are significantly higher compared to those of the corresponding 2-butanol system, in accordance with the stronger coordination abilities of amines compared to alcohols.

In the case of *t*-butyl ^1^H NMR signals measured at 300 MHz, significant broadening can still be observed upon the addition of the *S*-enantiomer of the amine guest. This broadening is not mainly caused by chemical exchange between the free and bound guest, because after adding more than 2.47 equiv of (*S*)-2-aminobutane, the signals practically do not shift anymore (Figure S38) and almost complete saturation of the host is obtained. Thus, the significantly broadened signals of the *t*-butyl groups of the macrocycle in (*S*)-2-aminobutane⊂**[Co**_**3**_**L***^**R**^*_**2**_**]** are an intrinsic feature of this complex. This broadening is rather caused by a chemical exchange process corresponding to the rotation of the guest in the cage and switching of the coordination site between the three Co(II) ions of **[Co**_**3**_**L***^**R**^*_**2**_**]**. Such rotation results in averaging of the macrocyclic ligand signals and the effective average *C*_3_ symmetry of the host–guest complex, which would otherwise have lower symmetry. Most likely, in the case of the (*S*)-2-aminobutane⊂**[Co**_**3**_**L***^**R**^*_**2**_**]** pair, due to the strongest guest binding, this rotation is slower and the averaging is incomplete compared to other pairs.

For comparison, we have estimated the apparent binding constants for (*S*)-2-aminobutane interactions with the isostructural diamagnetic Zn(II) host by using ^1^H NMR titration experiments (Figure S40). The deuterated benzene solutions of **[Zn**_**3**_**L***^**R**^*_**2**_**]** or **[Zn**_**3**_**L***^**S**^*_**2**_**]** were titrated with (*S*)-2-aminobutane and the obtained binding isotherms (Figure S33) were used to estimate the apparent 1:1 binding constants. The *K* = 184.0 M^–1^ (3.64% error) value for the (*S*)-2-aminobutane⊂**[Zn**_**3**_**L***^**R**^*_**2**_**]** pair turned out to be much higher in comparison with the *K* = 6.35 M^–1^ (1.54% error) value for the (*S*)-2-aminobutane⊂**[Zn**_**3**_**L***^**S**^*_**2**_**]** pair, again confirming a strong preference for binding of the guest enantiomer that is of opposite chirality to that of the host.

**Figure 7:**
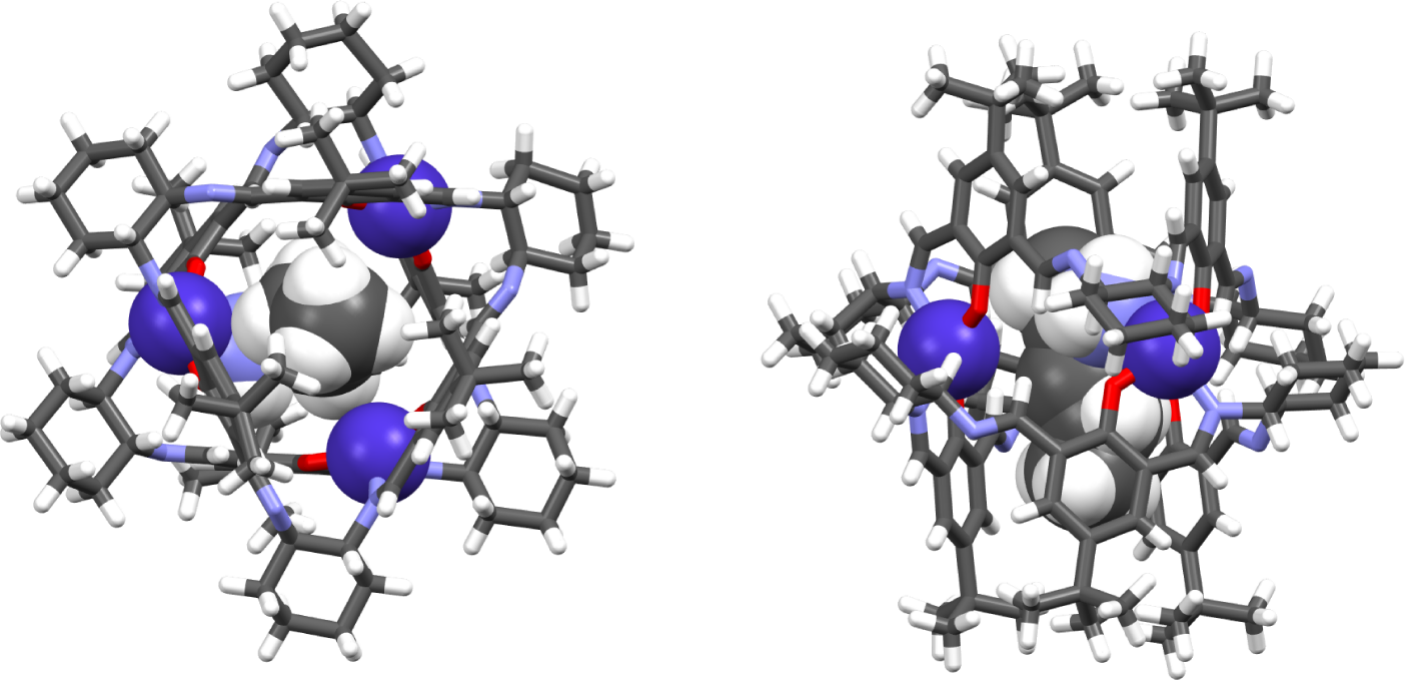
Top and side view of the **[Co**_**3**_**L***^**R**^*_**2**_**(***S***-CH**_**3**_**CH(NH**_**2**_**)CH**_**2**_**CH**_**3**_**)]** cage with the coordinated (*S)*-2-aminobutane molecule and Co(II) ions in a space-filling representation.

The selective binding of enantiomers of 2-aminobutane by the Co(II) cage is clearly observed in the solid state. The X-ray crystal structure of a derivative **[Co**_**3**_**L***^**R**^*_**2**_**(***S***-CH**_**3**_**CH(NH**_**2**_**)CH**_**2**_**CH**_**3**_**)]·2C**_**6**_**H**_**6**_**·H**_**2**_**O** that crystallized from the solution containing **[Co**_**3**_**L***^**R**^*_**2**_**]** and racemic 2-aminobutane indicates that the *S*-enantiomer is selectively bound within the trinuclear cage molecule (Figures 7 and S41–S43). The molecular structure of the resulting **[Co**_**3**_**L***^**R**^*_**2**_**(***S***-CH**_**3**_**CH(NH**_**2**_**)CH**_**2**_**CH**_**3**_**)]** complex shows that the disordered amine molecule tightly fits within a barrel-shaped trinuclear complex. The amine nitrogen atom is coordinated to one of the Co(II) ions, although the bond length is untypically large −2.31 Å. In contrast, the Co(II)–N bonds connecting the metal ion with the imine nitrogen atoms of the macrocyclic ligand, equal to 2.09 and 2.11 Å, are more typical for high-spin Co(II). The coordination sphere around this ion is also untypical and is highly distorted. The irregular geometry is reflected by the value of the index of trigonality^126^ τ equal to 0.23 (τ = (β – α)/60, where α and β are the two largest angles in the coordination sphere around the penta-coordinate Co(II)). The above structural parameters indicate that the rigid structure of the cage does not allow the formation of a typical pentacoordinate Co(II) and results in a rather weak coordination bond with the guest molecule. Thus, the binding of this kind of guest is based on the combination of weak interactions and coordination with the metal center. The latter factor should be more important for the amine guests, which are better ligands in comparison with alcohols, ketones, or esters. This stronger binding of the amine guest explains the absence of fast chemical exchange on the NMR time scale between bound and free guest molecules.

The binding of a single enantiomer of a guest within the enantiopure cage **[Co**_**3**_**L**_**2**_**]** observed in the solid state is in accord with the enantioselective binding in solution but does not guarantee effective separation of enantiomers in the solid state. This is because, in the crystal structure, amine or alcohol molecules may be present not only within the cage but also fill the voids in between the complex molecules. For instance, the crystal structure of the derivative that was crystallized from the benzene solution containing **[Co**_**3**_**L***^**R**^*_**2**_**]** and racemic 2-pentanol indicates that, apart from the guest molecule of *S*-chirality bound in the cage, additional 2-pentanol molecules are present in between the cage molecules (Figures S44 and S45). Similarly, the X-ray structures of analogous zinc(II) cage **[Zn**_**3**_**L**_**2**_**]** derivatives obtained in the presence of perfluoroalkyl carboxylic acids reveal that these achiral guest molecules are not bound inside the cage cavity.^25^

**Figure 8:**
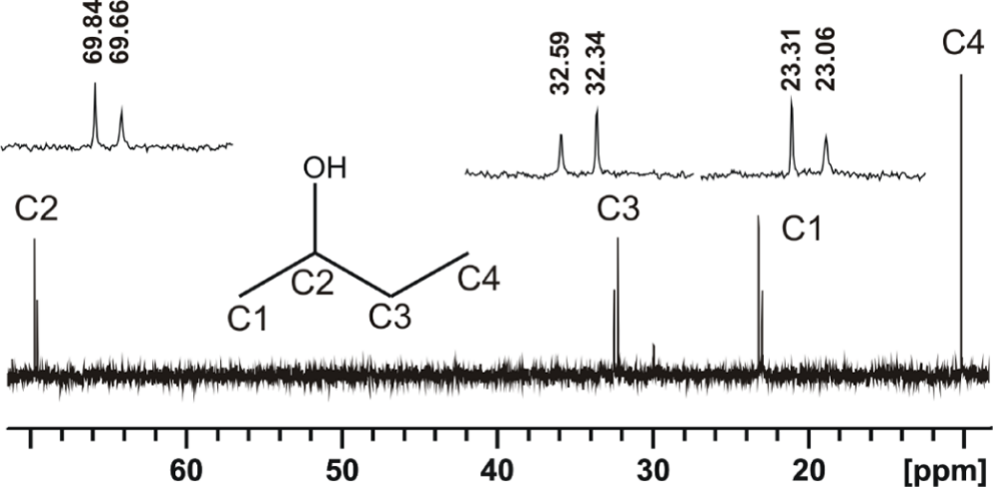
Splitting of the ^13^C-{^1^H} NMR signals of racemic 2-butanol (298 K, 125 MHz, 7168 scans, 0.036 M solution in CDCl_3_) after addition of 0.028 equiv of **[Co**_**3**_**L***^**S**^*_**2**_**]**.

The paramagnetic **[Co**_**3**_**L**_**2**_**]** cage has a much larger influence on NMR signals of guest molecules in comparison with the analogous diamagnetic **[Zn**_**3**_**L**_**2**_**]** complex. For instance, the Co(II) complex exerts clear effects on signals of 1-phenylethanol or 1-propanol, while the Zn(II) counterpart used at the same concentrations has practically no effect (Figures S46 and S47). The application of the paramagnetic cobalt(II) cage also results in the resolution of ^13^C NMR signals of the racemic 2-butanol into enantiomer signals of equal intensity (Figures 8and S48), whereas the application of the Zn(II) cage complex does not result in such an effect. The **[Co**_**3**_**L**_**2**_**]** complex can lead to a baseline resolution of the ^1^H NMR signals of enantiomers when used at a very low host:guest ratio, e.g., as low as 1:200. The other advantage of the Co((II) derivative is that there is very little overlap of the paramagnetic NMR signals of the host molecule with the signals of the guest.

**Figure 9:**
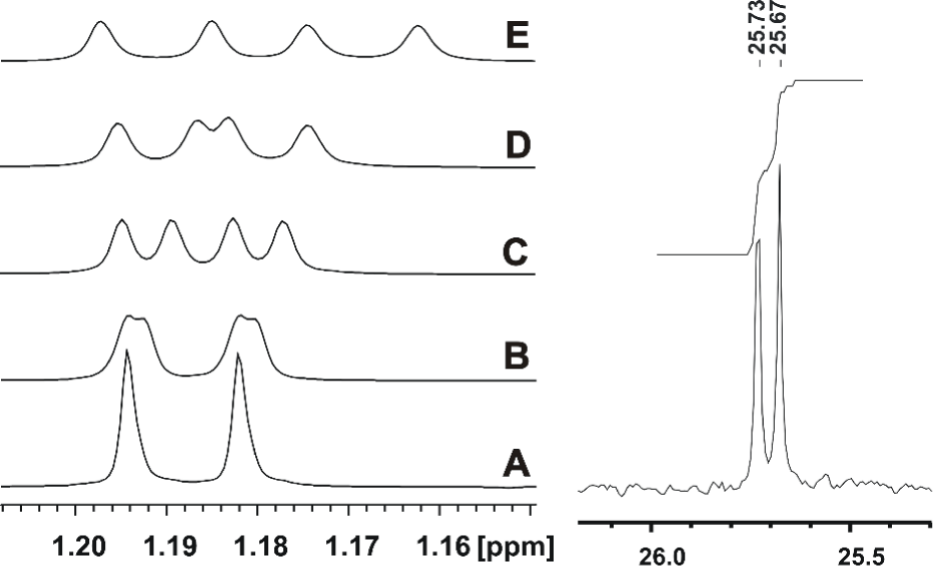
Left: splitting of the ^1^H NMR signal (doublet originating from vicinal coupling with a CH group) of the methyl group of 2-propanol (0.04 M solution in CDCl_3_, 298 K, 500 MHz, 16 scans) after addition of increasing amounts of the **[Co**_**3**_**L***^**R**^*_**2**_**]** complex. Traces A–E correspond to 0, 0.004, 0.008, 0.012, and 0.031 equiv of the added Co(II) complex, respectively. Traces B and C correspond to discrimination of pro-*R* and pro-*S* methyl groups bound within the chiral host. Right: splitting of the ^13^C-{^1^H} NMR signal of the methyl group of 2-propanol after addition of 0.031 equiv of **[Co**_**3**_**L***^**R**^*_**2**_**]** 0.04 M solution in (CDCl_3_, 298 K, 125 MHz, 7168 scans).

The enantiopure nature of the paramagnetic **[Co**_**3**_**L**_**2**_**]** cage results in unusual NMR effects for small guest molecules that are prochiral. The enantiotopic protons of such prochiral molecules are no longer NMR equivalent when the host is bound in **[Co**_**3**_**L***^**R**^*_**2**_**]**. Thus, doubling of the methylene ^1^H NMR signals of ethanol and doubling of the methyl signals of 2-propanol as well as of DMSO is observed in the ^1^H NMR spectra (Figures 9, S49, and S50). The pro-*R* and pro-*S* protons of the CH_2_ group in ethanol (whose substitution would lead to enantiomers) are equivalent in ^1^H NMR spectra of ethanol alone, but these enantiotopic protons are spectrally discriminable in an enantiopure, chiral cage, on the basis of a chemical shift difference. The methyl ^1^H NMR signals of 2-propanol and DMSO are discriminated because these molecules are prochiral and possess two enantiotopic methyl groups. In interaction with enantiomers of **[Co**_**3**_**L**_**2**_**]**, a new guest–host chiral enantiopure aggregate is formed; hence, the methyl groups of DMSO and 2-propanol as well as the methylene hydrogens of ethanol can be treated as if they were diastereotopic and so inequivalent. Spectrally, like any CH_2_ group near the stereogenic center in a chiral compound, they become distinguishable by NMR. This enantiotopic discrimination based on a difference of chemical shifts can be observed on both ^1^H and ^13^C nuclei of enantiotopic methyl groups (Figure 9). Such spectroscopic effects result from the combination of three effects: (i) the chirotopic surrounding of the guest molecule, (ii) tight fit and hindered rotation along the metal–donor atom bond, and (iii) strong paramagnetic effect of Co(II) ions. These factors also cause different extents of paramagnetic broadening of the signals of prochiral groups due to unequal average distances of these groups to paramagnetic Co(II) ions in the cage. Contrastingly, in the case of the acetone guest molecule, which is not prochiral and the methyl groups are homotopic, only broadening and gradual shift of the methyl signals is observed in the ^1^H NMR spectra without the enantiotopic effect (Figure S51). The enantiotopic discrimination of NMR signals was previously observed in the case of chiral liquid crystals used as NMR solvents^127−131^ but is very rare for interactions with metal complexes.^132−134^

The binding of guest molecules by **[Co**_**3**_**L**_**2**_**]** is not only enantioselective but also size-selective. The volume of the **[Co**_**3**_**L**_**2**_**]** host is limited and is equal to 139 Å^3^ for the crystalline form obtained from methanol and 168 Å^3^ for the crystalline form obtained from benzene (Figure S54). These volumes match well, for instance, the volume of the 2-butanol guest molecule equal to ca. 154 Å^3^. These volumes are also compatible with the volume of a pyridine molecule equal to ca. 134 Å^3^, but the ^1^H NMR data indicate that this potential guest is not bound. What is more important is the minimum diameter of the guest molecule in comparison with the diameter of the host cavity. The pore diameter reported in ref (24) for the isostructural Zn(II) barrel-shaped cage calculated with the py-window program is equal to 3.1 Å, and the window diameter (the entrance into the cavity between *tert*-butyl groups) is equal to 2.8 Å. These diameters are sufficient for the binding of a smaller guest such as methanol or linear guests such as 2- or 1-butanol of minimum diameter equal to approximately 3.3 Å, taking into account some flexibility of the host structure discussed above. On the other hand, guests such as pyridine (and other guests with aromatic rings), guests with *tert*-butyl groups, or amines such as triethylamine with the diameters of molecules equal to ca. 5.7, 6.0, and 7.5 Å, respectively, will not fit into the cavity of the **[Co**_**3**_**L**_**2**_**]** hosts. Thus, a strong paramagnetic effect reflecting binding within the cage molecule is observed for smaller alcohol molecules (methanol, ethanol, 1-propanol, 2-propanol, 1,2-propanediol, 1-butanol, 2-butanol, 1-pentanol, 2-pentanol) even at a low host/guest ratio, while the ^1^H NMR signals of cyclohexanol, *tert*-butanol, and 1-phenylethanol are practically not affected or minimally affected when **[Co**_**3**_**L**_**2**_**]** is used in the same low concentrations (Figures S47, S55, and S56). This difference reflects the larger diameters of these molecules, which are not compatible with the diameter of the cage cavity. Only much higher concentrations of **[Co**_**3**_**L**_**2**_**]** allow resolution of the ^1^H NMR signals of the two enantiomers of 1-phenylethanol (Figure S47). This weaker effect most likely reflects the interaction of these alcohol molecules with the outer walls of the barrel-shaped cage. It should be noted that similar weak C–H···F and C–H···O hydrogen bonding interactions between the outer substituents of **[Zn**_**3**_**L**_**2**_**]** and perfluoroalkyl carboxylic acids were observed in the solid state.^25^ This kind of interaction is likely responsible for the effective resolution of enantiomers of organic compounds by **[Zn**_**3**_**L**_**2**_**]** in GC chromatography and electrophoresis^26−28^ in the case of molecules that are too large to fit inside the cavity of this host. The effect of the size and shape selectivity in the binding of guest molecules is also manifested in their influence on the paramagnetic shifts of the host. For example, the addition of 1-butanol or enantiomers of 2-butanol causes distinct changes in the ^1^H NMR signals of the Co(II) host, while the addition of *t*-butanol has no effect (Figures S37 and S57). This is another confirmation that the bulkier *t*-butanol does not fit in a barrel-shaped **[Co**_**3**_**L**_**2**_**]** cage.

Analogous trends regarding the influence of the guest size on changes in NMR spectra were observed for amines and ketones. Smaller amines (2-aminobutane, 1-aminobutane, 1-aminopropane, and 1,2-diaminoethane) are clearly affected by the trinuclear Co(II) complex used at low concentrations. On the other hand, the ^1^H NMR spectra of the larger triethylamine or pyridine, which are very good ligands for Co(II), practically do not change (Figure S58). Similarly, the addition of small amounts of the **[Co**_**3**_**L**_**2**_**]** complex practically does not result in the broadening or shift of ^1^H NMR signals of acetophenone and cyclopentanone (Figure S59), while the signals of smaller ketones acetone and 2-butanone are clearly shifted and broadened (Figures S51 and S52). The Co(II) host also influences ethyl acetate signals (Figure S53). In addition, the **[Co**_**3**_**L**_**2**_**]** cage can bind diethyl ether and *n*-hexane, as indicated by spectral changes when the complex is used at higher concentrations. The effects are relatively small, particularly for the latter guest, which reflects poor complexing ability and binding via VdW interactions only.

## Conclusions

The different coordination preferences of Zn(II), Co(II), Ni(II), and Cu(II) ions result in a remarkable difference in the kind of products derived from the [3 + 3] macrocycle **H**_**3**_**L**. Co(II) ions, similarly to Zn(II), form stable **[M**_**3**_**L**_**2**_**]** cage complexes with a distorted tetrahedral coordination sphere. In contrast, the Cu(II) and Ni(II) ions do not form stable trinuclear complexes with this macrocycle, and complexes of a smaller [2 + 2] macrocyclic Schiff base are formed instead. The latter macrocycle has N_2_O_2_ compartments suitable for metal ions with octahedral or tetragonal pyramidal geometry and is formed as a result of imine bond hydrolysis and rearrangement. The barrel-shaped **[Co**_**3**_**L**_**2**_**]** molecules have a central void and can accommodate small guest molecules. The guest binding in this system results from the combination of weak interactions and coordination to Co(II) centers and is strongest for linear coordinating molecules. Due to the restricted volume of the central void and the enantiopure nature of **[Co**_**3**_**L**_**2**_**]**, size-selective and enantioselective binding of organic guests is observed in solution. This enables spectroscopic enantiodifferentiation of racemic guests, facilitated by the paramagnetic influence of the high-spin cobalt(II) ions. The baseline resolution of ^1^H NMR signals is observed at host:guest ratios as low as 1:200 and is accompanied by little interference from the ^1^H NMR signals of the host molecule due to their broad nature. The chiral nature of the host also results in enantiotopic discrimination and splitting of ^1^H NMR signals of prochiral guests, such as ethanol or acetone. In light of the gas sorption properties of the Zn(II) analogue,^23,24^**[Co**_**3**_**L**_**2**_**]** may be an interesting material for gas sorption and separation. In particular, it is interesting whether potentially coordinating molecules such as O_2_, CO, or H_2_ will exhibit a higher tendency to interact with Co(II) ions in this kind of cage.

## Experimental Section

### Synthesis

#### H

_**3**_**L***^**R**^***·CH**_**3**_**CN** and **H**_**3**_**L***^**S**^***·CH**_**3**_**CN** have been obtained as described previously.^22^

**[Co**_**3**_**L***^**R**^*_**2**_**]·3H**_**2**_**O**: 1.402 g of **H**_**3**_**L***^**R**^***·CH**_**3**_**CN** (1.568 mmol) and 0.585 g of Co(CH_3_COO)_2_·4H_2_O (2.352 mmol) have been refluxed for 2 h in 75 mL of methanol with vigorous stirring under a nitrogen atmosphere. The mixture was cooled and left to stand for 2 days. The resulting orange-brown precipitate (723 mg) was filtered off, dried under vacuum, and stored in air. Concentration of the filtrate gave the second crop of the product (43 mg). Total yield: 50.6%.

Anal. calcd for C_108_H_144_Co_3_N_12_O_9_: C, 67.17; H, 7.52; N, 8.70. Found: C, 67.16; H, 7.85; N, 8.83. ^1^H NMR (CDCl_3_, 500 MHz): δ_H_ 443.6, 196.1, 53.49, 48.12, 10.89, 2.73, −2.35, −4.42, −5.29, −6.28, −8.65, −11.05, −39.6, −45.6, −57.3. ESI-MS: *m*/*z* 1877.9 [C_108_H_138_Co_3_N_12_O_6_]+H^+^, 939.5 [C_108_H_138_Co_3_N_12_O_6_]+2H^+^, and 626.7 [C_108_H_138_Co_3_N_12_O_6_]+3H^+^.

#### [Co

_**3**_**L***^**S**^*_**2**_**]·3H**_**2**_**O** has been obtained in the same fashion starting from **H**_**3**_**L***^**S**^***·CH**_3_**CN**.

In some attempted syntheses of Cu(II) and Ni(II) cages, the perchlorate salts were used. *Caution:* perchlorate derivatives of coordination compounds can be potentially explosive.

### Methods

The NMR spectra were recorded on Bruker Avance III 300, 500, and 600 MHz spectrometers. The elemental analyses were carried out on PerkinElmer 2400 CHN and Elementar CHNS Vario EL Cube elemental analyzers. Magnetic measurements were carried out with Quantum Design MPMSXL-5 and MPMS3 SQUID magnetometers. The direct current (dc) magnetic susceptibility measurements were carried out in the temperature range of 1.8–300 K with applied magnetic fields of 5000 Oe. The alternating current (ac) susceptibility measurements in a zero dc field and in dc fields of 1000 and 1500 Oe were performed with an oscillating magnetic field of 3 Oe at frequencies ranging from 1 to 960 Hz. Background corrections for the sample holder and the diamagnetic contribution estimated from the Pascal constants were applied. The positive-mode electrospray mass spectra of methanol solutions of the complexes were obtained using a Bruker microOTOF-Q instrument. The CD spectra of 5.5 × 10^–4^ M solutions in CDCl_3_ were measured on a Jasco J-715 spectropolarimeter using 0.1 cm cuvettes.

The binding constants have been obtained on the basis of ^1^H NMR titrations of 0.0040 M **[Zn**_**3**_**L***^**S**^*_**2**_**]** or **[Zn**_**3**_**L***^**R**^*_**2**_**]** solution in C_6_D_6_, (298 K) with aliquots of a solution of (*R*)-2-butanol and ^1^H NMR titrations of 0.0040 M **[Zn**_**3**_**L***^**S**^*_**2**_**]** or **[Zn**_**3**_**L***^**R**^*_**2**_**]** solution in C_6_D_6_, (298 K) with aliquots of a solution of (*S*)-2-aminobutane. The apparent 1:1 binding constant *K* values have been calculated on the basis of the obtained binding isotherms with the use of the online Bindfit program.^135,136^ In each calculation, a simultaneous fitting of the two δ values of the two most affected signals of aromatic protons of the Zn(II) host was applied, with 28 or 30 total data points, and 3 fitted parameters were used in each fitting.

### X-Ray Crystallography

X-ray data collection for single crystals **1**–**8** (see Table S1 for numbering) investigated here was performed using graphite monochromatic MoKα radiation on a four-circle κ geometry KUMA KM-4 diffractometer with a two-dimensional area CCD detector at 100(2) K. The ω-scan technique with Δω = 1.0° for each image was used for data collection. One image was used as a standard after every 50 images for monitoring the crystal stability and the data collection. No correction of the relative intensity variations was necessary. Data collections were made using the CrysAlis CCD program.^137^ Integration, scaling of the reflections, correction for Lorentz and polarization effects, and absorption corrections were performed using the CrysAlis Red program.^137^ The structures were solved by direct methods using SHELXT-2014^138^ and refined with anisotropic displacement parameters using the SHELXL-2014/7 program.^139^ The hydrogen atoms were introduced into their geometrical positions and refined with isotropic displacement parameters. In most crystals, it was possible to locate and refine the solvent molecules; however, in crystals **2** and **8**, it was not possible to locate all the solvent molecules. The correct modeling of the disordered molecules was not possible, and therefore, a “squeeze” procedure was used to remove the scattering contribution of these molecules, which could not be satisfactorily modeled. In crystal **7**, the 2-aminobutane guest is disordered and has two orientations with an occupancy of 0.5, both of which bind to different Co(II) ions of the **[Co**_**3**_**L***^**R**^*_**2**_**]** host. 2-Pentanol in crystal **8**, which is located outside the **[Co**_**3**_**L***^**R**^*_**2**_**]** cage, is disordered in two opposite positions, both with an occupation factor of 0.5. However, the carbon backbone of 2-pentanol in both orientations overlaps, but the hydroxyl groups have opposite orientations (the head-to-tail orientation of the two possible occupations of this alcohol molecule in the crystal). The final difference Fourier maps showed no peaks of chemical significance. The data collection parameters, crystallographic data, and final agreement parameters are collected in Table S1. The structures were visualized by using the DIAMOND 3.0 and MERCURY 3.5.1 programs.^140,141^

## Supporting Information Available

NMR spectra, views of crystal structures, magnetic data, binding isotherms, and visualization of cage voids (Figures S1–S59) (PDF)
