# Supplementary material for: Paramagnetic Cage-Type Co(II) Complexes of Chiral Macrocycles: Enantio- and Size-Selective Binding of Guest Molecules
Source: Inorg Chem. 2025 Feb 24;64(9):4236–49. doi: 10.1021/acs.inorgchem.4c03956 (PMC11898176; doi:10.1021/acs.inorgchem.4c03956)
Supplement: Supplementary file 1 — ic4c03956_si_001.pdf [file ic4c03956_si_001.pdf]

Supporting Information

Paramagnetic Cage-type Co(II) Complexes of Chiral Macrocycles: Enantio- and Size-selective binding of Guest Molecules

*Jan Janczak‡ and Jerzy Lisowski†**

†Department of Chemistry, University of Wrocław, 14 F. Joliot-Curie, 50-383 Wrocław, Poland.

‡Institute of Low Temperature and Structure Research, Polish Academy of Sciences, Okólna 2 str., 50-422, Wrocław, Poland.

E-mail: jerzy.lisowski@chem.uni.wroc.pl

Contents

Crystallographic Table S1……………………………..…………………………..…..…S2-S3

Crystal structure figures……………………………………………………….……S6-S14, S35-S37

NMR spectra………………………………………………S4, S5, S19, S20, S38-S43, S45-S47

Magnetic data figures………………………………………………………….…………S15-S18

^{1}H titration experiments and binding isotherms……………….……………..……..S21-S34

Visualisation of the voids in the Co(II) cage……………………….……….……….…………S44

References…………………………………………………………..…...…….….…………S48

Table S1. Crystallographic data for investigated complexes.

Cif-file name	**cotcc_(1)**	**COSTACH3_(2)**	**Corte100_(3)**	**co16_(4)**
CCDC Number	**2355629**	**2355630**	**2355631**	**2355632**
Compound	**$[Co_3L^{S}_2]\cdot 8(CH_3OH)\cdot 4(H_2O)$**	**$[Co_3L^{S}_2]\cdot 7(CHCl_3)$**	**$[Co_3L^{R}_2]\cdot 2(C_6H_6)\cdot 3(H_2O)$**	**$[Co_2L'(AcO)_3](AcO)\cdot AcOH\cdot 3(H_2O)$**
Crystallization from	methanol	chloroform	benzene	methanol
Chemical formula	$C_{116}H_{170}Co_3N_{12}O_{18}$	$C_{115}H_{145}Cl_{21}Co_3N_{12}O_6$	$C_{120}H_{150}Co_3N_{12}O_9$	$C_{90}H_{126}Co_4N_8O_{25}$
Formula Mass	2197.42	2712.67	2087.30	1955.70
Crystal system	monoclinic	monoclinic	orthorhombic	monoclinic
Space group	C2	I2	$C222_1$	$P2_1$
a/Å	18.858(2)	17.322(2)	17.8201(9)	17.027(2)
b/Å	24.308(3)	18.365(2)	27.4701(12)	15.327(1)
c/Å	15.913(2)	23.679(3)	24.3506(7)	18.372(3)
α/°	90.0	90.0	90.0	90.0
β/°	112.266(8)	90.93(1)	90.0	92.855(2)
γ/°	90.0	90.0	90.0	90.0
V/Å^3	6750.7(14)	7531.7(15)	11920.1(9)	4788.6(2)
Temperature/K	100(2)	100(2)	100(2)	100(2)
Z	2	2	4	2
No. of refls measured	25332	20890	57717	24026
No. of independent refls	12205	12318	12984	18957
No. of obs. refls ($I > 2\sigma(I)$)	4842	7958	5541	15688
R_{int}	0.0766	0.0494	0.0851	0.0187
Final R_1 for obs. ($I > 2\sigma(I)$)	0.0739	0.0724	0.1161	0.0622
Final $wR(F^2)$ for all data	0.1652	0.1549	0.2786	0.1685
S	1.005	1.022	1.067	1.005
Flack parameter	0.061(30)	0.014(15)	0.09(4)	0.033(12)
Largest peak, hole/eA$^{\circ\ -3}$	+0.407, -0.343	+1.311, -0.431	+0.834, -0.356	+1.675, -0.565

Table S1. (continuation)

Cif-file name	**CUP21_(5)**	**CUTA21_(6)**	**co2_(7)**	**6_2pen_auto_sq_(8)**
CCDC Number	**2355633**	**2355634**	**2355635**	**2355636**
Compound	**$[Cu(H_2L')(ClO_4)]$ (ClO_4)**	**$[Cu(H_2L')(CH_3OH)]$ $(ClO_4)_2\cdot CH_3OH$**	**$[Co_3L^R{}_2$(*S*-$CH_3CH(NH_2)$ $CH_2CH_3)]\cdot 2(C_6H_6)\cdot H_2O$**	**$[Co_3L^R{}_2$(*S*-$CH_3CH(OH)$ $CH_2CH_2CH_3)]\cdot C_5H_{12}O$**
Crystallization from	methanol	methanol	benzene/*rac*-2-aminobutane	Benzene/*rac* 2-pentanol
Chemical formula	$C_{36}H_{48}Cl_2CuN_4O_{10}$	$C_{38}H_{56}Cl_2CuN_4O_{12}$	$C_{124}H_{159}Co_3N_{13}O_7$	$C_{128}H_{171}Co_3N_{12}O_{13}$
Formula Mass	831.22	895.30	2120.42	2262.55
Crystal system	monoclinic	monoclinic	orthorhombic	hexagonal
Space group	$P2_1$	$P2_1$	$C222_1$	$P6_322$
a/Å	6.4484(2)	9.9642(2)	17.822(3)	19.2535(2)
b/Å	16.7708(3)	10.2208(3)	27.479(5)	19.2535(2)
c/Å	17.3899(4)	20.0065(7)	24.357(4)	21.5476(2)
α/°	90.0	90.0	90.0	90.0
β/°	97.990(2)	90.949(3)	90.0	90.0
γ/°	90.0	90.0	90.0	120
V/$Å^3$	1862.37(8)	2037.22(10)	11928(4)	6917.53(16)
Temperature/K	100(2)	100(2)	100(2)	100(2)
Z	2	2	4	2
No. of refls measured	33046	21236	52290	189656
No. of independent refls	16275	9653	13439	7069
No. of obs. refls ($I > 2\sigma(I)$)	12760	7138	7560	6675
R_{int}	0.0219	0.0366	0.0747	0.0479
Final R_1 for obs. ($I > 2\sigma(I)$)	0.0534	0.0653	0.0858	0.0672
Final $wR(F^2)$ for all data	0.1460	0.1818	0.1981	0.1902
S	1.004	1.169	1.012	1.022
Flack parameter	0.07(4)	0.08(4)	0.026(12)	0.011(4)
Largest peak, hole/eA° $^{-3}$	+1.786, -1.182	+1.369, -0.846	+0.418, -0.263	+0.485, -0.683

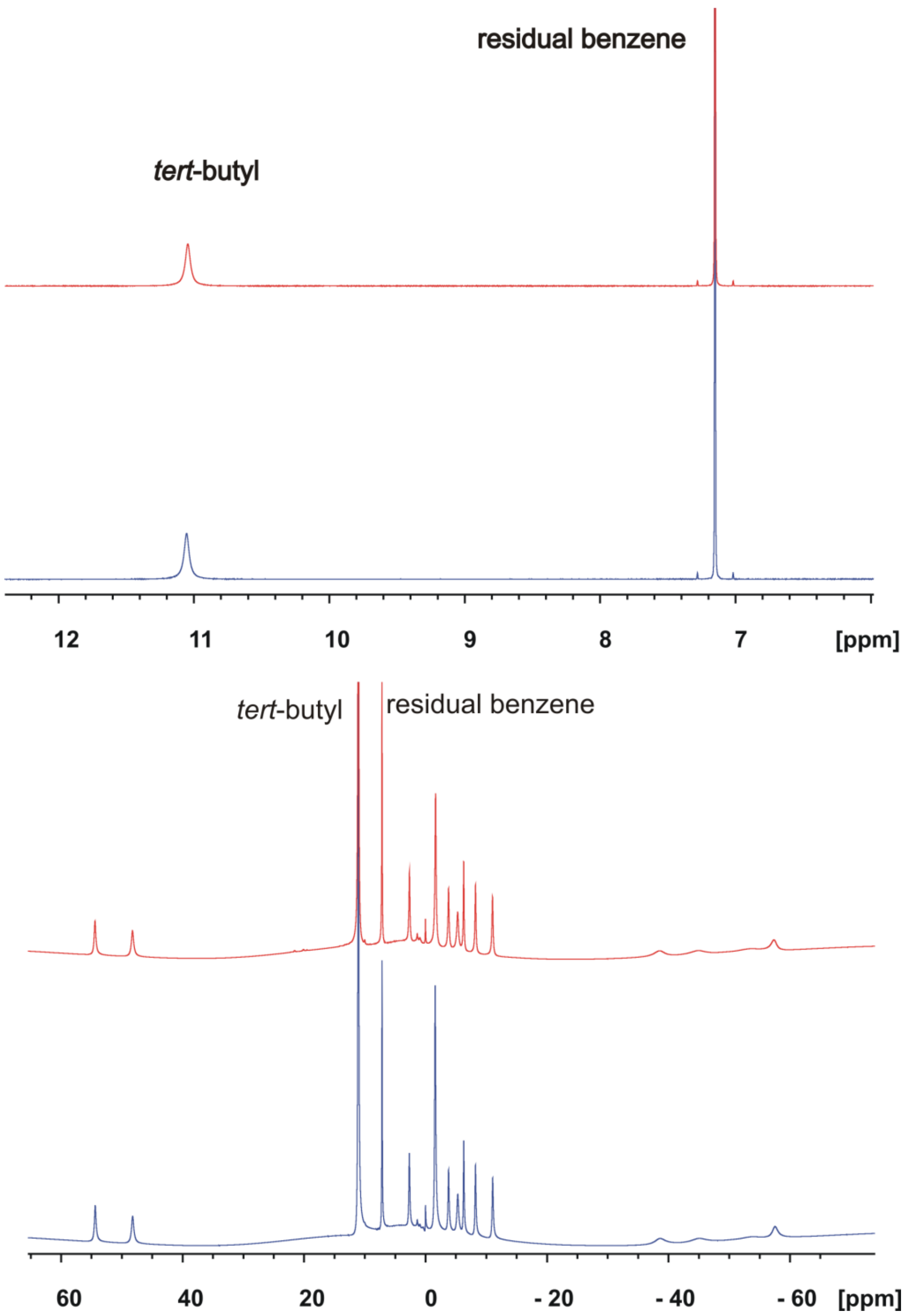

Figure S1. ^{1}H NMR spectra (300K, 600 MHz) of freshly prepared solution of **[Co_3L_2]** in C_6D_6 (blue) and the same solution after standing in aerobic conditions for 1 week (red). Top – the aromatic region of the ^{1}H NMR spectrum acquired with parameters typical for measurements of diamagnetic samples. Bottom – the paramagnetic region of the ^{1}H NMR spectrum recorded with parameters modified for paramagnetic sample (repetition time 50 ms, LB = 20, SW = 400 ppm).

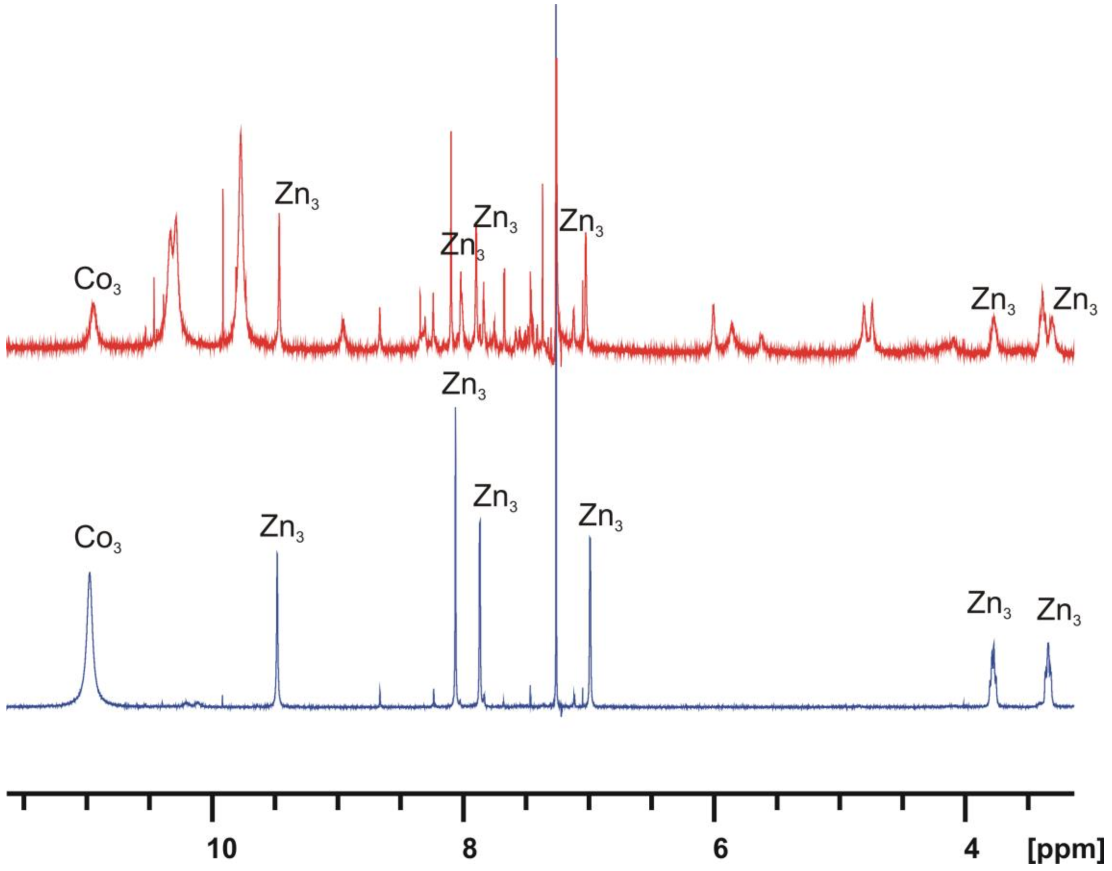

Figure S2. Top: Region of the ^{1}H NMR spectrum ($CDCl_3$ solution) of the product of the reaction between 2 equivalents of $\mathbf{H_3L^{\mathit{R}}}$ and a mixture of 1.5 equivalents of $Co(CH_3COO)_2{\cdot}4H_2O$ and 1.5 equivalents of $Zn(CH_3COO)_2{\cdot}2H_2O$. Label Co_3 indicate the signal of the [$\mathbf{Co_3L^{\mathit{R}}_2}$] complex and label Zn_3 indicates the signals of the **[**$\mathbf{Zn_3L^{\mathit{R}}_2}$**]** complex. The remaining signals correspond to the low-symmetry mixed species [$\mathbf{Co_2ZnL^{\mathit{R}}_2}$] and [$\mathbf{CoZn_2L^{\mathit{R}}_2}$].

Bottom: Region of the ^{1}H NMR spectrum ($CDCl_3$ solution) of the mixture of **[**$\mathbf{Co_3L_2}$**]** and **[**$\mathbf{Zn_3L_2}$**]** complexes kept for 1 day.

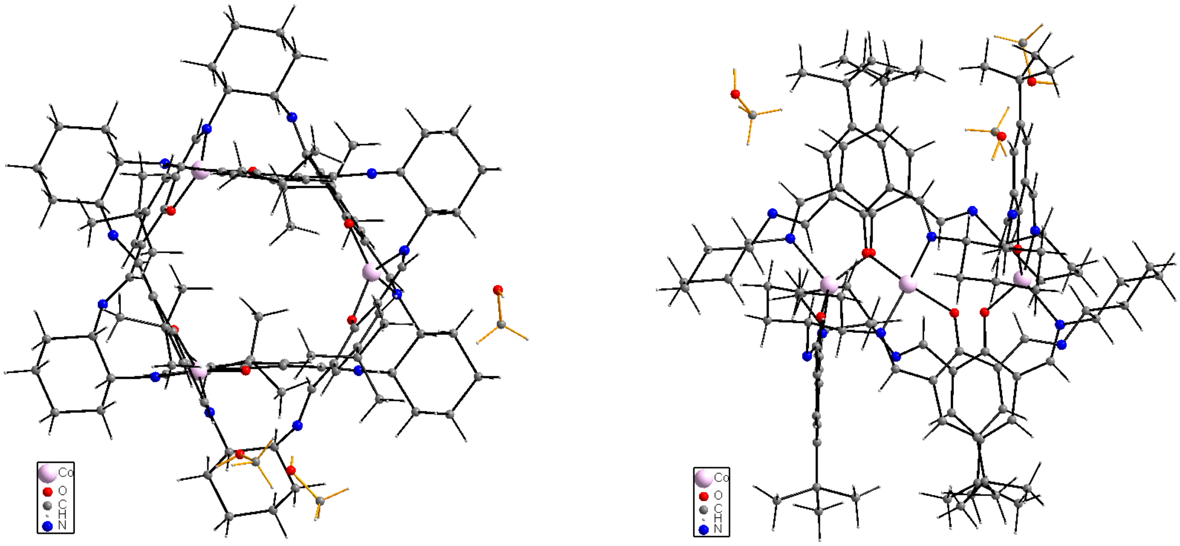

Figure S3. Top (left) and side (right) views of the $[Co_3L^{S}_2(CH_3OH)]\cdot 7CH_3OH\cdot 4H_2O$ complex crystallized from methanol (CH_3OH molecules indicated in orange).

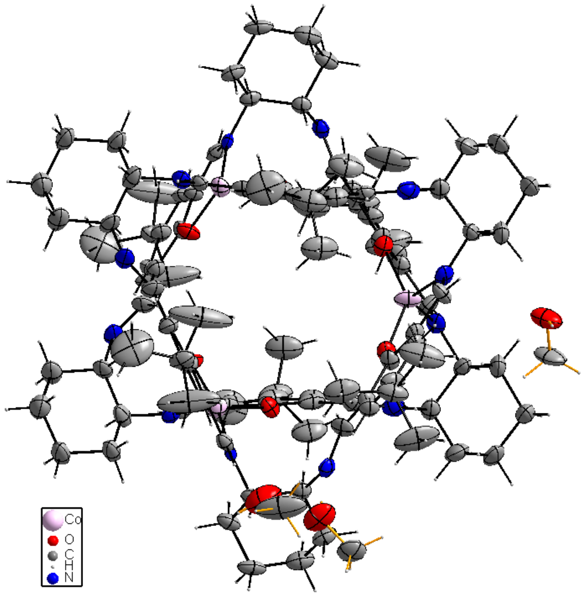

Figure S4. Top view of the $[Co_3L^{S}_2(CH_3OH)]\cdot 7CH_3OH\cdot 4H_2O$ complex with anisotropic displacement parameters.

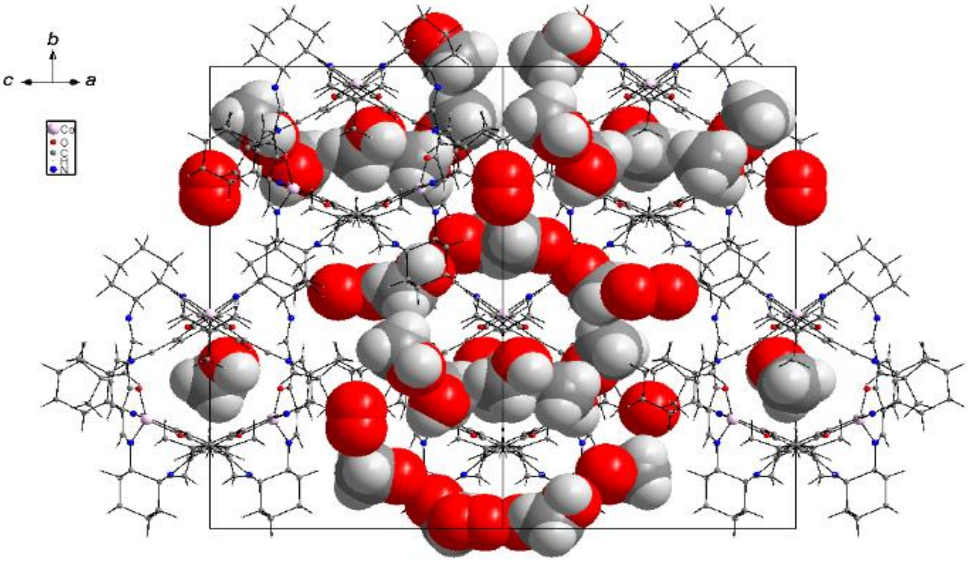

Figure S5. Packing of the $[Co_3L^{S}_2(CH_3OH)]\cdot 7CH_3OH\cdot 4H_2O$ complex (solvent molecules in spacefill representation).

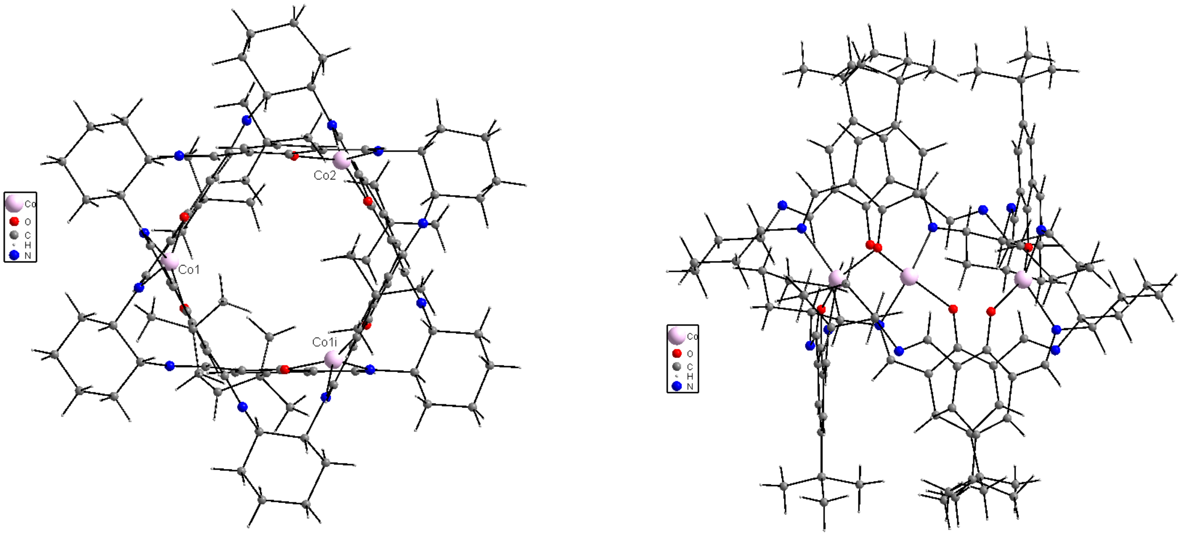

Figure S6. Top (left) and side (right) views of the $[Co_3L^{R}_2]\cdot 2C_6H_6\cdot 3H_2O$ complex crystallized from benzene.

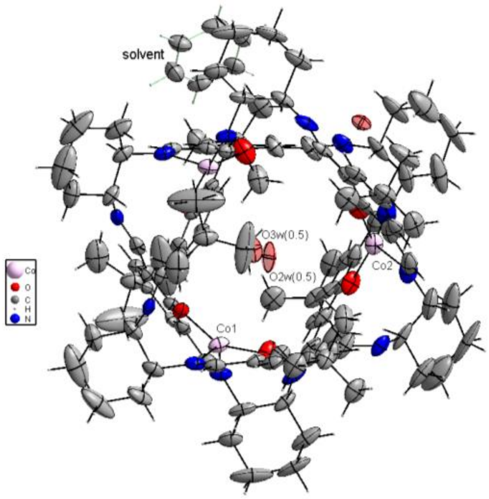

Figure S7. Top view of the $[Co_3L^R{}_2]\cdot 2C_6H_6\cdot 3H_2O$ complex with anisotropic displacement parameters

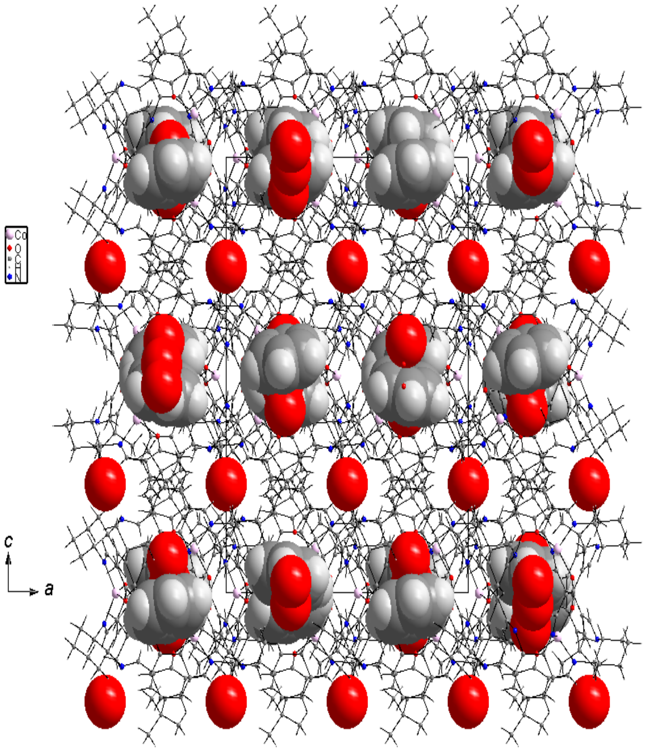

Figure S8. Packing of the $[Co_3L^R{}_2]\cdot 2C_6H_6\cdot 3H_2O$ complex (solvent molecules in spacefill representation)

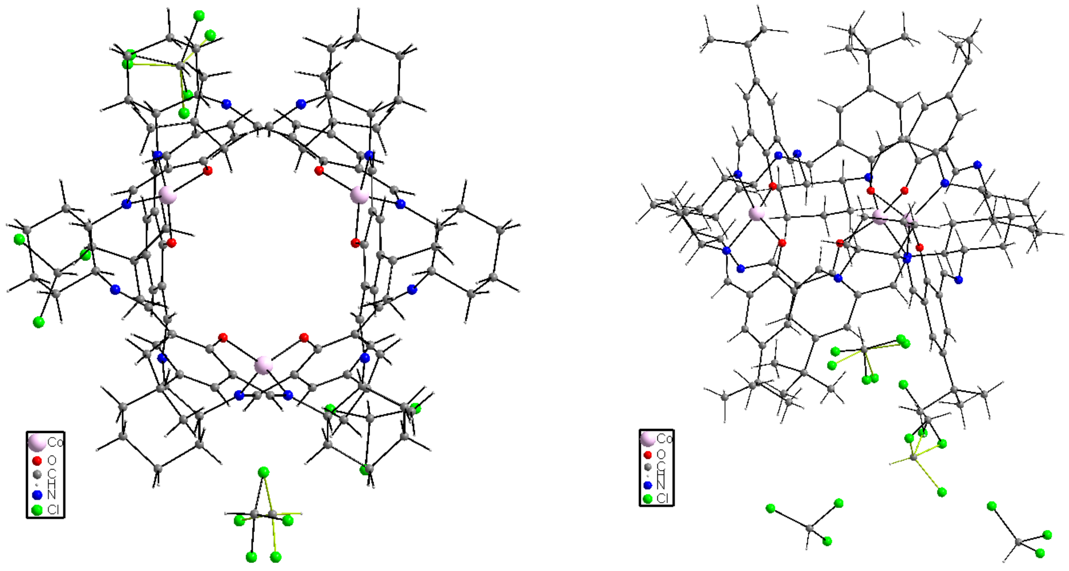

Figure S9. Top (left) and side (right) views of the $[Co_3L^{S}_2]\cdot 7CHCl_3$ complex crystallized from chloroform with disordered $CHCl_3$ molecules

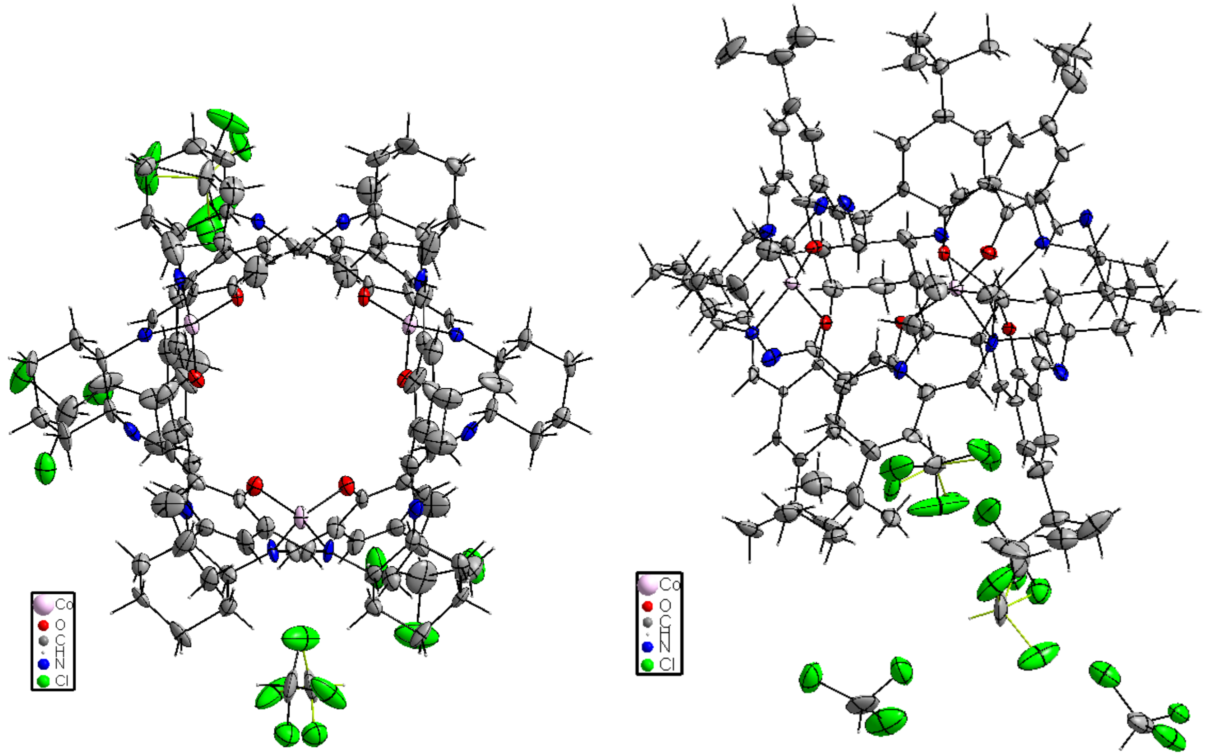

Figure S10. Top (left) and side (right) views of the $[Co_3L^{S}_2]\cdot 7CHCl_3$ complex with anisotropic displacement parameters.

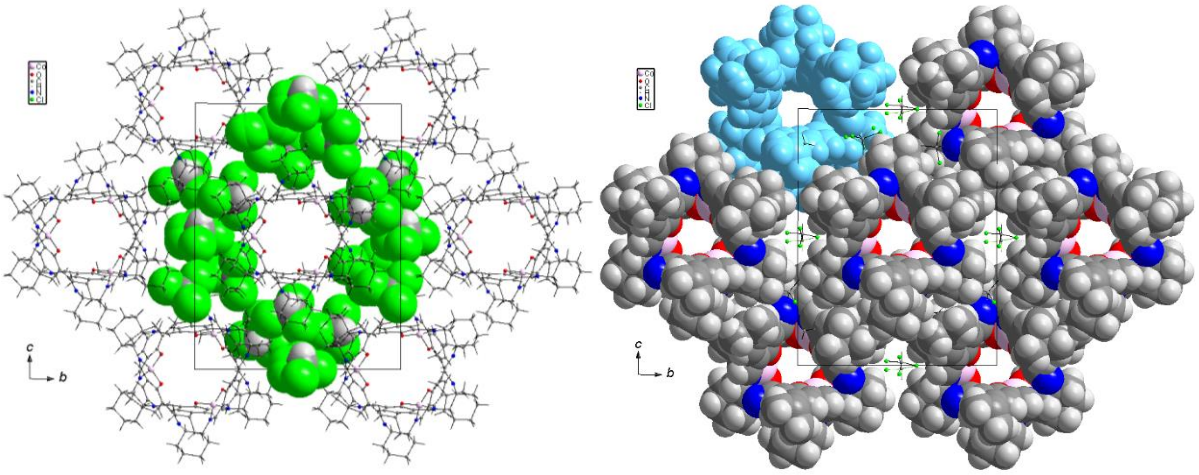

Figure S11. Left: Packing of the $[Co_3L^{S}_2]\cdot 7CHCl_3$ complex (solvent molecules in spacefill representation). Right spacefill representation of packing of the $[Co_3L^{S}_2]\cdot 7CHCl_3$ complex (single cage molecule indicated in light blue).

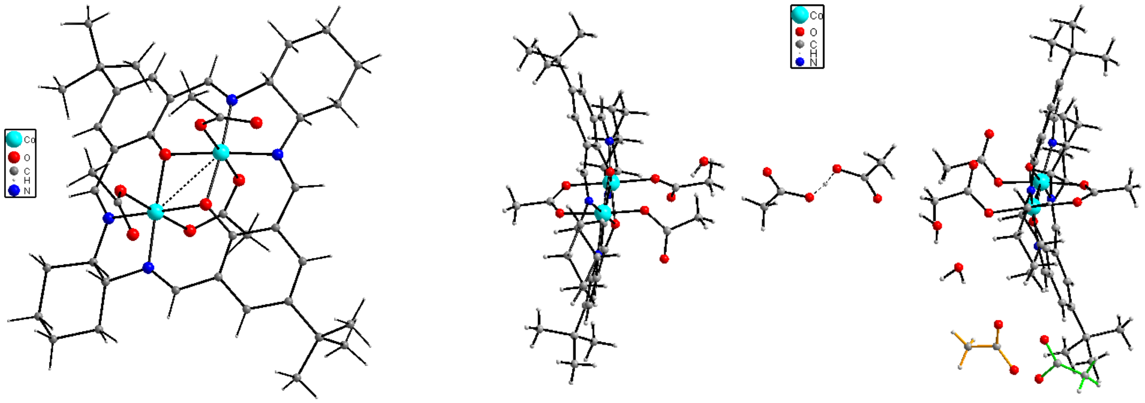

Figure S12. Views of the $[Co_2L'(AcO)_3](AcO)\cdot AcOH$ complex (the disordered CH_3COO^- anions indicated in green and yellow)

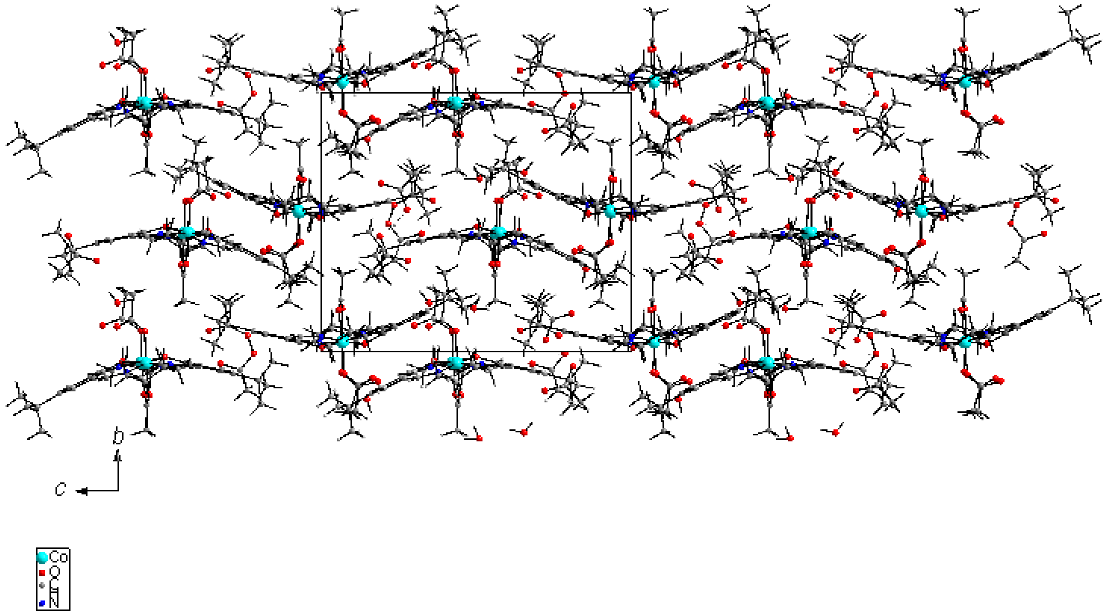

Figure S13. Packing of the $[Co_2L'(AcO)_3](AcO)\cdot AcOH$ complex.

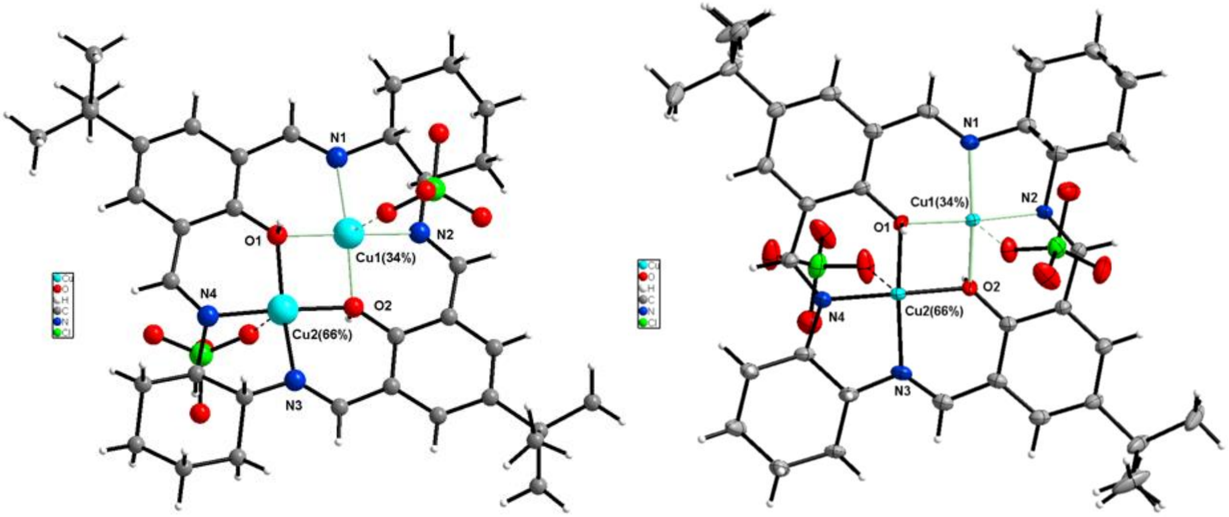

Figure S14. Views of the $[Cu(H_2L')(ClO_4)](ClO_4)$ complex.

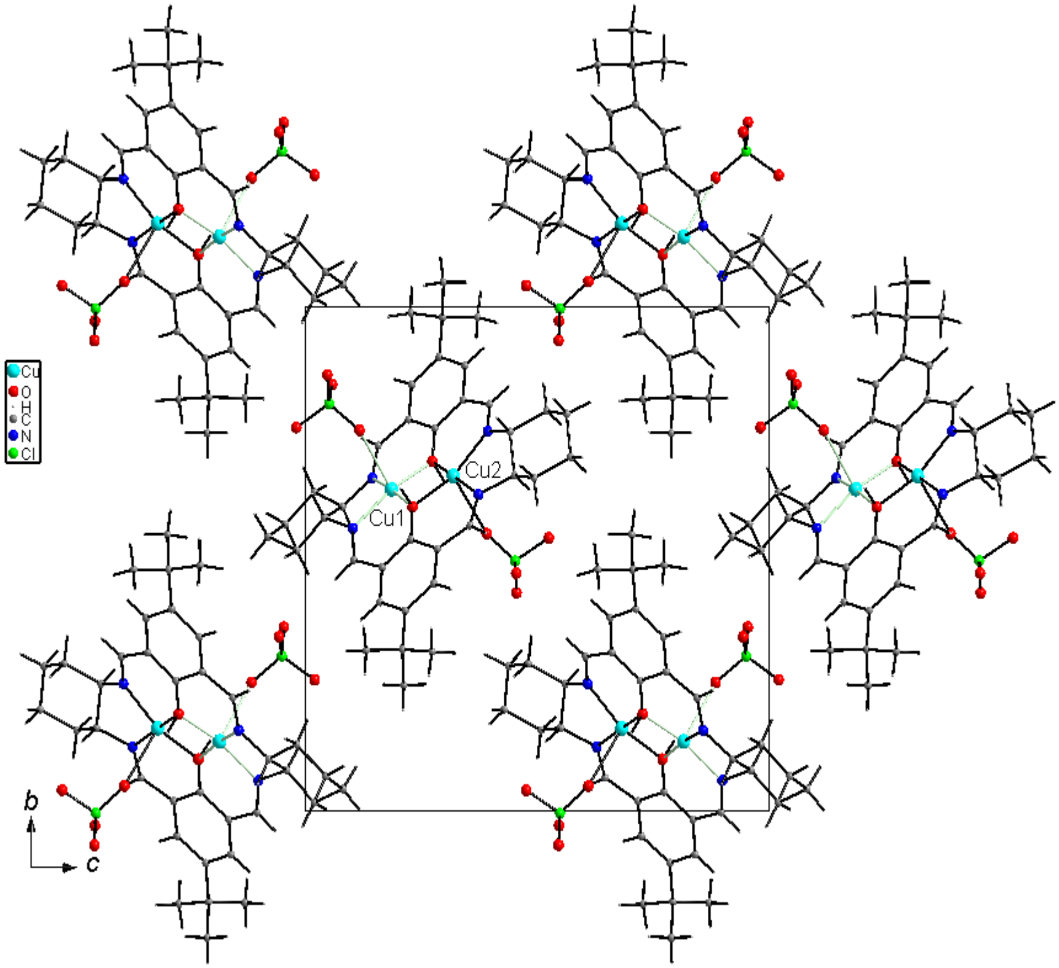

Figure S15. Packing of the [Cu(H_2L')(ClO_4)](ClO_4) complex.

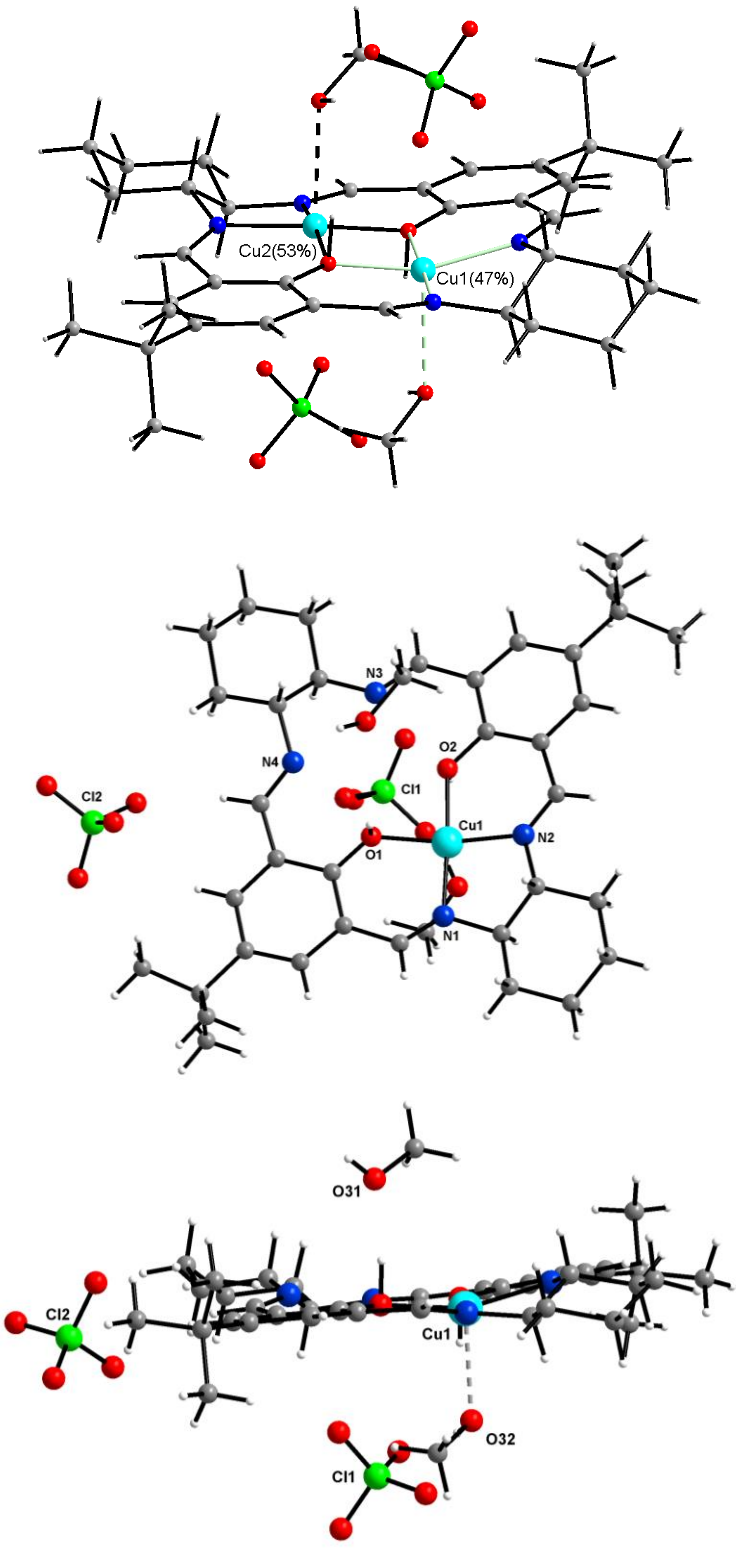

Figure S16. Views of the $[Cu(H_2L')(CH_3OH)](ClO_4)_2 \cdot CH_3OH$ complex.

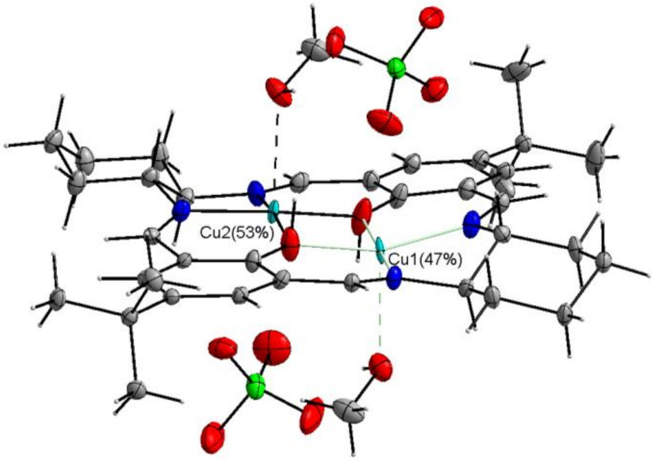

Figure S17. View of the $[Cu(H_2L')(CH_3OH)](ClO_4)_2 \cdot CH_3OH$ complex with anisotropic displacement parameters

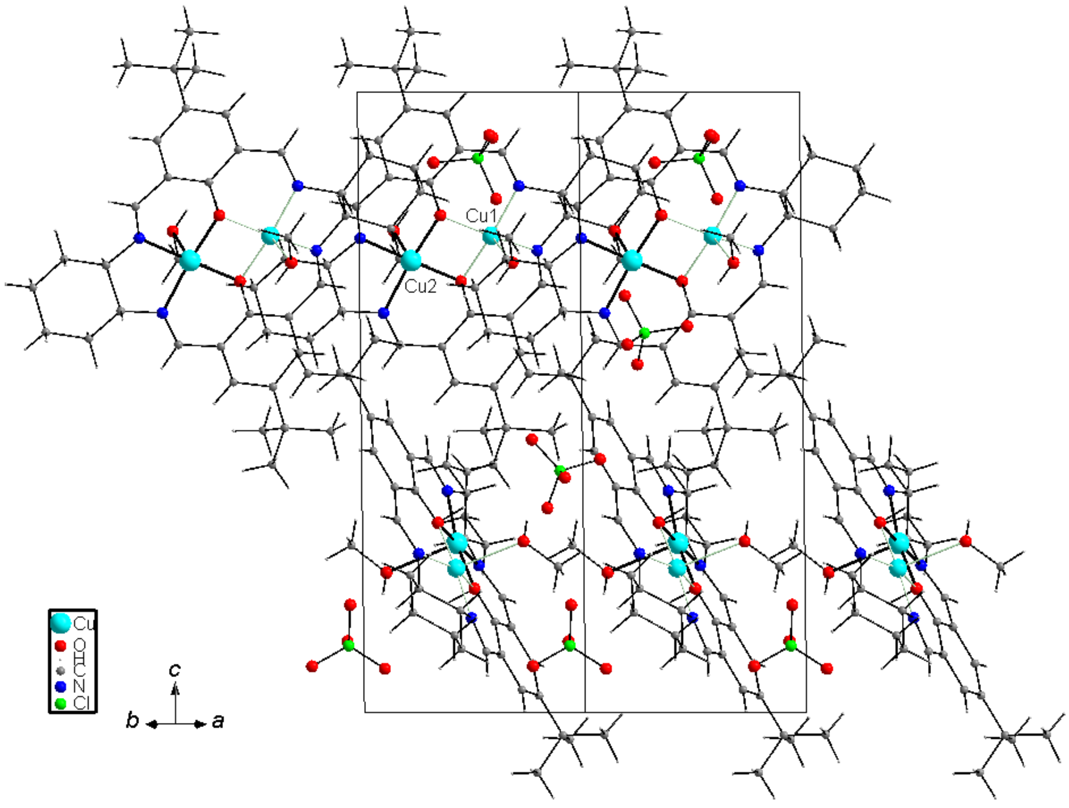

Figure S18. Packing of the $[Cu(H_2L')(CH_3OH)](ClO_4)_2 \cdot CH_3OH$ complex.

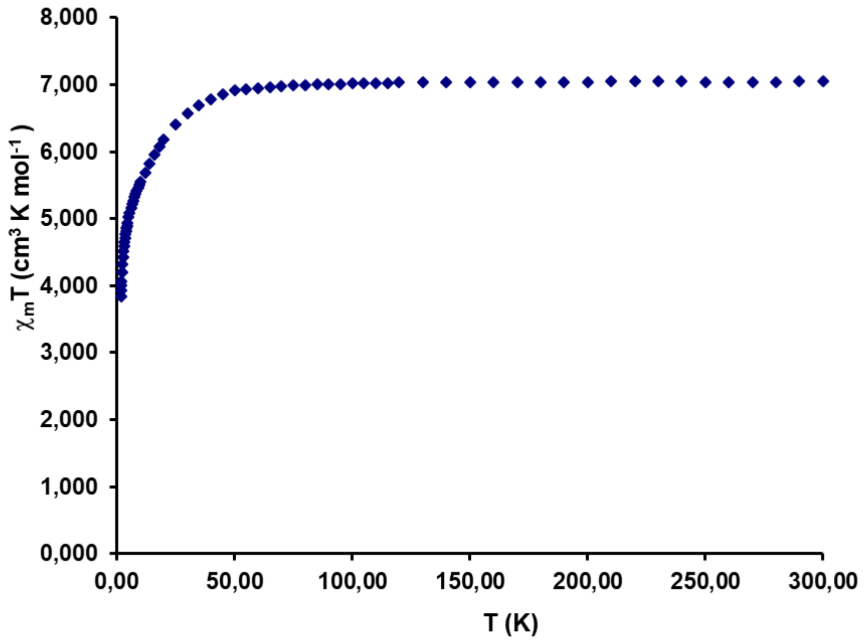

Figure S19. Plot of experimental $\chi_M T$ product vs. T for the $[Co_3L^R{}_2]$ complex.

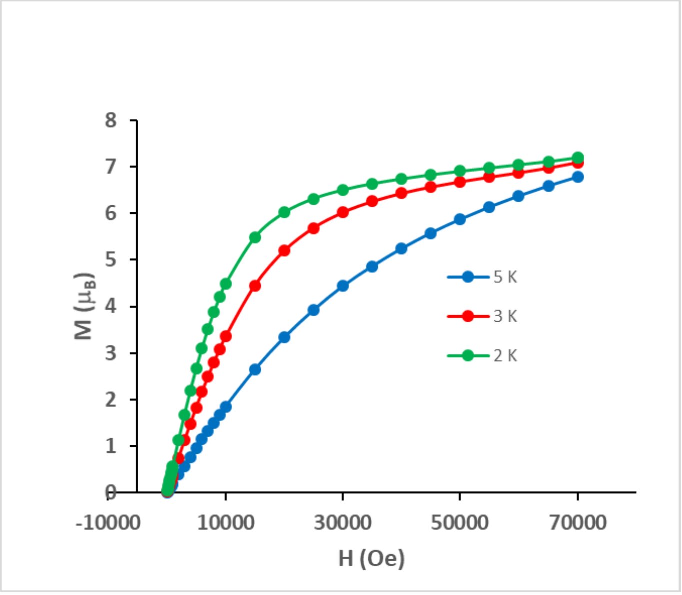

Figure S20. Plot of field dependence of the magnetization M vs H at 2, 3 and 5 K for the $[Co_3L^R{}_2]$ complex (the solid lines are eye guides only).

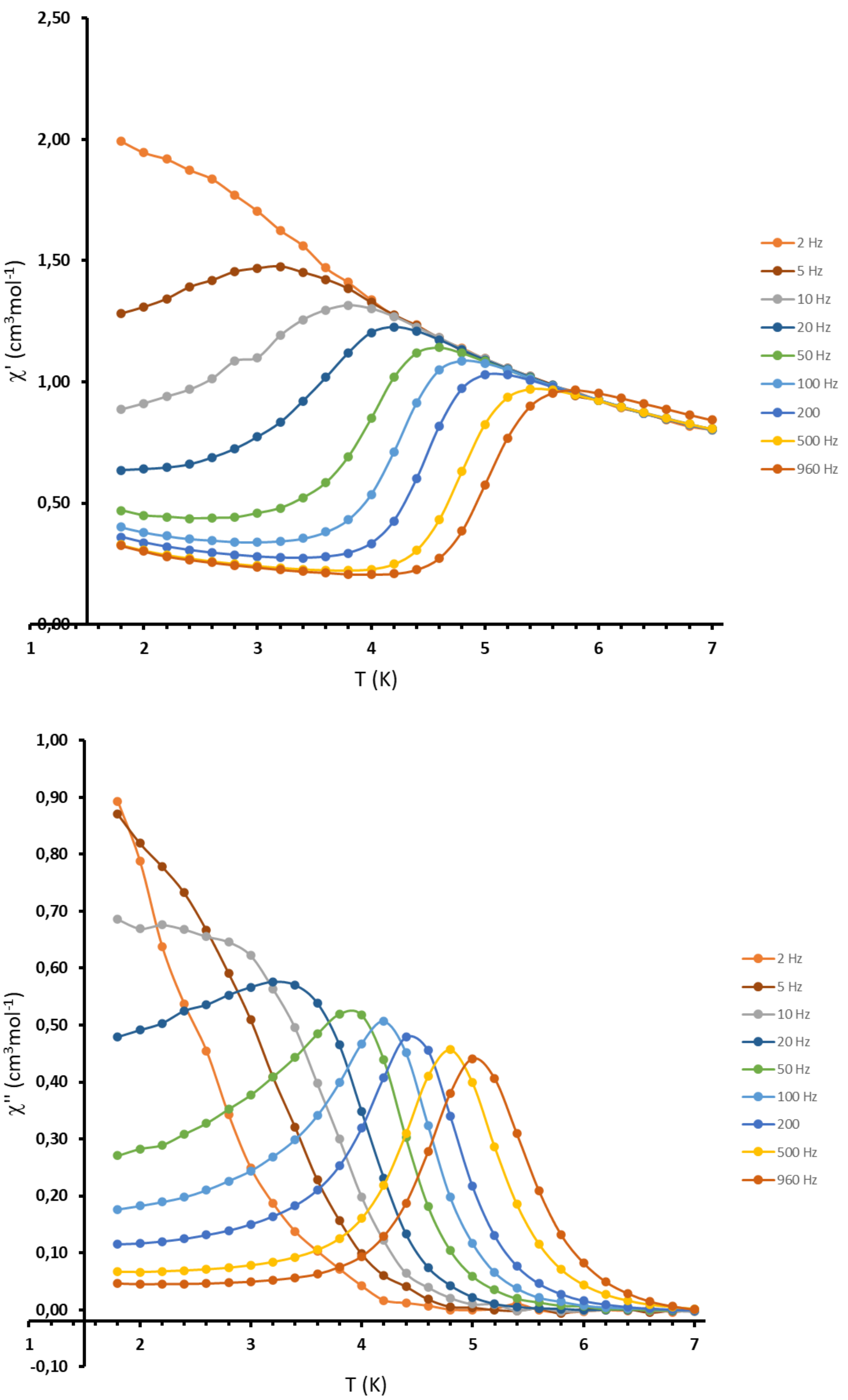

Figure S21. Temperatue dependence of the imaginary (χ'' top) and real (χ' bottom) components of ac susceptibility of the [$Co_3L^R{}_2$] complex at different *ac* frequencies between 1 and 960 Hz and different temperatures (3 Oe *ac* field and 1500 Oe *dc*-field, the solid lines are for the guidance only).

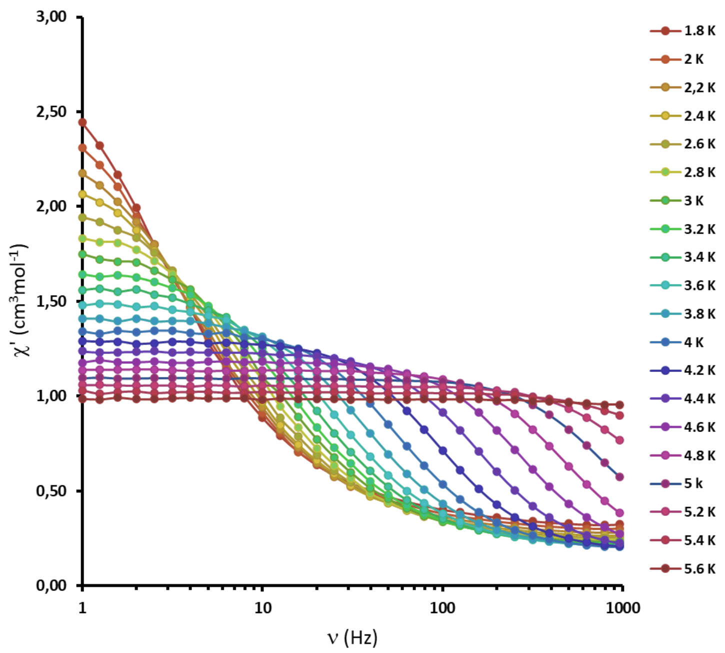

Figure S22. Frequency dependence of the real component of ac susceptibility (χ') for the [$Co_3L^R{}_2$] complex under 1000 Oe dc field at ac frequencies of 1-960 Hz in the temperature range of 1.8 to 5.6 K (the solid lines are eye guides only).

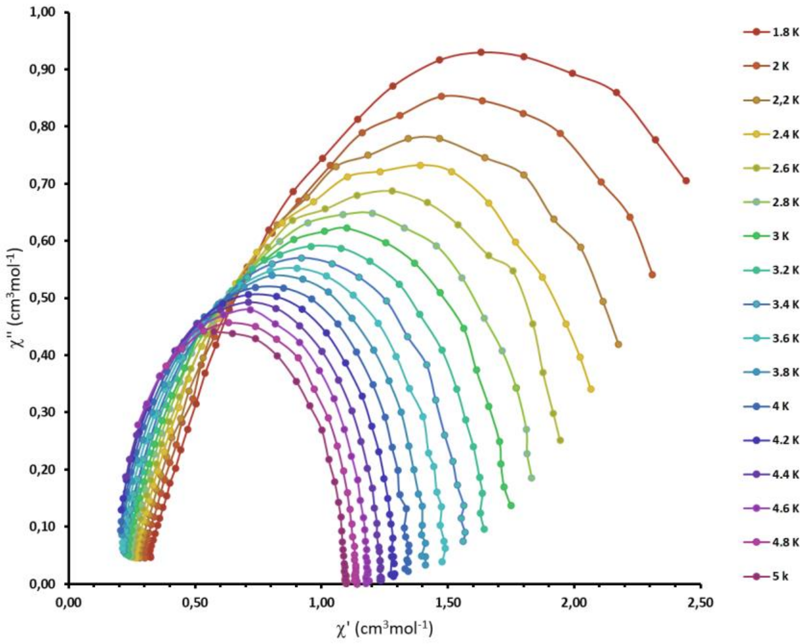

Figure S23. Cole-Cole plots for [$Co_3L^R{}_2$] (the solid lines are eye guides only).

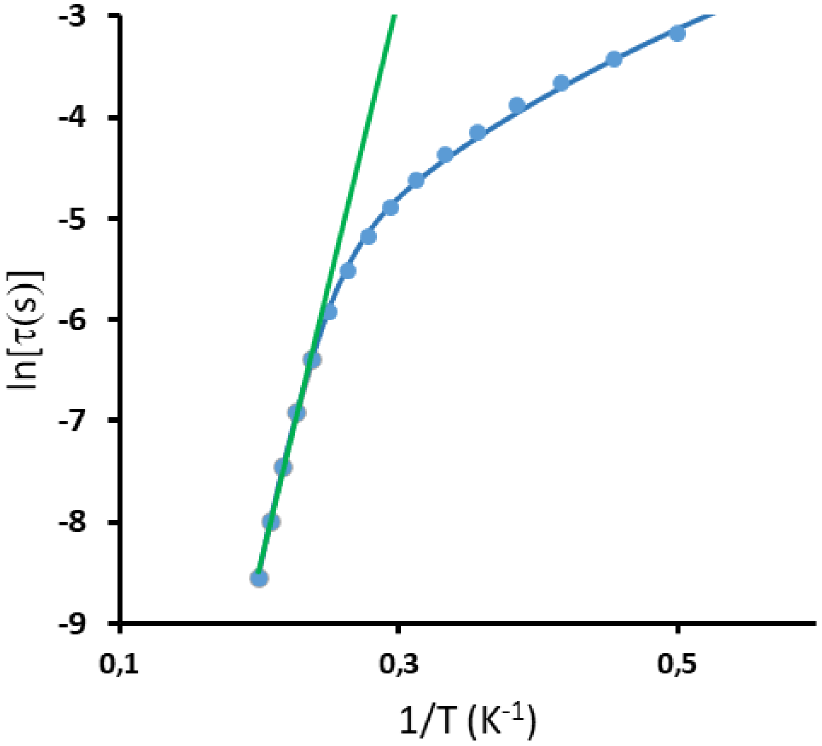

Figure S24. Temperature dependence of the logarithm of the magnetization relaxation time τ versus T^{-1} at applied 1500 Oe *dc*-field. Temperature dependent magnetic relaxation is in general dependent on multiple relaxation mechanism as given by the general equation

$$\tau^{-1} = \tau_0^{-1} exp(-U_{eff}/K_B T) + CT^n + \tau_{QTM}^{-}$$

where the three terms correspond to Orbach mechanism, Raman mechanism and quantum tunneling of magnetization, respectively.[1-4] Green line corresponds to Orbach process in accord with Arrhenius law and the blue line represents the fitting to the sum of Orbach and Raman processes with the following parameters: U_{eff} = 46.3 cm^{-1} (66.7 K), $\tau_0 = 3.5 \times 10^{-10}$ s, C = 2.5 s^{-1} K^{-n}, n = 3.2.

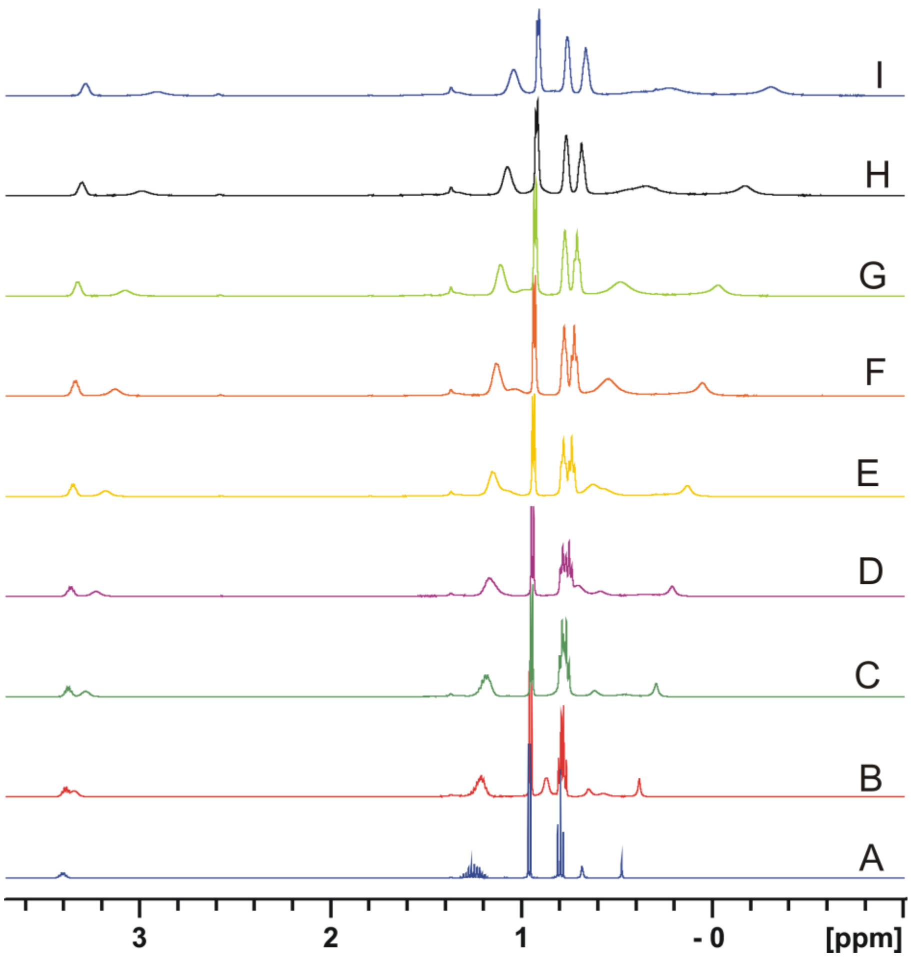

Figure S25. ^{1}H NMR titration of racemic 2-butanol (0.04 M solution in C_6D_6, 298K, 500 MHz) with increasing amounts of [$\mathbf{Co_3L^{R}_2}$] complex. Traces A – I correspond to 0, 0.0024, 0.0048, 0.0072, 0.0096, 0.012, 0.014, 0.019 and 0.024 equivalents of added Co(II) complex, respectively.

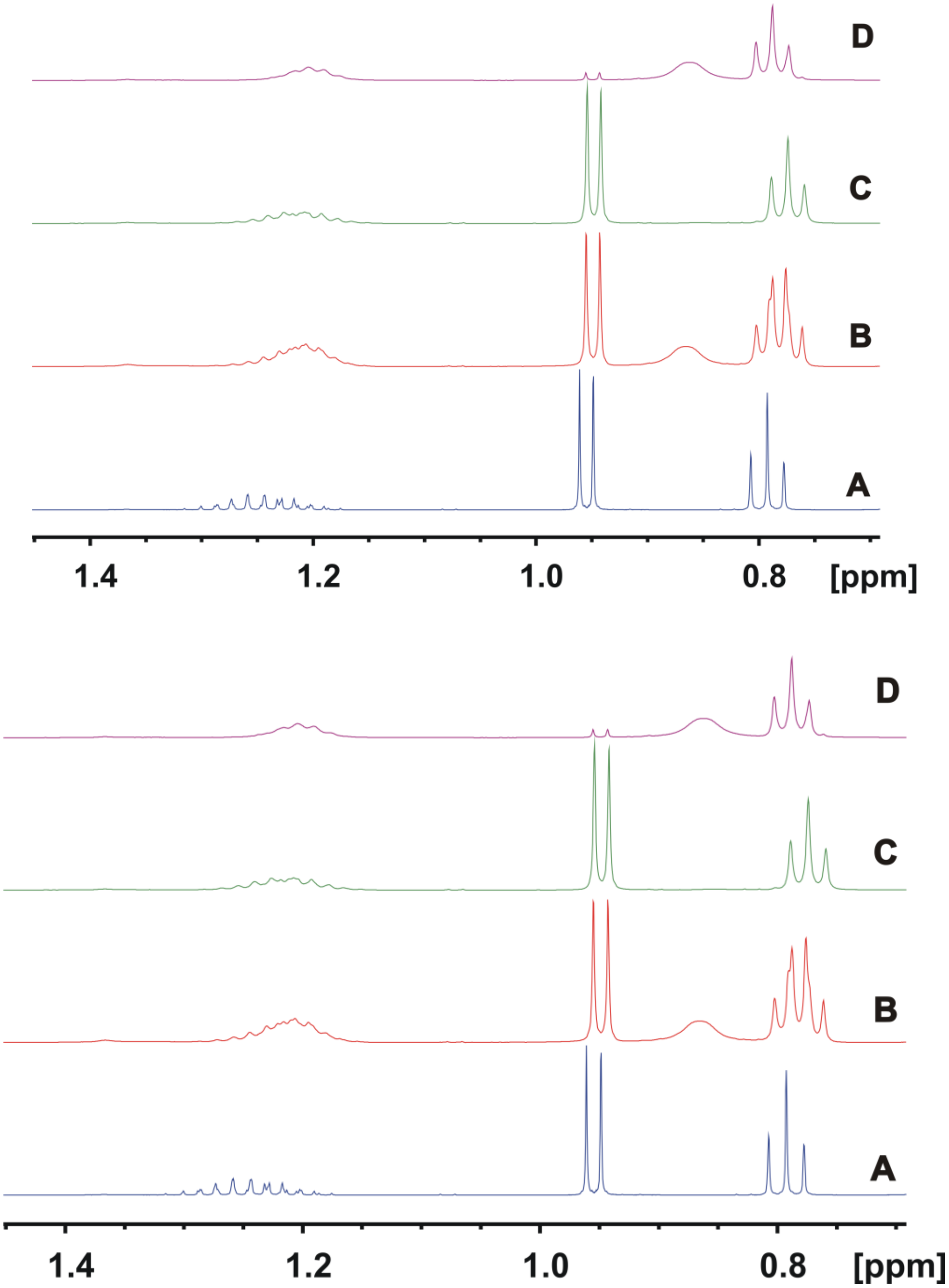

Figure S26. Top panel - $CH_3C\underline{\boldsymbol{H}}(OH)CH_2CH_3$ ^{1}H NMR signal of: A - racemic 2-butanol (0.04 M solution in C_6D_6, 298K, 500 MHz), B - racemic 2-butanol after addition 0.0096 equivalents of $[\mathbf{Co_3L^{\mathit{R}}_2}]$ complex, C – (*R*)-2-butanol after addition 0.0096 equivalents of $[\mathbf{Co_3L^{\mathit{R}}_2}]$ complex, D – (*S*)-2-butanol (ca. 95% ee) after addition 0.0096 equivalents of $[\mathbf{Co_3L^{\mathit{R}}_2}]$ complex.

Bottom panel: region of the ^{1}H NMR spectra of: A - racemic 2-butanol (0.04 M solution in C_6D_6, 298K, 500 MHz), B - racemic 2-butanol after addition 0.0024 equivalents of $[\mathbf{Co_3L^{\mathit{R}}_2}]$ complex, C – (*R*)-2-butanol after addition 0.0024 equivalents of $[\mathbf{Co_3L^{\mathit{R}}_2}]$ complex, D – (*S*)-2-butanol (ca. 95% ee) after addition 0.0024 equivalents of $[\mathbf{Co_3L^{\mathit{R}}_2}]$ complex. From left to right signals of protons $CH_3CH(OH)C\underline{\boldsymbol{H}}_2CH_3$, $C\underline{\boldsymbol{H}}_3CH(OH)CH_2CH_3$ and $CH_3CH(OH)CH_2C\underline{\boldsymbol{H}}_3$, respectively.

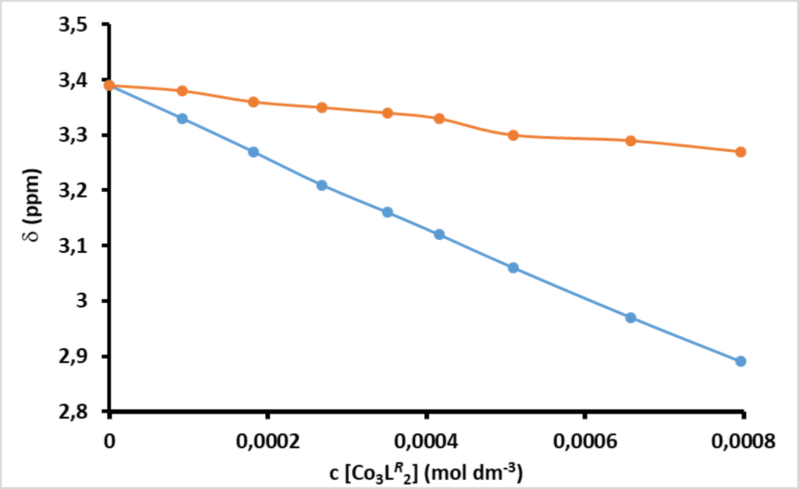

Figure S27. Dependence of the chemical shift of the $CH_3C\underline{\boldsymbol{H}}(OH)CH_2CH_3$ ^1H NMR signal of 2-butanol (0.04 M solution in C_6D_6) on the $[Co_3L^{R}_2]$ concentration for the enantiomers of 2-butanol: orange – (*R*)-2-butanol, blue – (*S*)-2-butanol.

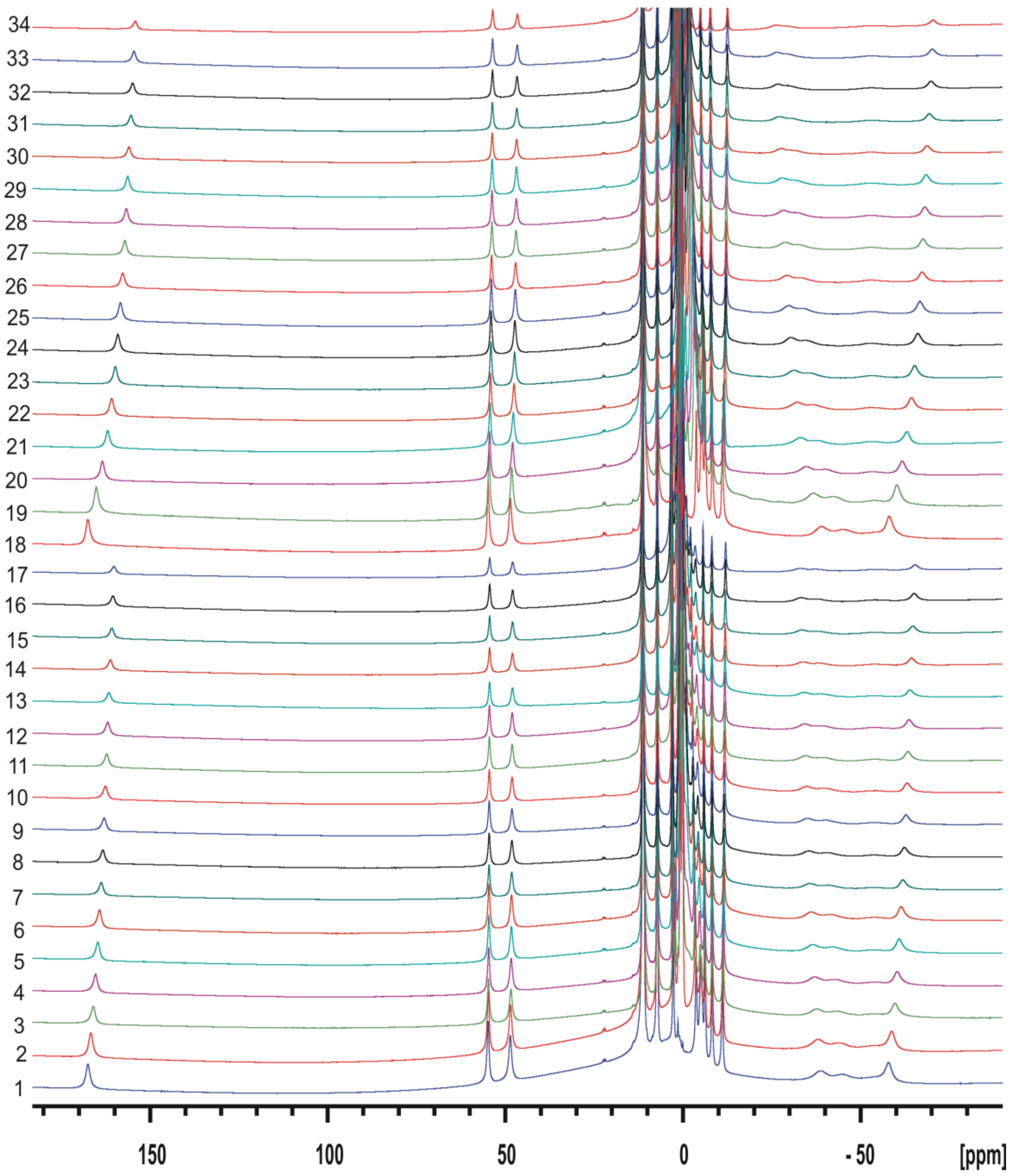

Figure S28.

Traces 1 – 17. ^{1}H NMR titration of 0.0040 M [$\mathbf{Co_3L^{R}_2}$] solution in C_6D_6, (300 K, 300 MHz) with increasing amount of (*R*)-2-butanol: 0, 4.53, 9.06, 13.59, 18.12, 22.65, 27.18, 31.71, 36.24, 40.77, 45.30, 49.83, 54.36, 63.42, 72.48, 81.54 and 90.60 equivalents of added guest, respectively.

Traces 18 – 34. ^{1}H NMR titration of 0.0040 M [$\mathbf{Co_3L^{R}_2}$] solution in C_6D_6, (300 K, 300 MHz) with increasing amount of (*S*)-2-butanol: 0, 4.53, 9.06, 13.59, 18.12, 22.65, 27.18, 31.71, 36.24, 40.77, 45.30, 49.83, 54.36, 63.42, 72.48, 81.54 and 90.60 equivalents of added guest, respectively.

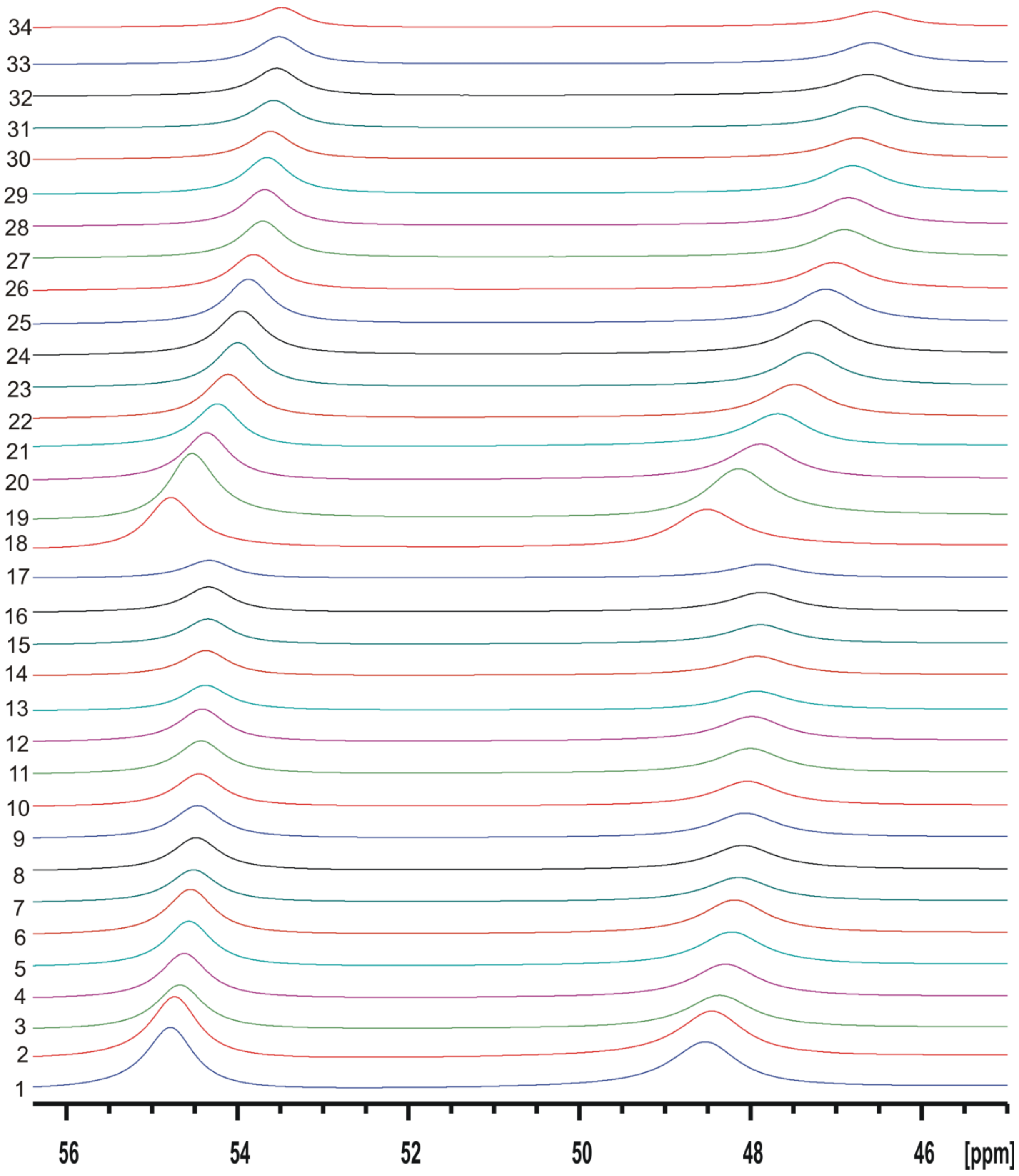

Figure S29. Expanded region of Figure S28, corresponding to two signals denoted as Ar1 and Ar2. These signals can be tentatively assigned to aromatic protons in [**Co₃L*ᴿ*₂**]. The tentative assignment is based on the analysis of the line widths, which are inversely proportional to the sixth power of the proton – paramagnetic ion distance.

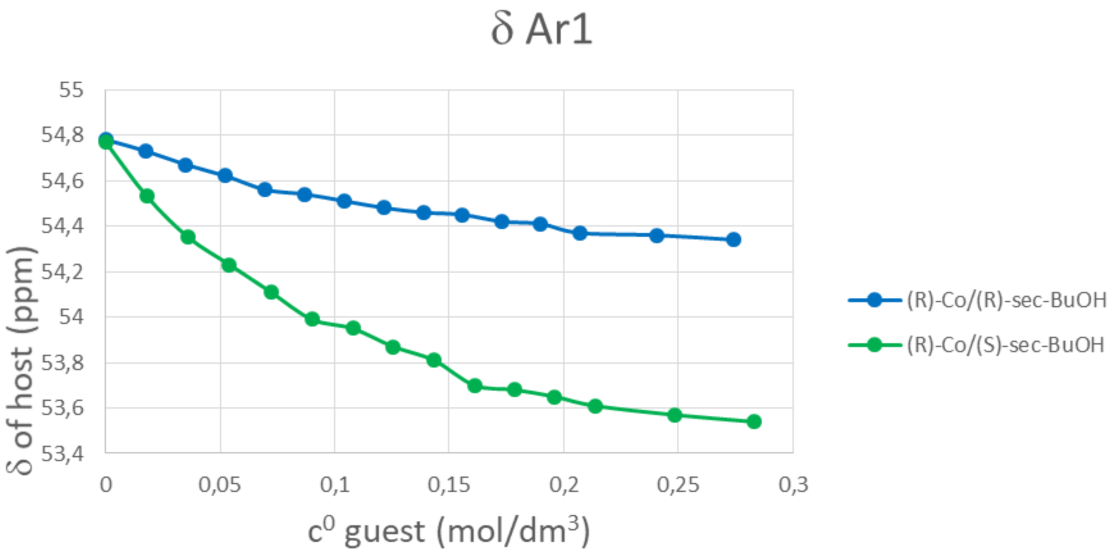

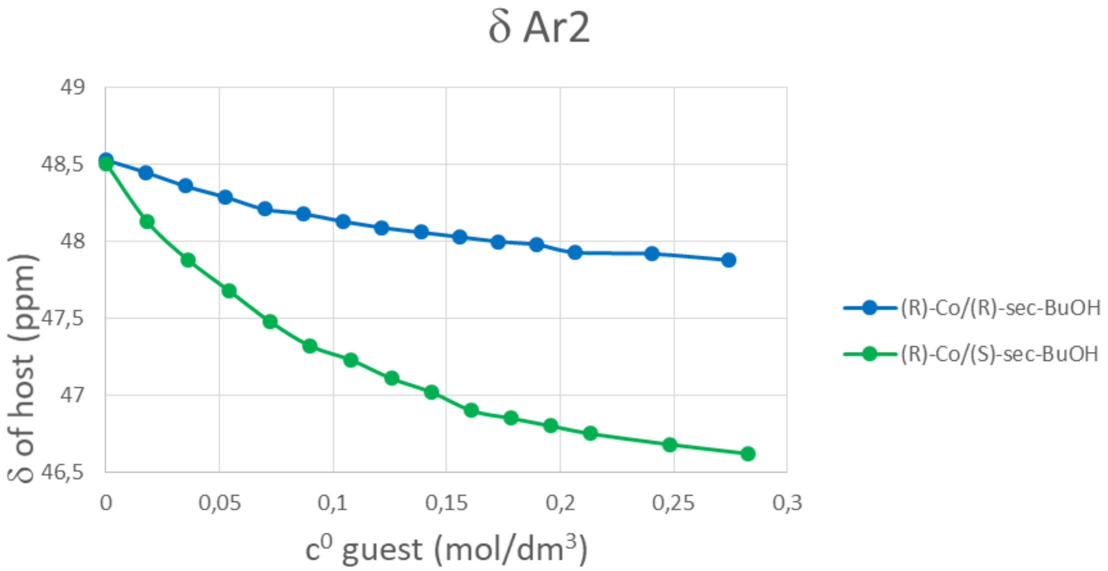

Figure S30. Variations of the chemical shift of the tentatively assigned aromatic signals Ar1 and Ar2 of the paramagnetic Co(II) cage host (0.0040 M [$\mathbf{Co_3L^{\mathit{R}}_2}$] solutions in C_6D_6, 300 K, 300 MHz) during ^{1}H NMR titration experiments presented in Figures S28, S29:

Blue (R)-Co/(R)-sec-BuOH – the (*R*)-2-butanol⊂[$\mathbf{Co_3L^{\mathit{R}}_2}$] pair

Green (R)-Co/(S)-sec-BuOH – the (*S*)-2-butanol⊂[$\mathbf{Co_3L^{\mathit{R}}_2}$] pair

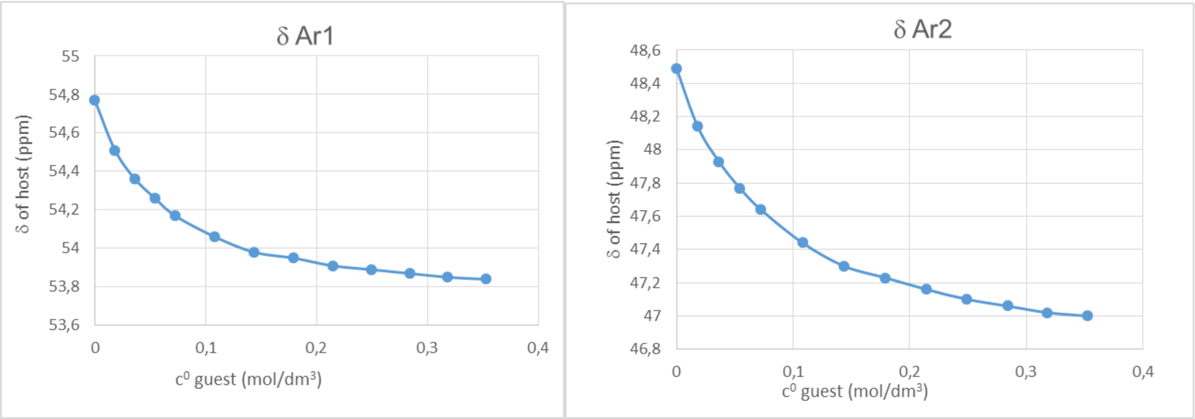

Figure S31. Variations of the chemical shift of the tentatively assigned aromatic signals Ar1 and Ar2 of the paramagnetic Co(II) cage host (0.0040 M $[\mathbf{Co_3L^{\mathit{R}}_2}]$ solutions in C_6D_6, 300 K) during ^1H NMR titration with 1-butanol.

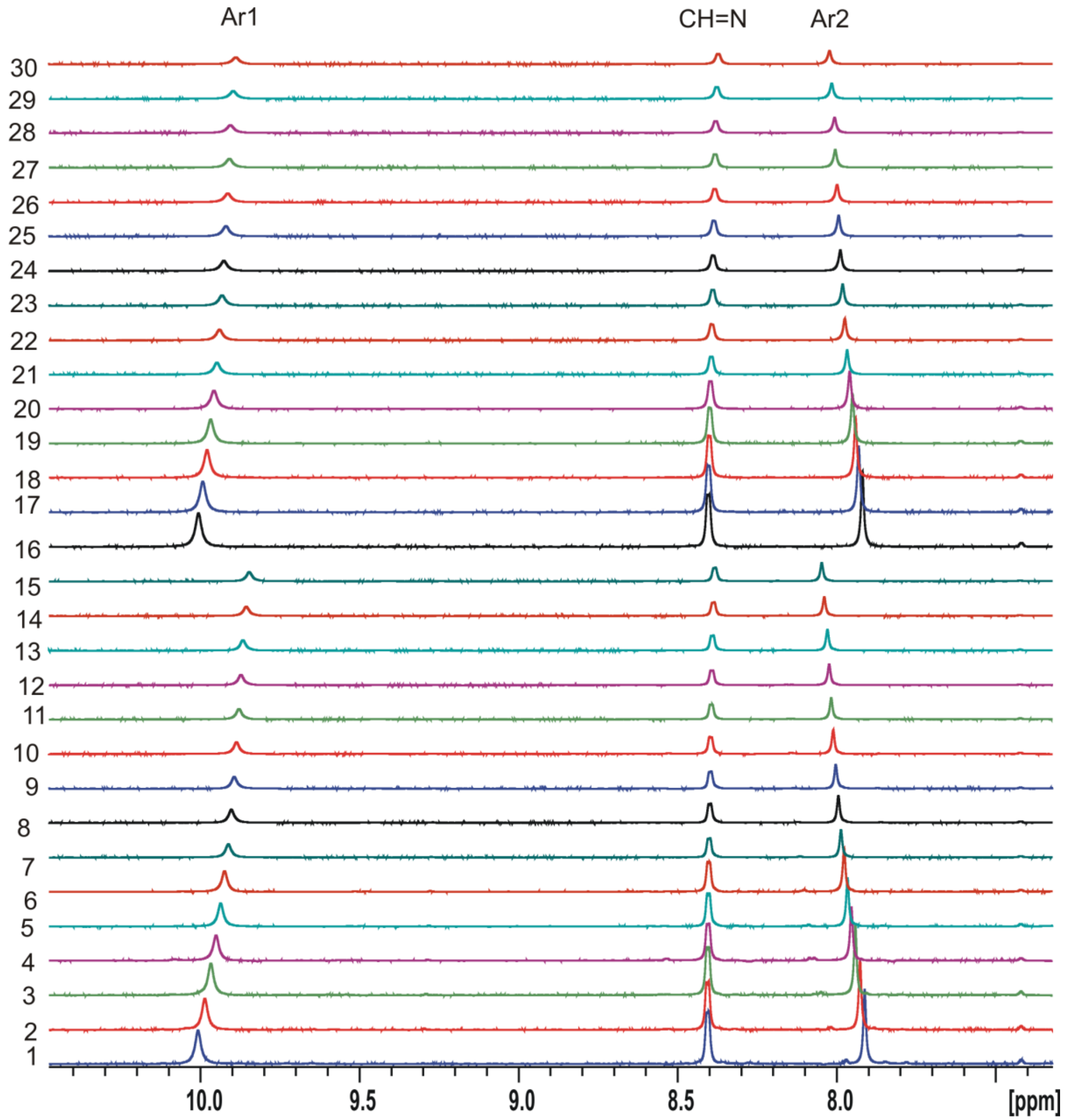

Figure S32. Traces 1 – 15. ^{1}H NMR titration of 0.0040 M [$\mathbf{Zn_3L^{\mathit{S}}_2}$] solution in C_6D_6, (298 K, 600 MHz) with increasing amount of (*R*)-2-butanol: 0, 8.78, 17.39, 25.84, 34.14, 42.28, 50.27, 58.11, 65.82, 73.39, 80.82, 88.13, 95.30, 109.29 and 122.81 equivalents of added guest, respectively.

Traces 16 – 30. ^{1}H NMR titration of 0.0040 M [$\mathbf{Zn_3L^{\mathit{R}}_2}$] solution in C_6D_6, (298 K, 600 MHz) with increasing amount of (*R*)-2-butanol: 0, 8.78, 17.39, 25.84, 34.14, 42.28, 50.27, 58.11, 65.82, 73.39, 80.82, 88.13, 95.30, 109.29 and 122.81 equivalents of added guest, respectively.

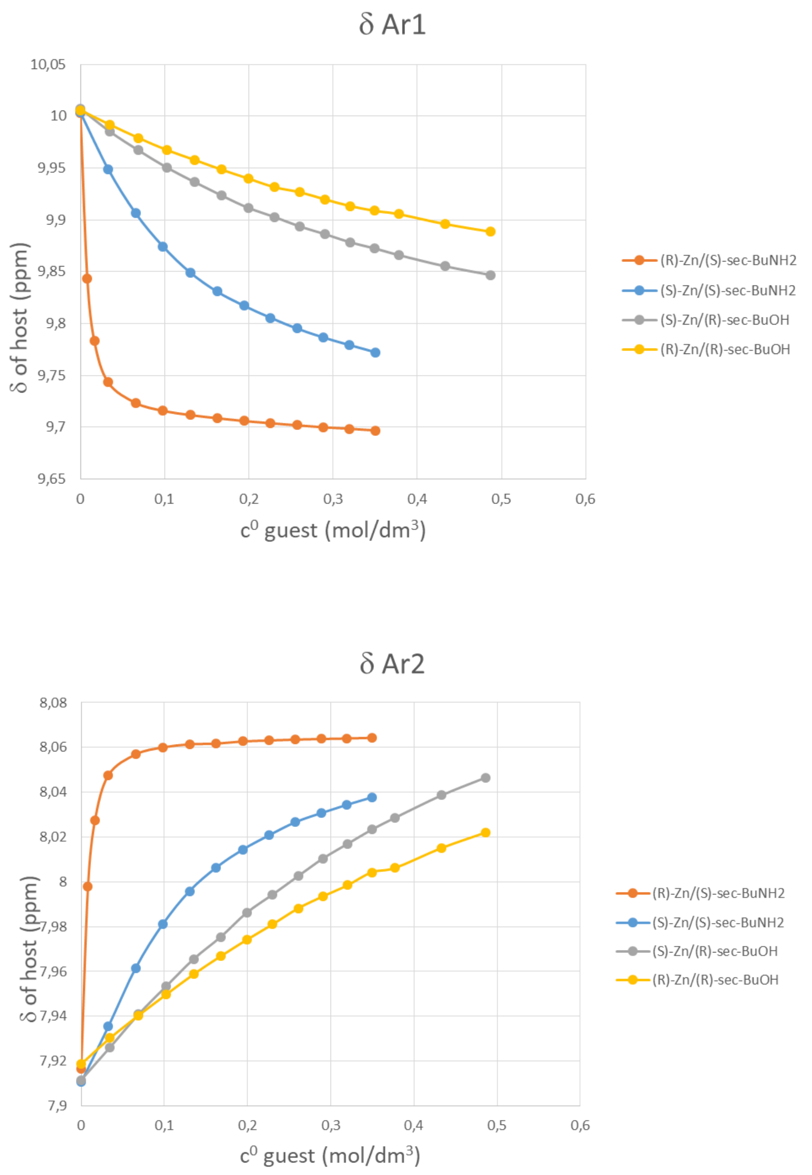

Figure S33. Variations of the chemical shift of the aromatic signals Ar1 and Ar2 of the diamagnetic Zn(II) cage host (0.0040 M $[\mathbf{Zn_3L_2}]$ solutions in C_6D_6, 298 K) during ^{1}H NMR titration experiments presented in Figures S40 and S44:

Orange-red (R)-Zn/(S)-sec-BuNH2 – the (*S*)-2-aminobutane⊂$[\mathbf{Zn_3L^{\mathit{R}}_2}]$ pair

Blue (S)-Zn/(S)-sec-BuNH2 – the (*S*)-2-aminobutane⊂$[\mathbf{Zn_3L^{\mathit{S}}_2}]$ pair

Gray (S)-Zn/(R)-sec-BuOH – the (*R*)-2-butanol⊂$[\mathbf{Zn_3L^{\mathit{S}}_2}]$ pair

Yellow (R)-Zn/(R)-sec-BuOH – the (*R*)-2-butanol⊂$[\mathbf{Zn_3L^{\mathit{R}}_2}]$ pair

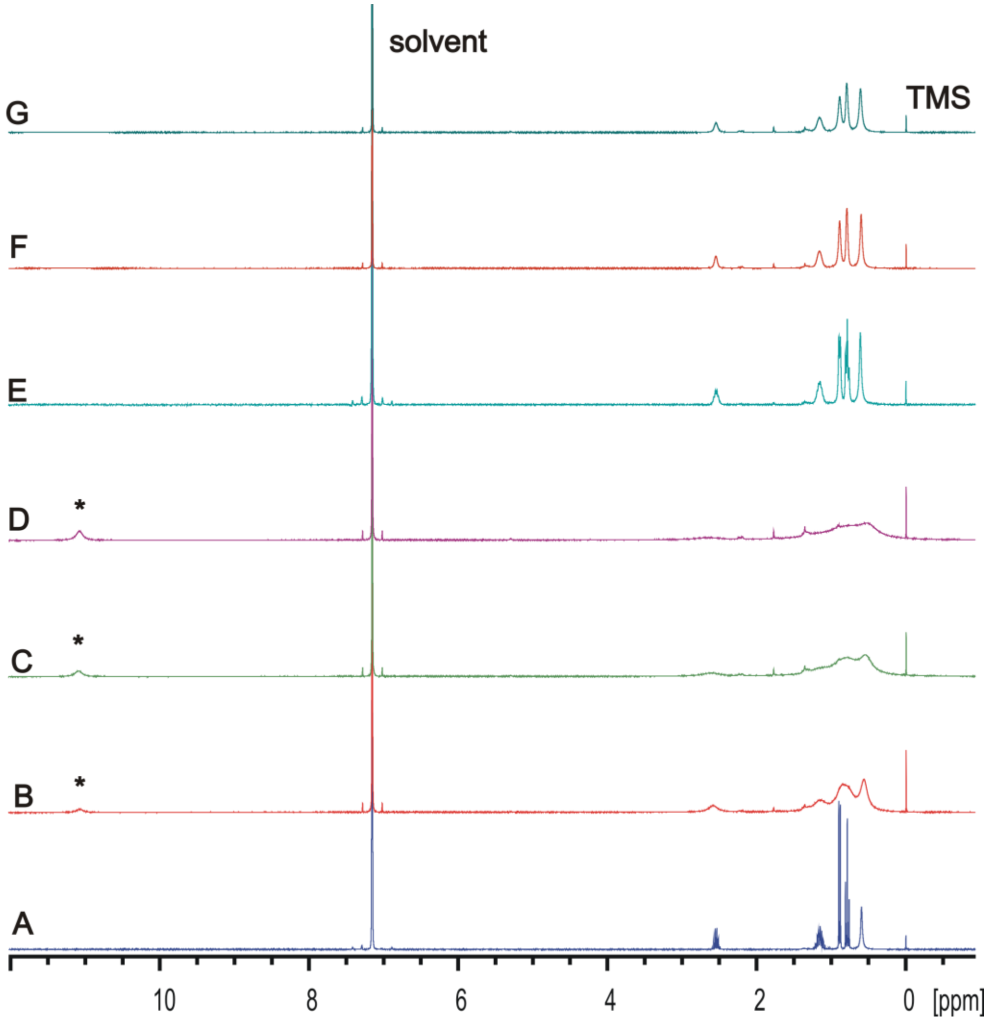

Figure S34.
A – ^{1}H NMR spectrum of 0.033 M solution (*S*)-2-aminobutane in C_6D_6 (300 K, 600 MHz)
B – ^{1}H NMR spectrum of 0.033 M solution (*S*)-2-aminobutane in C_6D_6 after addition of 0.01 equivalents of **[Co$_3$L^{S}$_2$]**
C – ^{1}H NMR spectrum of 0.033 M solution (*S*)-2-aminobutane in C_6D_6 after addition of 0.02 equivalents of **[Co$_3$L^{S}$_2$]**
D – ^{1}H NMR spectrum of 0.033 M solution (*S*)-2-aminobutane in C_6D_6 after addition of 0.03 equivalents of **[Co$_3$L^{S}$_2$]**
E – ^{1}H NMR spectrum of 0.033 M solution (*S*)-2-aminobutane in C_6D_6 after addition of 0.01 equivalents of **[Co$_3$L^{R}$_2$]**
F – ^{1}H NMR spectrum of 0.033 M solution (*S*)-2-aminobutane in C_6D_6 after addition of 0.02 equivalents of **[Co$_3$L^{R}$_2$]**
G – ^{1}H NMR spectrum of 0.033 M solution (*S*)-2-aminobutane in C_6D_6 after addition of 0.03 equivalents of **[Co$_3$L^{R}$_2$]**
Asterisks indicate the signals of the *tert*-butyl groups of the added paramagnetic host.

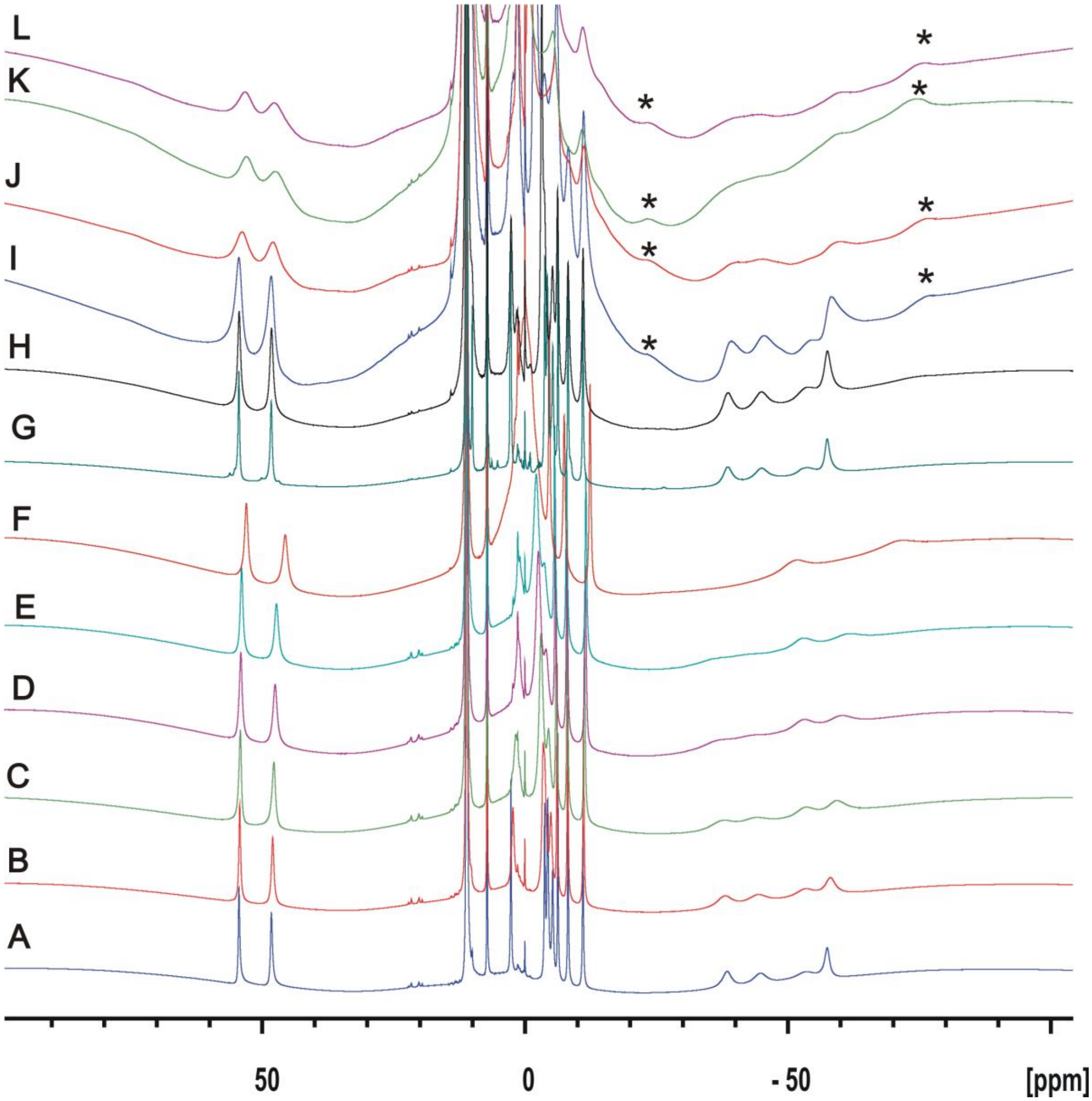

Figure S35. Trace A – paramagnetic range of the ^{1}H NMR spectrum of the 0.004 M solution of [**Co3L*S*2**] in C_6D_6 (300 K, 600 MHz). Traces B – F: the spectra of this solution after addition of 0.4, 1, 1.65, 2.31, and 6.3 equivalents of (*S*)-2-aminobutane, respectively. Trace G – paramagnetic range of the ^{1}H NMR spectrum of the 0.004 M solution of [**Co3L*R*2**] in C_6D_6. Traces H – L: the spectra of [**Co3L*R*2**] solution after addition of 0.4, 1, 1.65, 2.31, and 6.3 equivalents of (*S*)-2-aminobutane, respectively. Asterisks indicate the possible signals of the amine coordinated to Co(II).

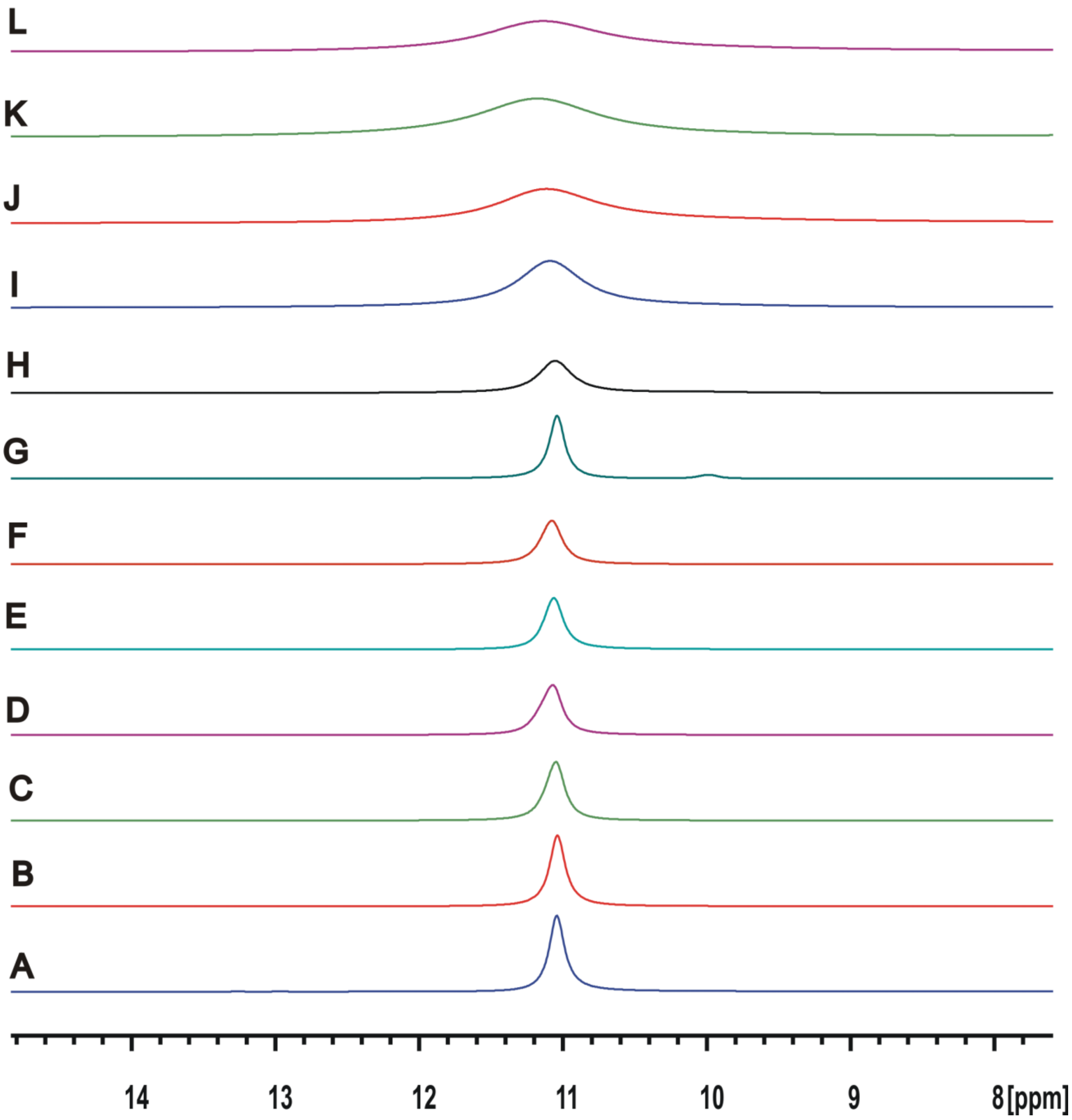

Figure S36. Trace A – the *tert*-butyl ^{1}H NMR signal of the 0.004 M solution of [**$Co_3L^{S}_2$**] in C_6D_6 (300 K, 600 MHz, expanded region of Figure S35). Traces B – F: *tert*-butyl ^{1}H NMR signal of [**$Co_3L^{S}_2$**] solution after addition of 0.4, 1, 1.65, 2.31, and 6.3 equivalents of (*S*)-2-aminobutane, respectively. Trace G – *tert*-butyl ^{1}H NMR signal of the 0.004 M solution of [**$Co_3L^{R}_2$**] in C_6D_6. Traces H – L: the *tert*-butyl ^{1}H NMR signal of [**$Co_3L^{R}_2$**] solution after addition of 0.4, 1, 1.65, 2.31, and 6.3 equivalents of (*S*)-2-aminobutane, respectively.

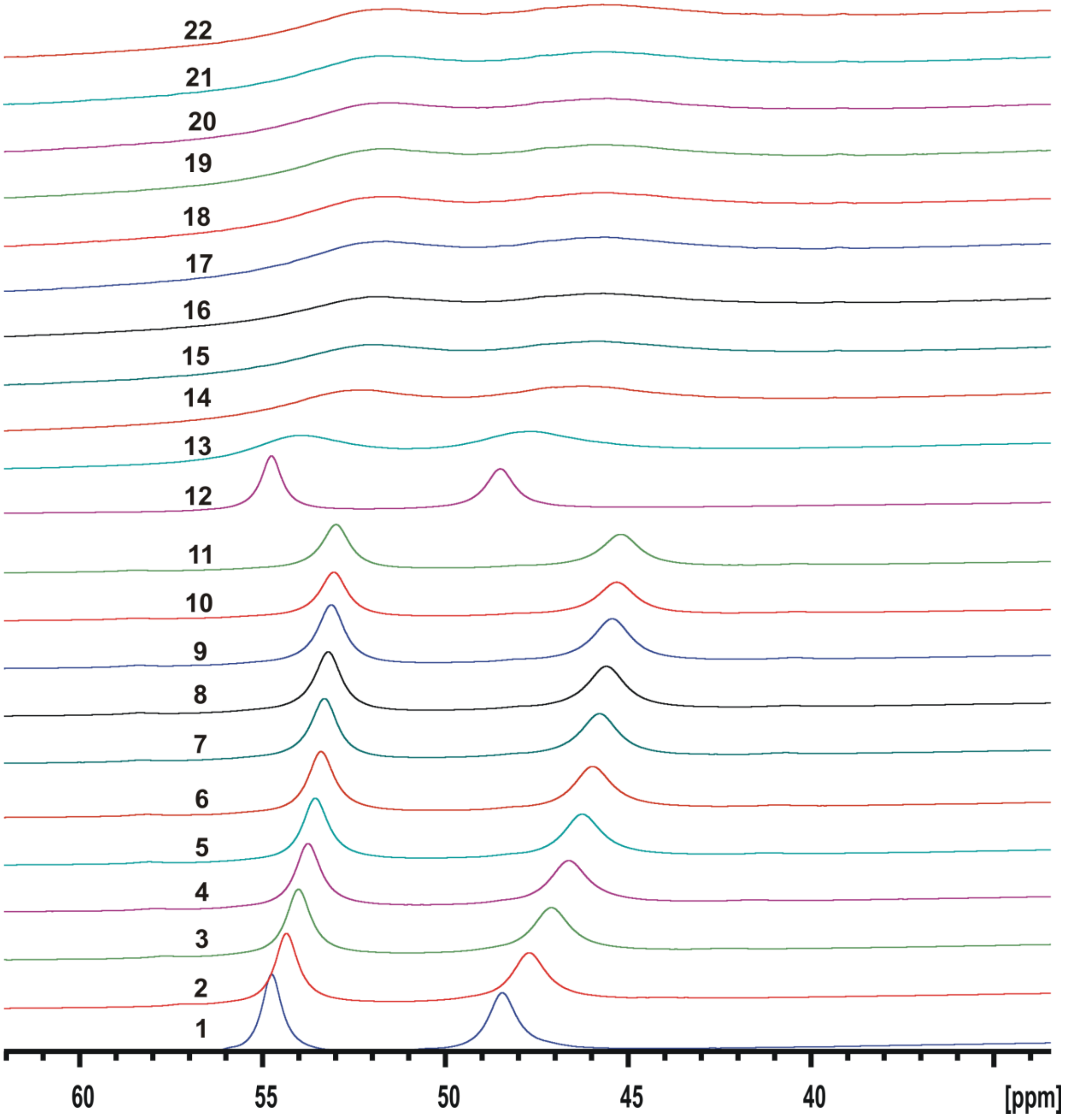

Figure S37.

Traces 1 – 11 ^{1}H NMR signal of [$\mathbf{Co_3L^S{}_2}$] *tert*-butyl group (0.0040 M [$\mathbf{Co_3L^S{}_2}$] solution in C_6D_6, 300 K, 300 MHz) after addition of 0, 0.81, 1.65, 2.47, 3.29, 4.12, 4.94, 5.76, 6.59, 7.41 and 8.24 equivalents of (*S*)-2-aminobutane, respectively. Traces 12 – 22 ^{1}H NMR signal of [$\mathbf{Co_3L^R{}_2}$] *tert*-butyl group (0.0040 M [$\mathbf{Co_3L^R{}_2}$] solution in C_6D_6, 300 K, 300 MHz) after addition of 0, 0.81, 1.65, 2.47, 3.29, 4.12, 4.94, 5.76, 6.59, 7.41 and 8.24 equivalents of (*S*)-2-aminobutane, respectively.

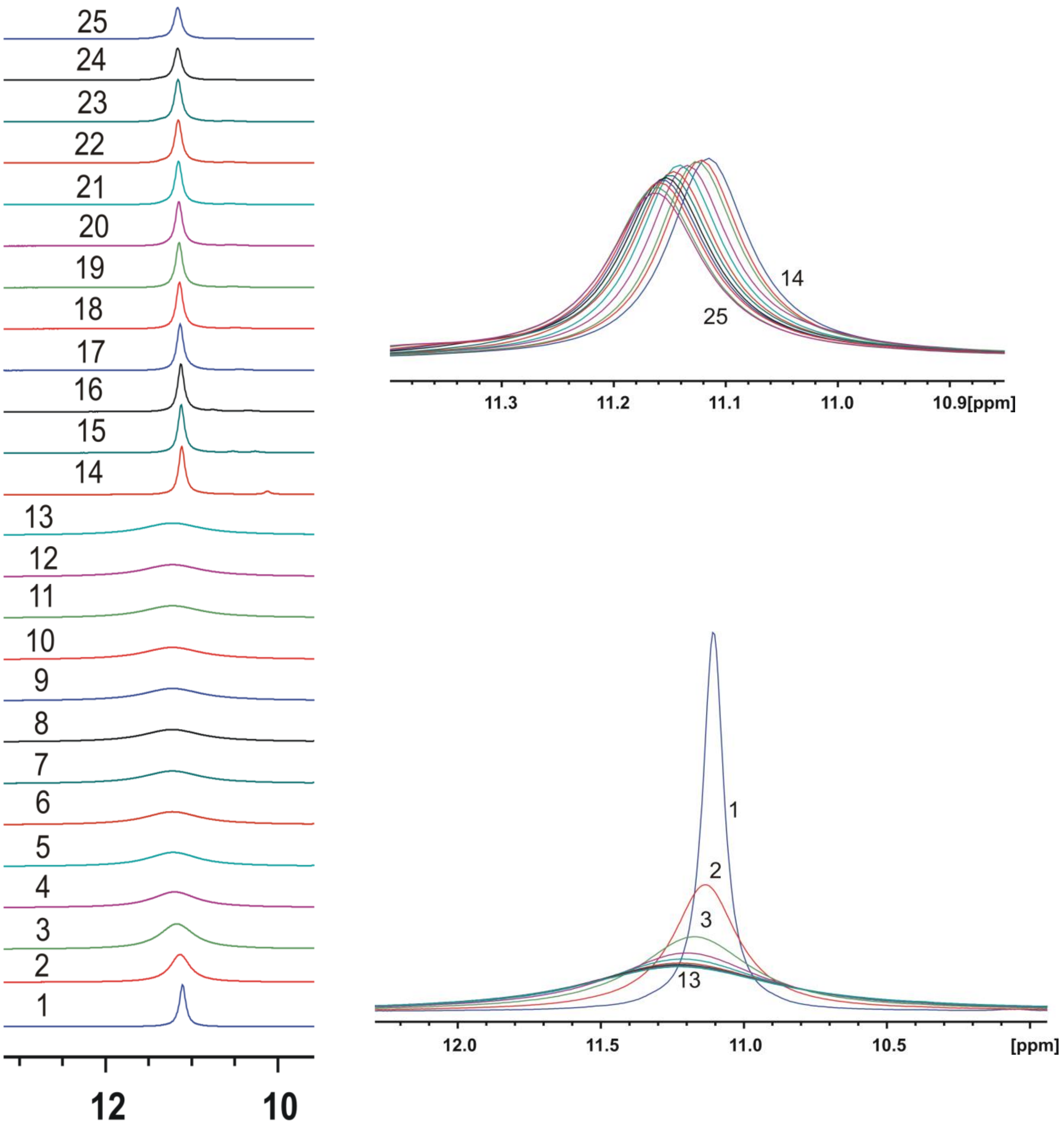

Figure S38. Left panel: Traces 1 – 13 ^{1}H NMR signal of $[\mathbf{Co_3L^{R}_2}]$ *tert*-butyl group (0.0040 M $[\mathbf{Co_3L^{R}_2}]$ solution in C_6D_6, 300 K, 300 MHz) after addition of 0, 0.41, 0.81, 1.24, 1.65, 2.47, 3.29, 4.12, 4.94, 5.76, 6.59, 7.41 and 8.24 equivalents of (*S*)-2-aminobutane, respectively. Traces 14 – 25 ^{1}H NMR signal of $[\mathbf{Co_3L^{S}_2}]$ *tert*-butyl group (0.0040 M $[\mathbf{Co_3L^{S}_2}]$ solution in C_6D_6, 300 K, 300 MHz) after addition of 0, 0.41, 0.81, 1.65, 2.47, 3.29, 4.12, 4.94, 5.76, 6.59, 7.41 and 8.24 equivalents of (*S*)-2-aminobutane, respectively.

Right panel. Overlaid expanded regions of the spectra 1 to 13 and 14 to 25 (note different vertical scales).

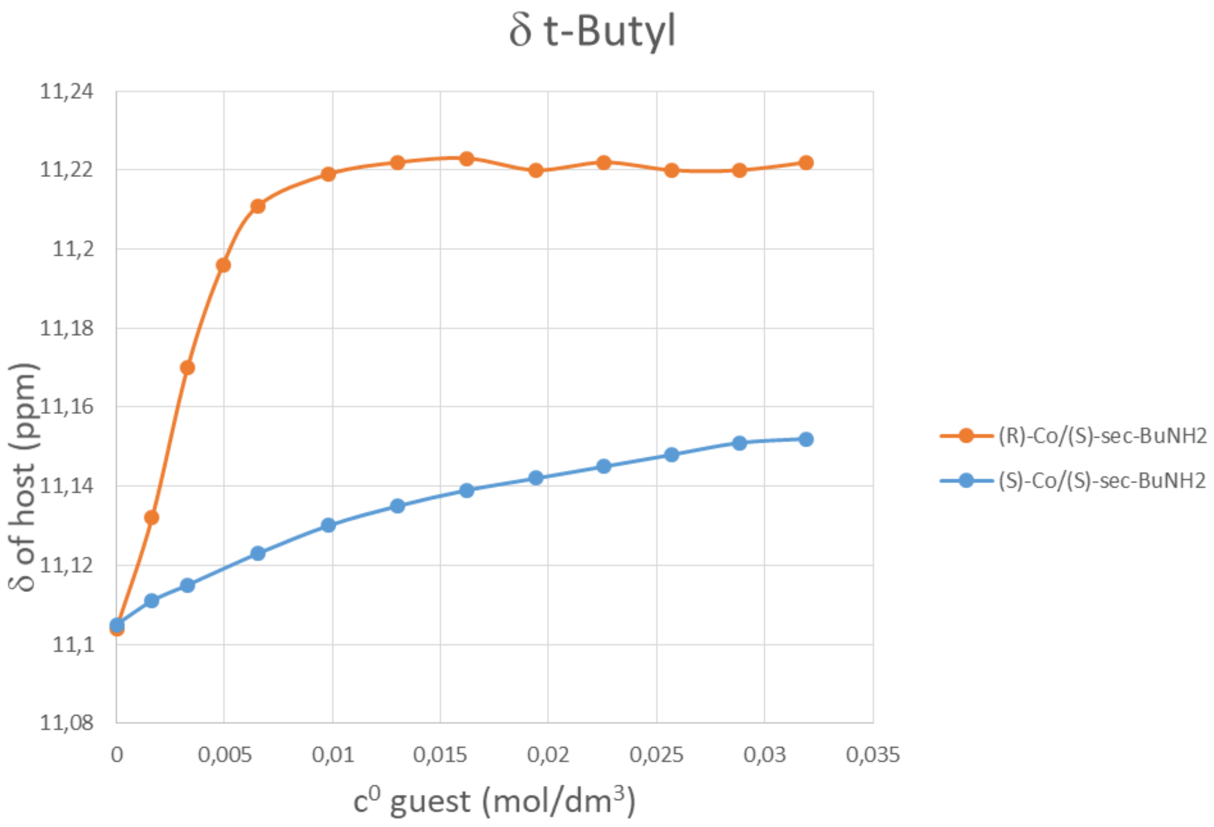

Figure S39. Variations of the chemical shift of the *tert*-butyl signal of the paramagnetic Co(II) cage hosts [**Co$_3$L^R$_2$**] and [**Co$_3$L^S$_2$**] (0.0040 M solutions in C_6D_6, in C_6D_6, 300 K, 300 MHz) during ^{1}H NMR titration experiments with (*S*)-2-aminobutane presented in Figure S38:

Orange-red (R)-Co/(S)-sec-BuNH2 – the (*S*)-2-aminobutane⊂[**Co$_3$L^R$_2$**] pair

Blue (S)-Co/(S)-sec-BuNH2 – the (*S*)-2-aminobutane⊂[**Co$_3$L^S$_2$**] pair

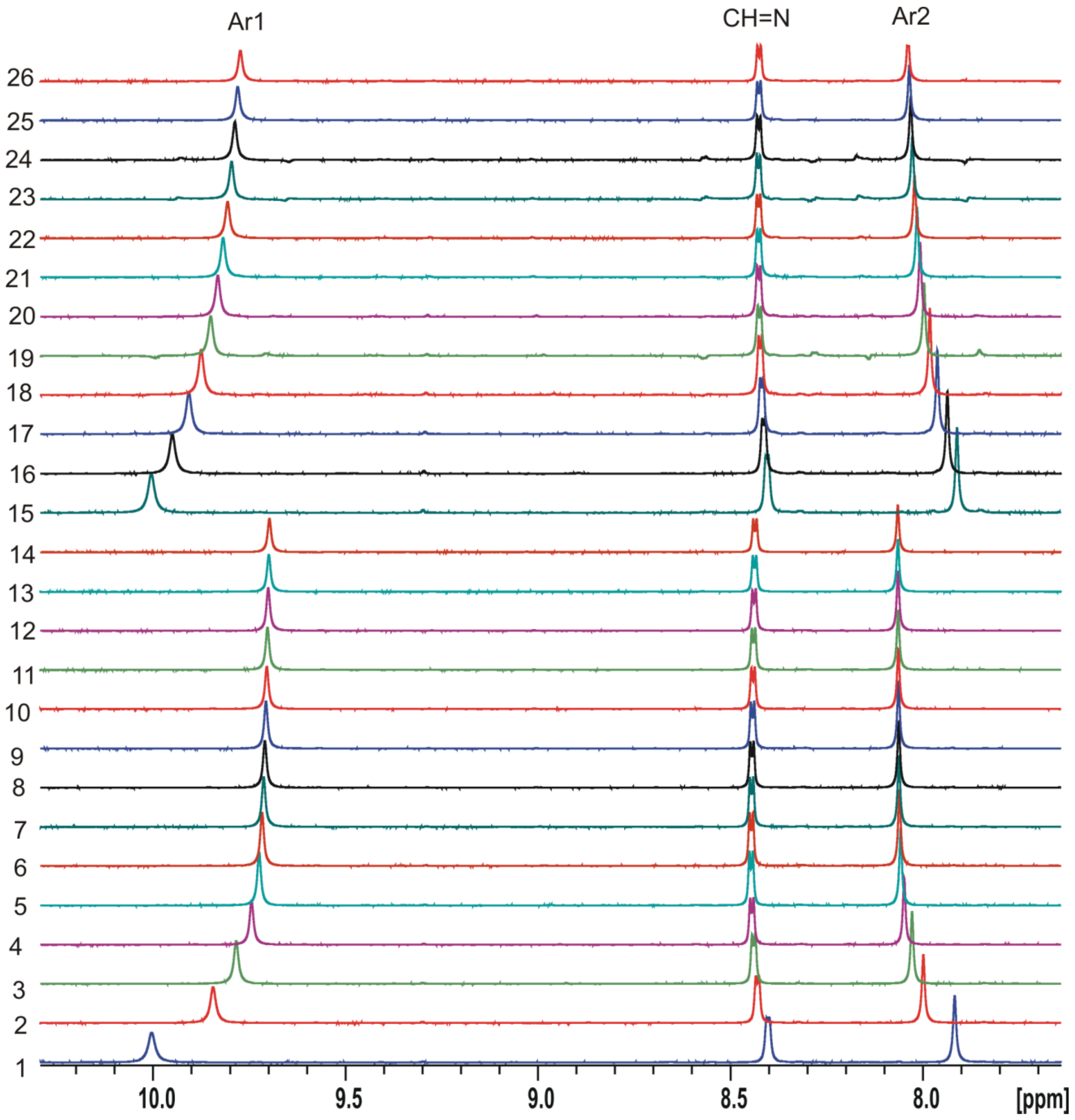

Figure S40. Traces 1 – 14. ^{1}H NMR titration of 0.0040 M [**$Zn_3L^{R}_2$**] solution in C_6D_6, (298 K, 600 MHz) with increasing amount of (*S*)-2-aminobutane: 0, 2.03, 4.06, 8.23, 16.47, 24.71, 32.94, 41.18, 49.41, 57.65, 65.88, 74.12, 82.35 and 90.59 equivalents of added guest, respectively.

Traces 15 – 26. ^{1}H NMR titration of 0.0040 M [**$Zn_3L^{S}_2$**] solution in C_6D_6, (298 K) with increasing amount of (*S*)-2-aminobutane: 0, 8.23, 16.47, 24.71, 32.94, 41.18, 49.41, 57.65, 65.88, 74.12, 82.35 and 90.59 equivalents of added guest, respectively.

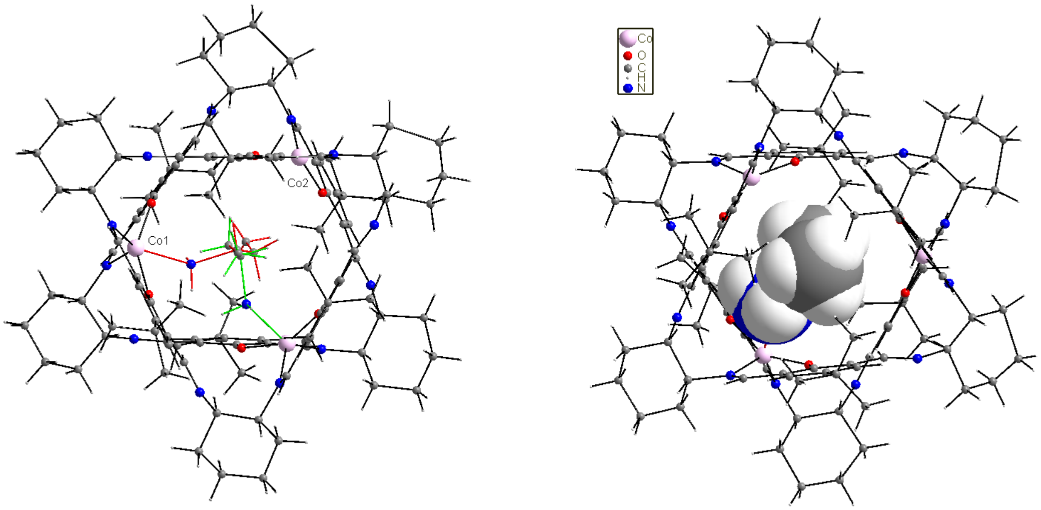

Figure S41. Top views of the $[Co_3L_2(S\text{-}CH_3CH(NH_2)CH_2CH_3)]$ complex. Left – the two orientations of disordered 2-(*R*)-aminobutane indicated in green and red. Right – the 2-(*R*)-aminobutane molecule in spacefill representation

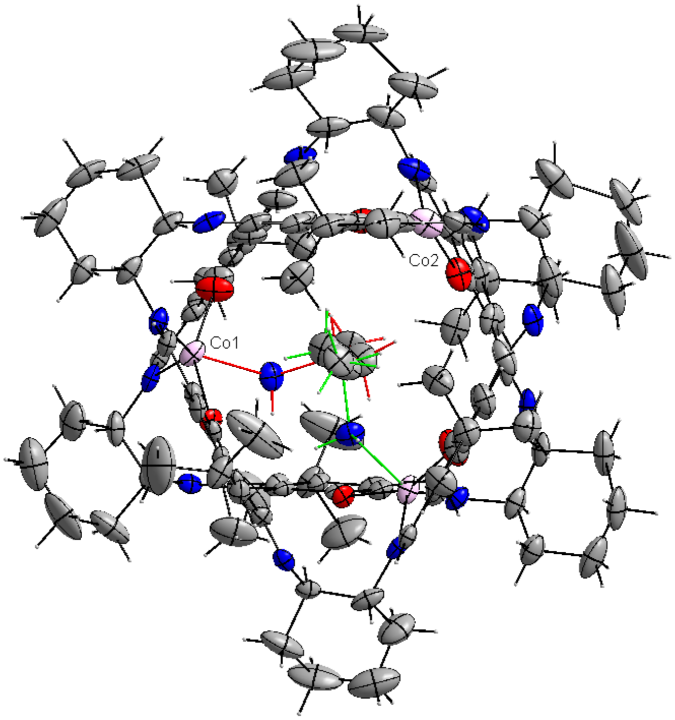

Figure S42. Top view of the $[Co_3L_2(S\text{-}CH_3CH(NH_2)CH_2CH_3)]$ complex with anisotropic displacement parameters.

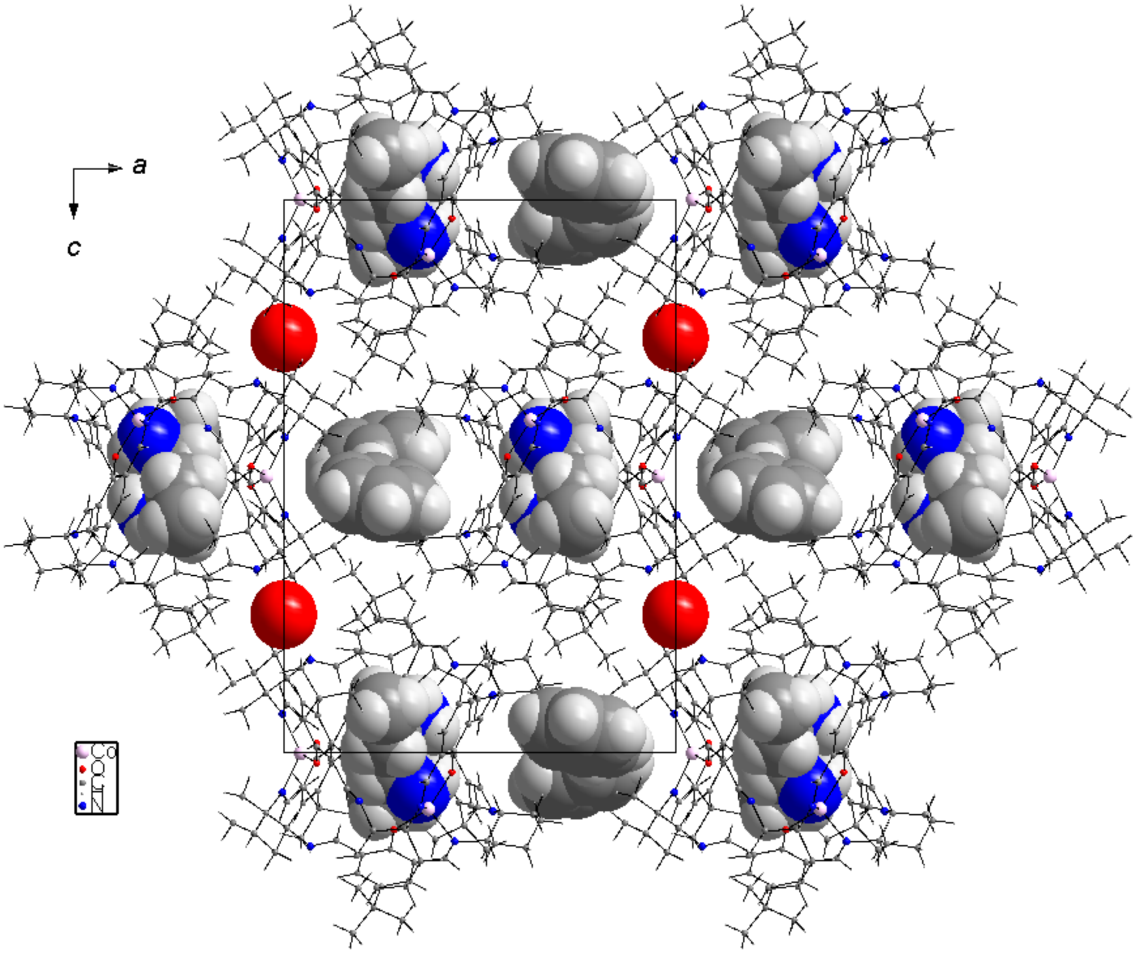

Figure S43. Packing of the $[Co_3L^R_2(S\text{-}CH_3CH(NH_2)CH_2CH_3)]\cdot 2C_6H_6\cdot H_2O$ complex (solvent molecules in spacefill representation).

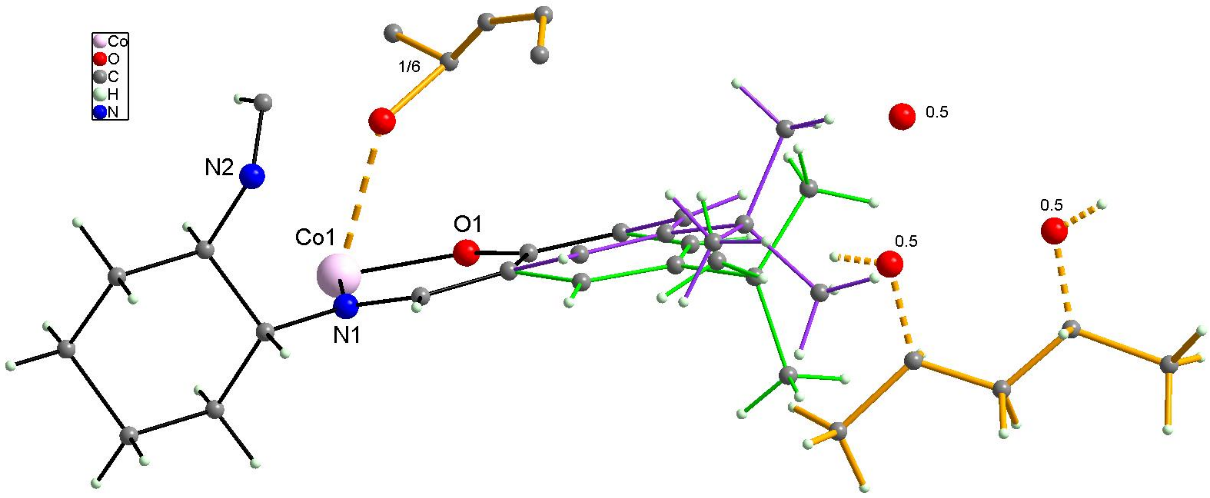

Figure S44. Asymmetric unit of the 2-pentanol derivative of [**$Co_3L^{R}_2$**].

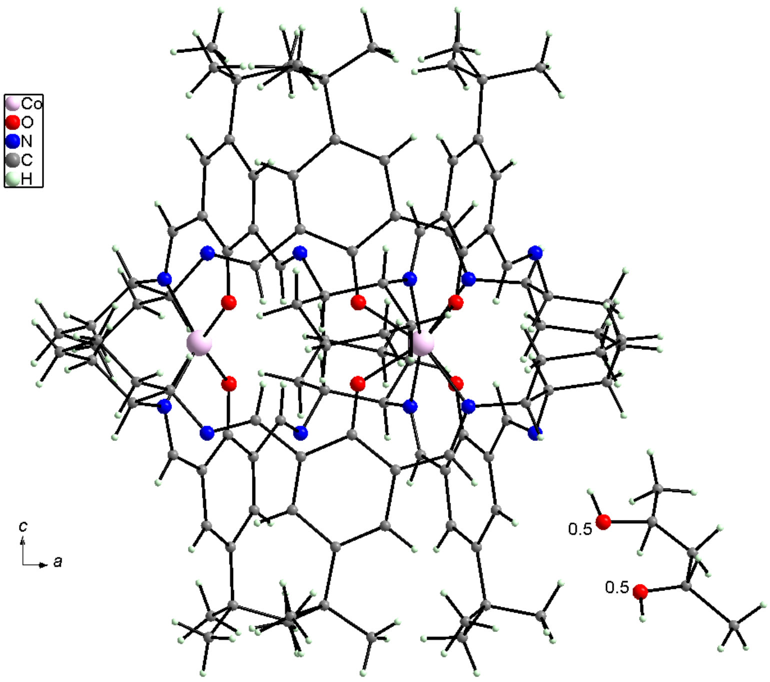

Figure S45. Side view of the 2-pentanol derivative of [**$Co_3L^{R}_2$**]. The 2-pentanol molecules are disordered in two equivalent positions, both with 50% occupancy. The carbon atoms of the 2-penatanol backbone in both orientations overlap.

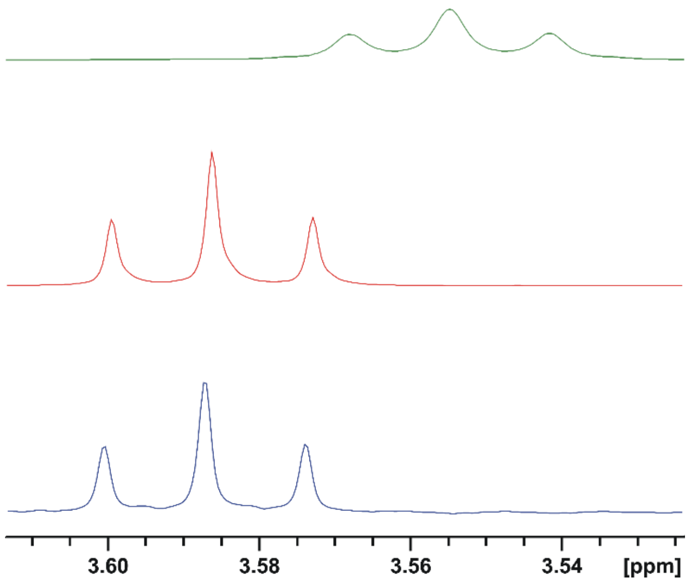

Figure S46. ^{1}H NMR signals of methyl group of 1-propanol: bottom – 0.06 M solution in $CDCl_3$, 298 K, 500 MHz, middle – after addition of 0.01 equivalents of [$\mathbf{Zn_3L^{\mathit{R}}_2}$], top – after addition of 0.01 equivalents of [$\mathbf{Co_3L^{\mathit{R}}_2}$].

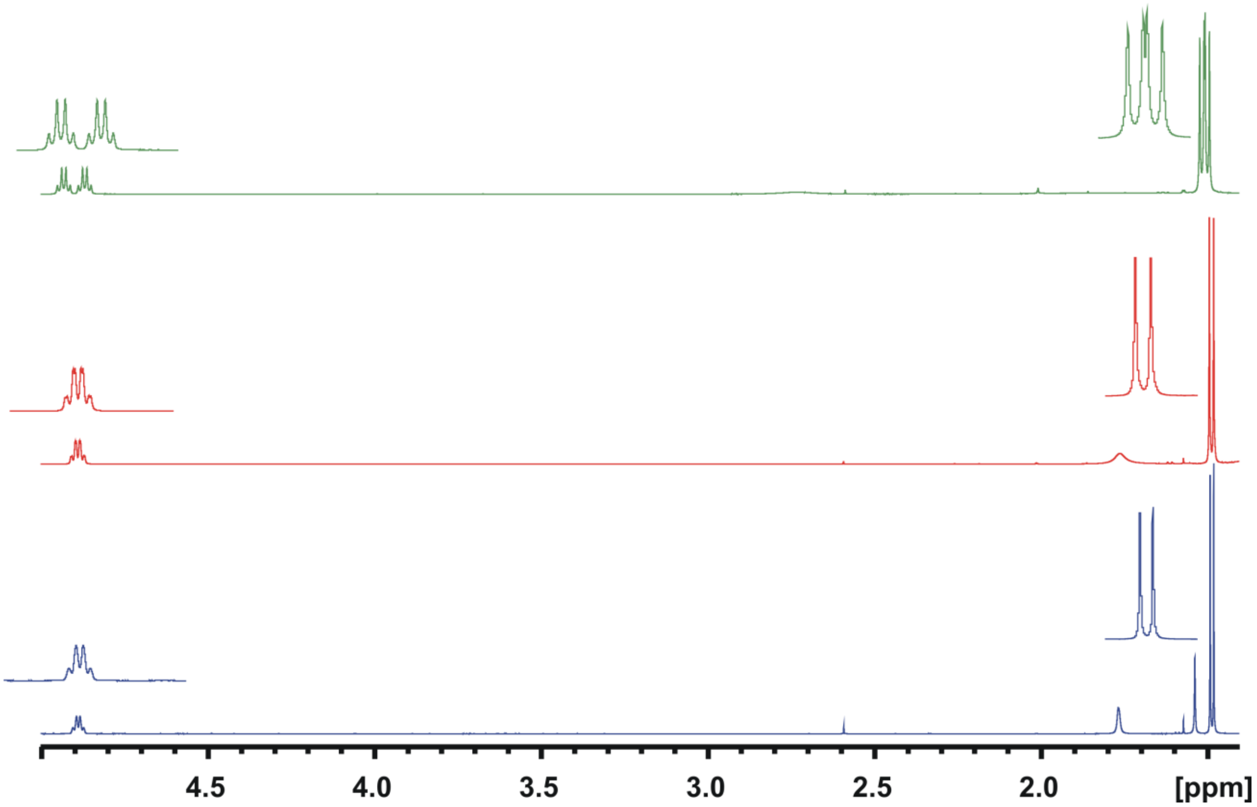

Figure S47. ^{1}H NMR spectra (298 K, 500 MHz, 16 scans) of racemic 1-phenylethanol: bottom – 0.04 M solution in $CDCl_3$, middle – after addition of 0.004 equivalents [$\mathbf{Co_3L^{\mathit{R}}_2}$], top – after addition of 0.05 equivalents of [$\mathbf{Co_3L^{\mathit{R}}_2}$].

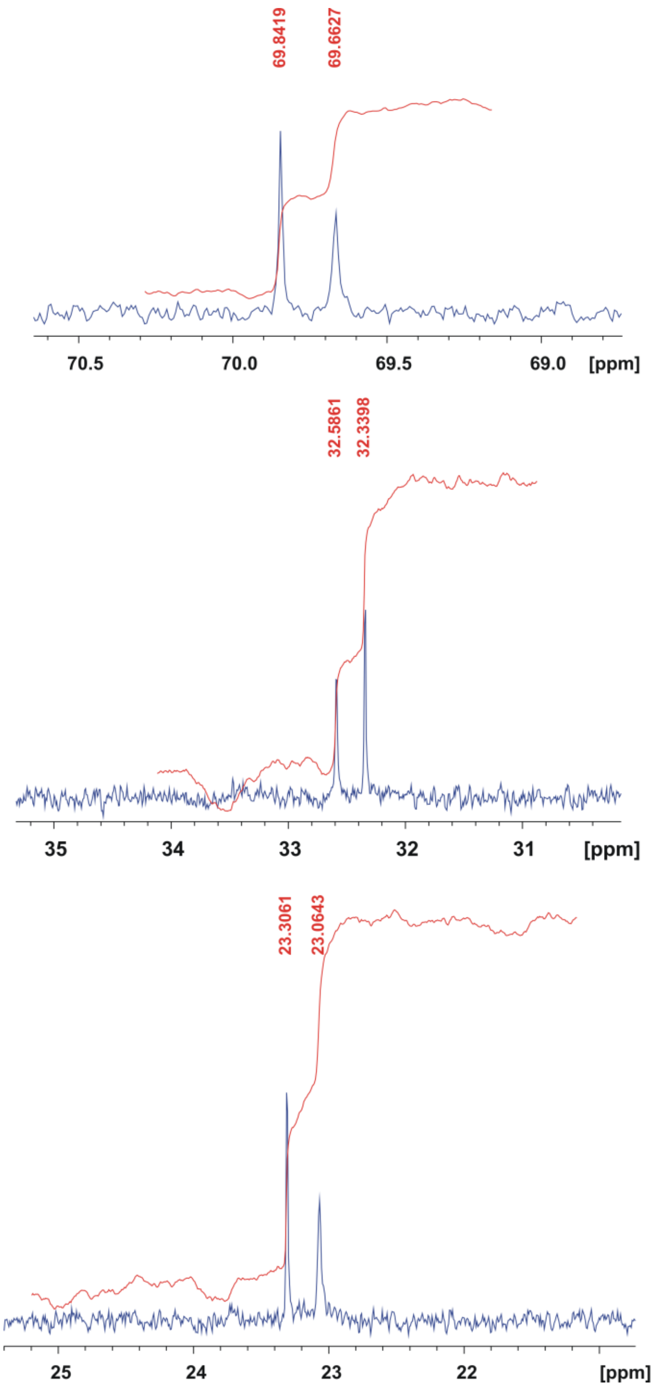

Figure S48. Integration of expanded regions of the ^{13}C-{^{1}H} NMR spectrum of racemic 2-butanol (298 K, 125 MHz, 7168 scans, 0.036 M solution in $CDCl_3$) after addition of 0.028 equivalents of [**$Co_3L^{S}{}_2$**].

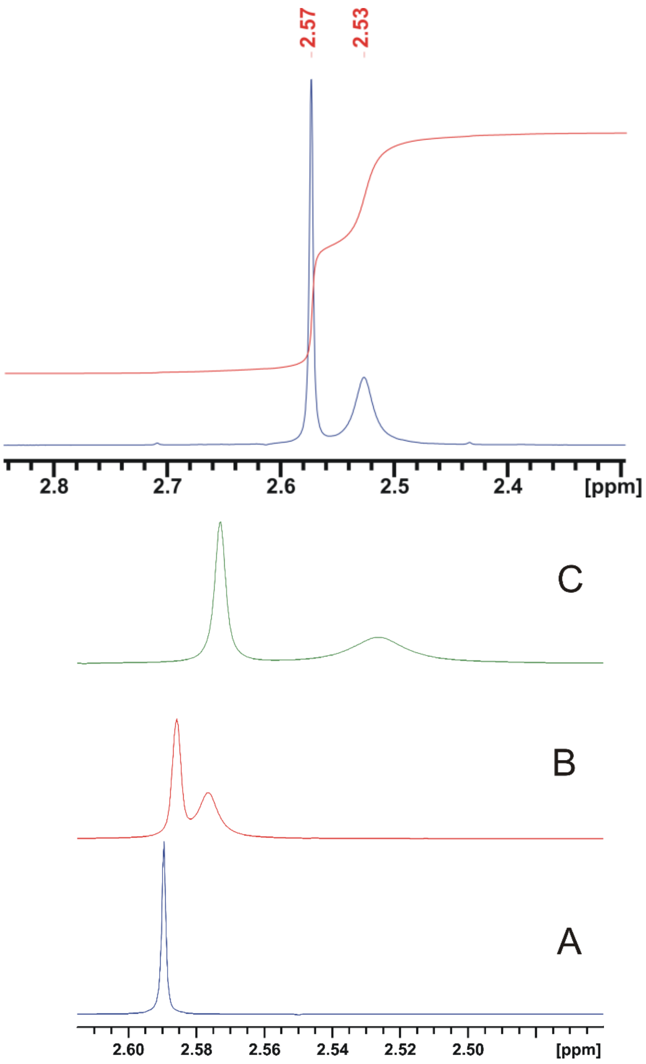

Figure S49. ^{1}H NMR signals of methyl group of DMSO (0.07 M solution in $CDCl_3$, 298 K, 500 MHz) after addition of increasing amounts of **[Co$_3$L^{R}$_2$]**. Traces A, B, C – 0, 0.0024 and 0.01 equivalents of added **[Co$_3$L^{R}$_2$]**, respectively. The split prochiral methyl groups are of the same intensity as shown by the integration of trace C on top of the figure.

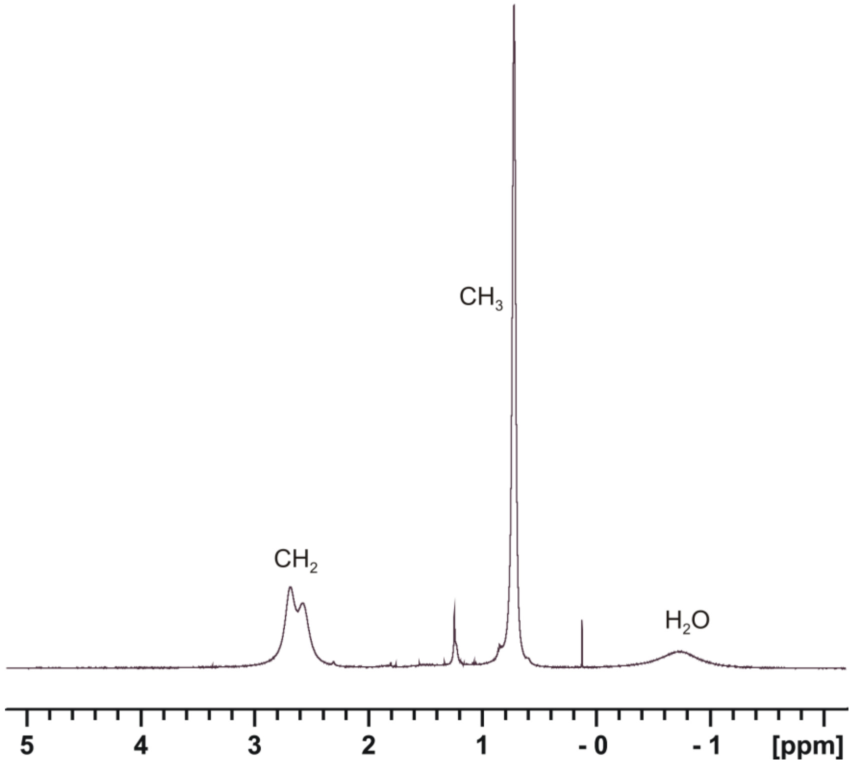

Figure S50. ^{1}H NMR spectrum of ethanol (0.1 M solution in $CDCl_3/C_6D_6$) after addition of 0.05 equivalents of [**Co$_3$L^{R}$_2$**].

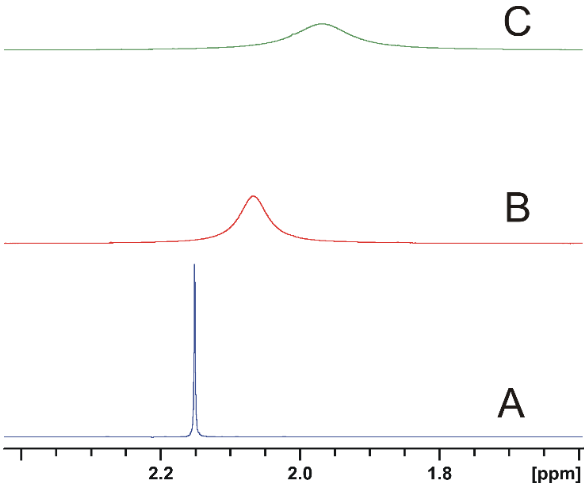

Figure S51. ^{1}H NMR signal of methyl group of acetone (0.04 M solution in $CDCl_3$) after addition of increasing amounts of [**Co$_3$L^{R}$_2$**]. Traces A, B, C – 0, 0.004 and 0.008 equivalents of added [**Co$_3$L^{R}$_2$**], respectively.

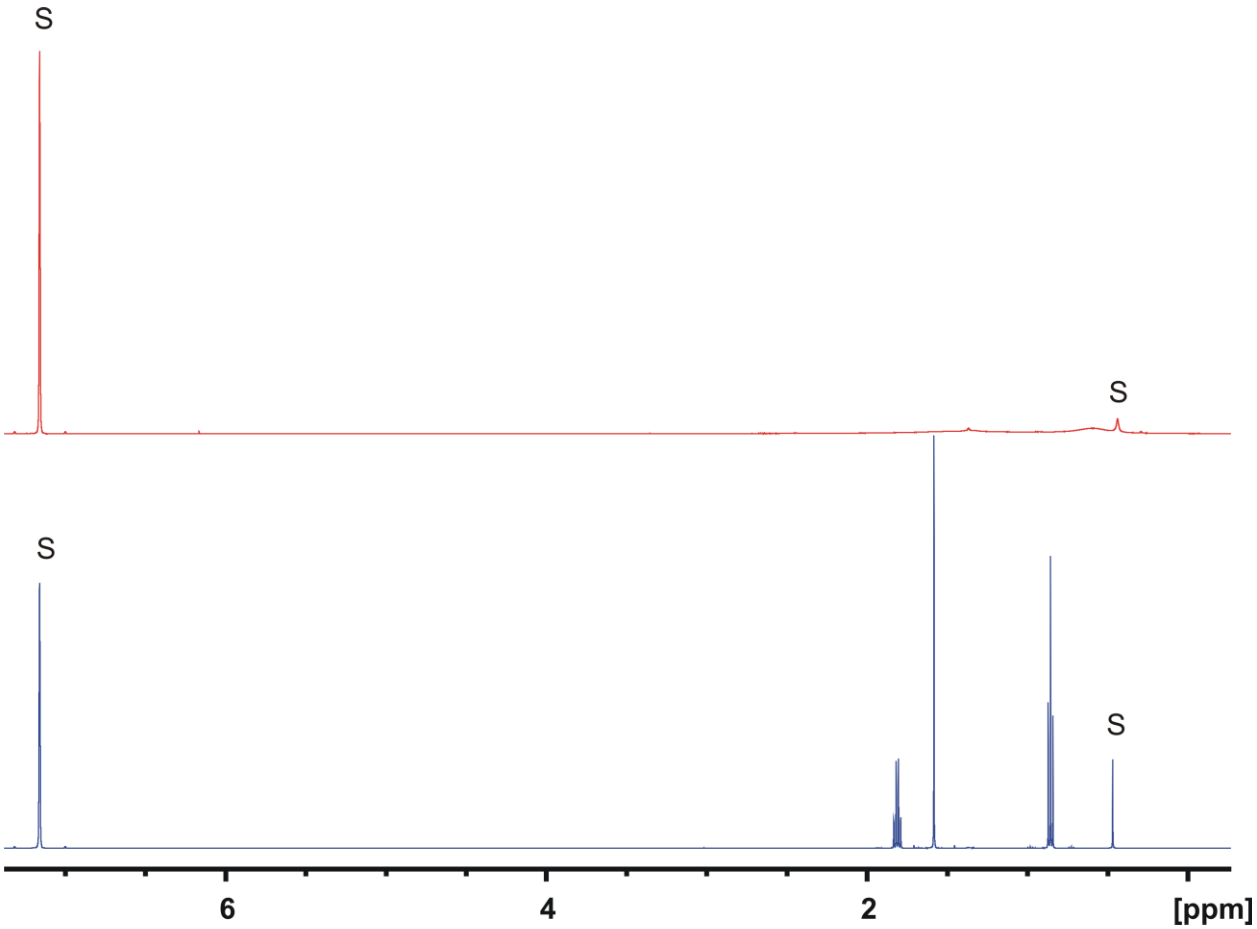

Figure S52. ^{1}H NMR spectrum of 2-butanone (0.05 M solution in C_6D_6, 300K, 500MHz) before (bottom) and after (top) addition of 0.0037 equivalents of added [**$Co_3L^{R}{}_2$**], respectively (s – residual solvent and water signal).

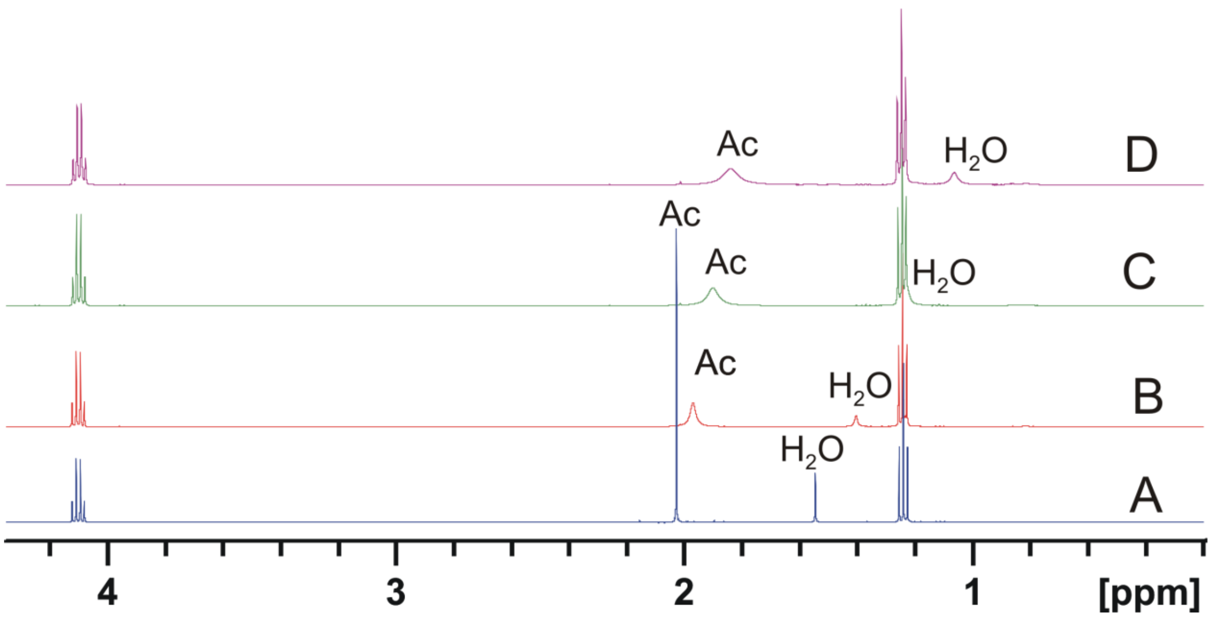

Figure S53. NMR titration of of ethyl acetate (0.03 M solution in $CDCl_3$, 298 K, 500 MHz) with increasing amount of **[$Co_3L^R{}_2$]**. Traces A – D correspond to 0, 0.005, 0.01 and 0.015 equivalents of added Co(II) complex, respectively. While the signal of the ethyl group is practically unaffected, the signal of the methyl group of acetate (Ac) is clearly broadened and shifted in accord with placement of this fragment of the molecule within the cavity of paramagnetic cage.

(a)

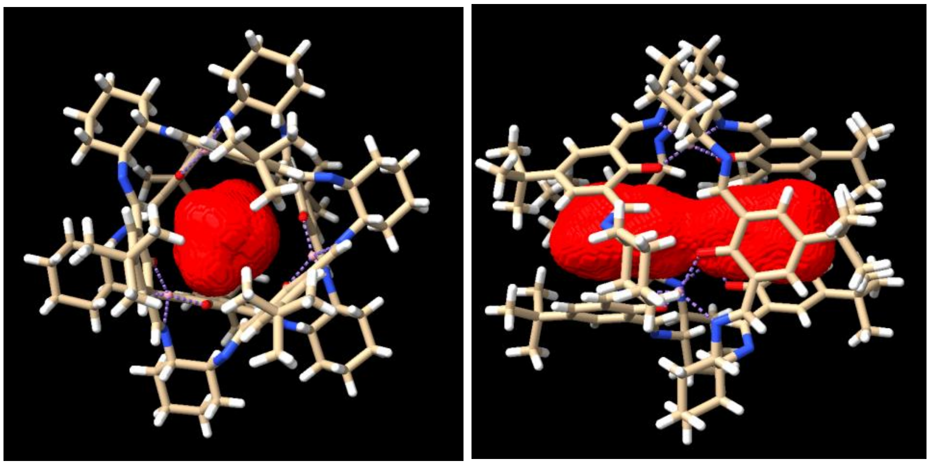

(b)

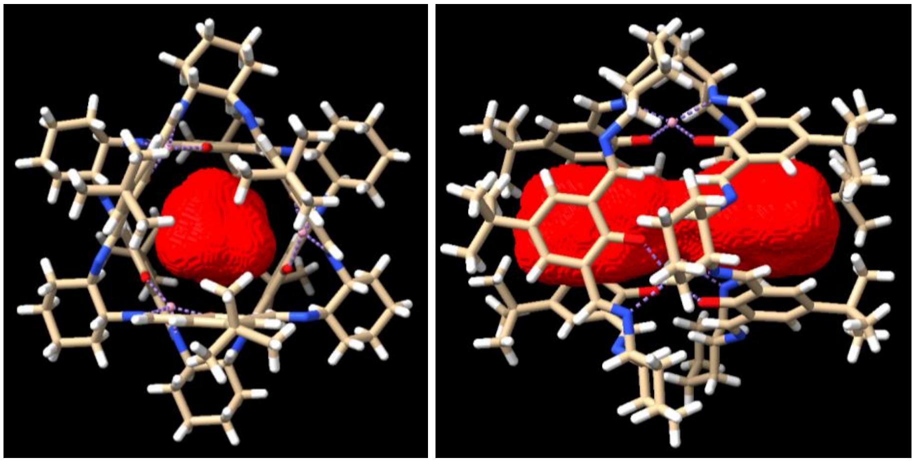

Figure S54. Top and side views of the cavity in complexes **$[Co_3L^{S}_2]\cdot 8(CH_3OH)\cdot 4(H_2O)$ - 1** (a) and **$[Co_3L^{R}_2]\cdot 2(C_6H_6)\cdot 3(H_2O)$ - 3** (b) made using MoloVol Ver. 1.1.1 [5] and ChimeraX ver. 1.8 programs [6].

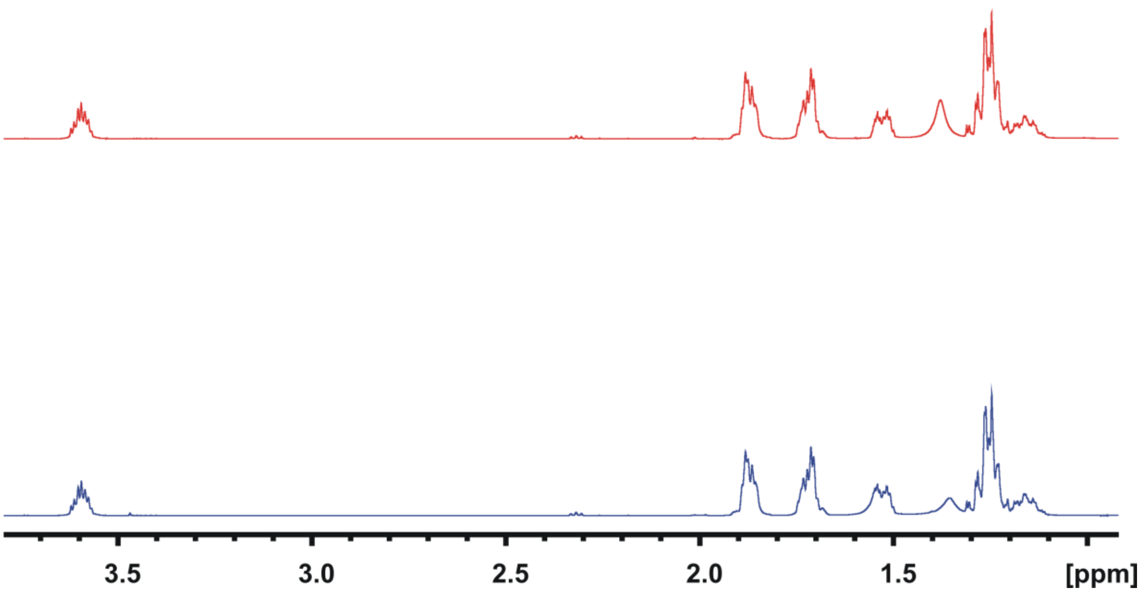

Figure S55. ^{1}H NMR spectrum of cyclohexanol (0.05 M solution in $CDCl_3$, 300K, 500MHz) before (bottom) and after (top) addition of 0.0036 equivalents of added [$\mathbf{Co_3L^{R}_2}$].

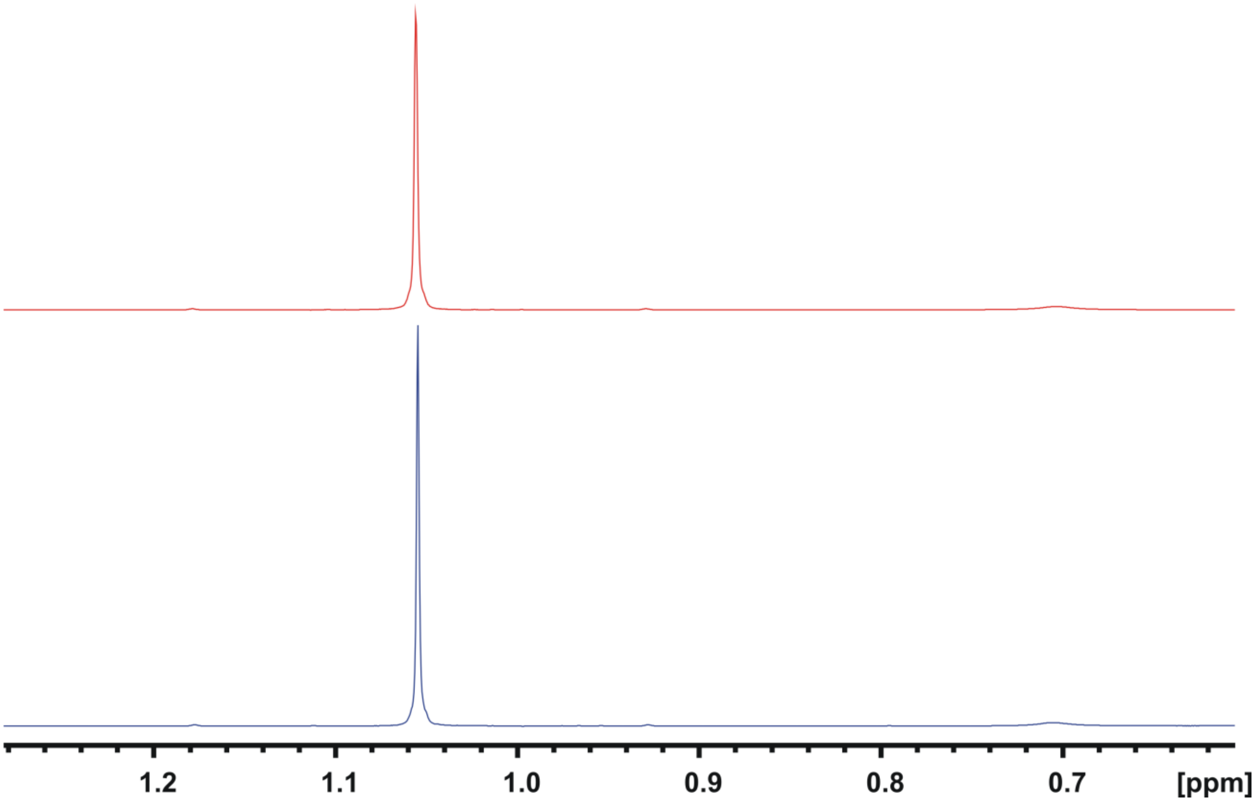

Figure S56. ^{1}H NMR spectrum of *tert*-butanol (0.15 M solution in C_6D_6, 300K, 500MHz) before (bottom) and after (top) addition of 0.003 equivalents of added [$\mathbf{Co_3L^{R}_2}$], respectively.

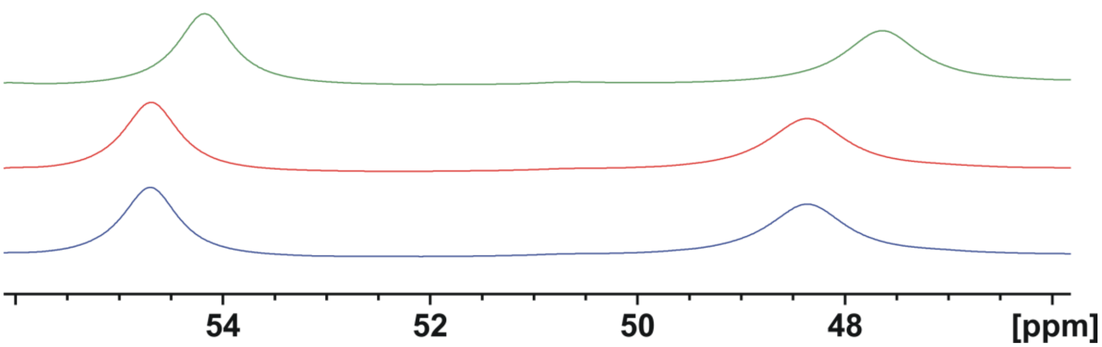

Figure S57. Regions of 1H NMR spectra of 0.004 M solution of **[Co$_3$L$^{S}_2$]** (bottom), 0.004 M solution of **[Co$_3$L$^{S}_2$]** after addition of xxx equivalents of *t*-butanol (middle) and 0.004 M solution of **[Co$_3$L$^{S}_2$]** after addition of xxx equivalents of 1-butanol.

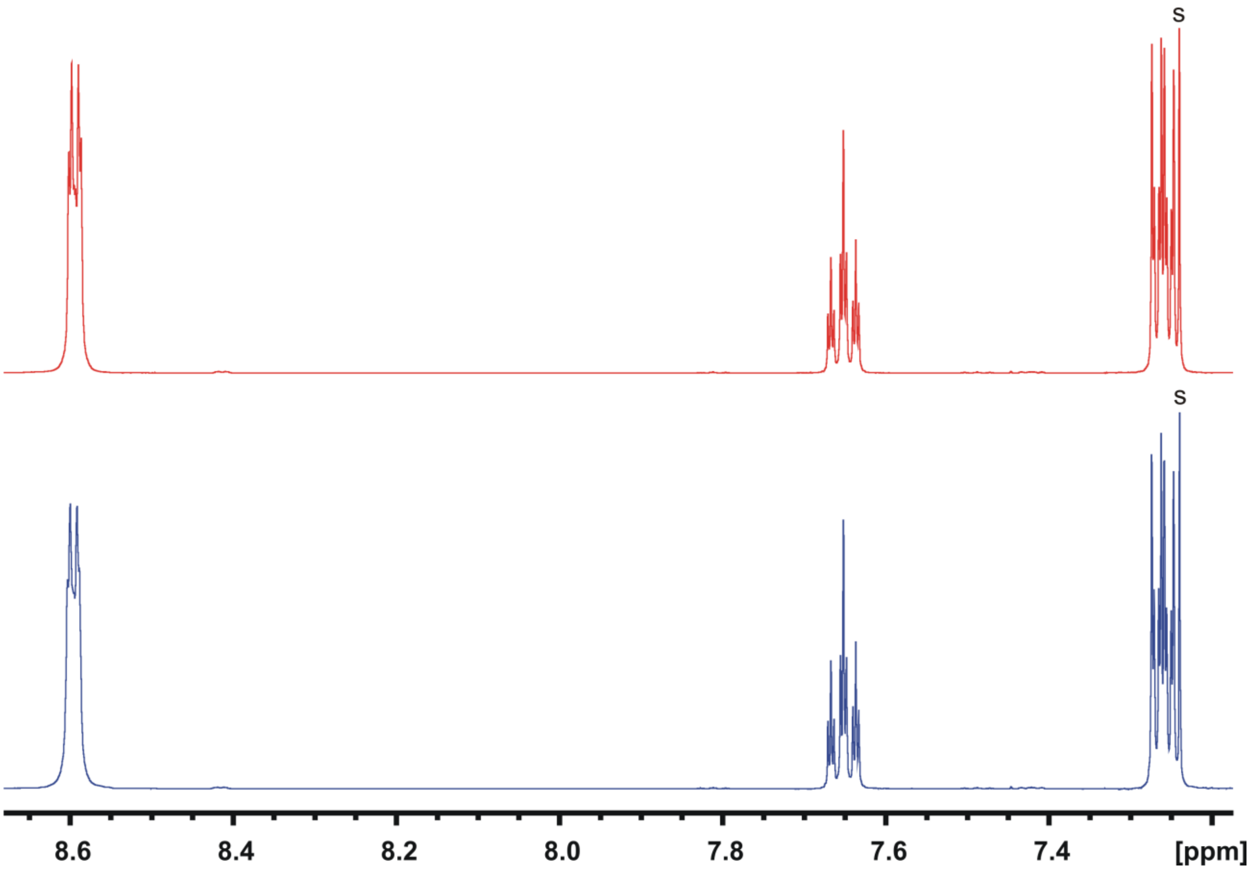

Figure S58. ^{1}H NMR spectrum of pyridine (0.065 M solution in CDCl$_3$, 300K, 500 MHz) before (bottom) and after (top) addition of 0.003 equivalents of added **[Co$_3$L$^{R}_2$]**.

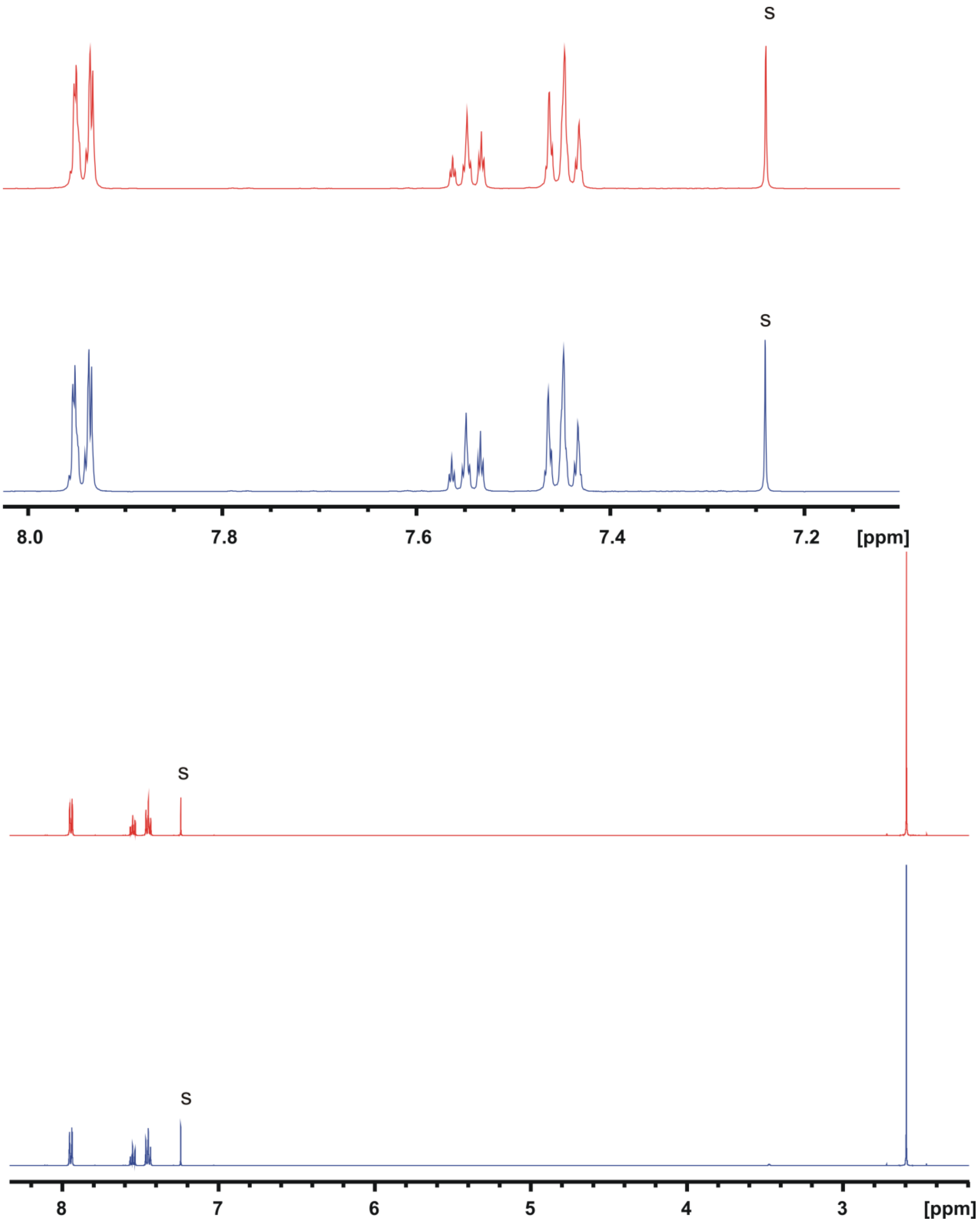

Figure S59. ^{1}H NMR spectrum of acetophenone (0.045 M solution in $CDCl_3$, 300K, 500 MHz) before (blue) and after (red) addition of 0.004 equivalents of added [$\mathbf{Co_3L^{\mathit{R}}{}_2}$], (s – residual solvent signal).

References

[1] Gupta, S. K.; Murugavel, R. *Chem. Commun.* **2018**, *54*, 3685-3696.

[2] Zabala-Lekuona, A.; Seco, J. M.; Colacio, E. *Coordination Chemistry Reviews* **2021**, *441*.

[3] Vieru, V.; Gómez-Coca, S.; Ruiz, E.; Chibotaru, L. F. *Angew. Chem. Int. Ed.* **2024**, *63*, e202303146

[4] Sahu, P. K.; Kharel, R.; Shome, S.; Goswami, S.; Konar, S. *Coord. Chem. Review.* **2023**, *475*, 214871.

[5] Maglic, J.B.; Lavendomme, R.; *J. App. Cryst*. **2022**, *55*, 1033-1044.

[6] Meng, E.C.; Goddard, T.D.; Pettersen, E.F.; Couch, G.S.; Pearson, Z.J.; Morris, J.H.; Ferrin, T.E.; UCSF ChimeraX: Tools for structure building and analysis. *Protein Sci*. **2023**, *32(11)*, e4792.
